# Subcellular Partitioning of Protein Tyrosine Phosphatase 1B to the Endoplasmic Reticulum and Mitochondria Depends Sensitively on the Composition of Its Tail Anchor

**DOI:** 10.1371/journal.pone.0139429

**Published:** 2015-10-02

**Authors:** Julia Fueller, Mikhail V. Egorov, Kirstin A. Walther, Ola Sabet, Jana Mallah, Markus Grabenbauer, Ali Kinkhabwala

**Affiliations:** 1 Department of Systemic Cell Biology, Max Planck Institute of Molecular Physiology, Otto-Hahn-Strasse 11, 44227, Dortmund, Germany; 2 Center for Molecular Biology (ZMBH), DKFZ-ZMBH Alliance, Heidelberg University, Im Neuenheimer Feld 282, 69120, Heidelberg, Germany; 3 Institute of Anatomy and Cell Biology, Heidelberg University, Im Neuenheimer Feld 307, 69120, Heidelberg, Germany; Institute for Nutritional Sciences, CHINA

## Abstract

The canonical protein tyrosine phosphatase PTP1B is an important regulator of diverse cellular signaling networks. PTP1B has long been thought to exert its influence solely from its perch on the endoplasmic reticulum (ER); however, an additional subpopulation of PTP1B has recently been detected in mitochondria extracted from rat brain tissue. Here, we show that PTP1B’s mitochondrial localization is general (observed across diverse mammalian cell lines) and sensitively dependent on the transmembrane domain length, C-terminal charge and hydropathy of its short (≤35 amino acid) tail anchor. Our electron microscopy of specific DAB precipitation revealed that PTP1B localizes via its tail anchor to the outer mitochondrial membrane (OMM), with fluorescence lifetime imaging microscopy establishing that this OMM pool contributes to the previously reported cytoplasmic interaction of PTP1B with endocytosed epidermal growth factor receptor. We additionally examined the mechanism of PTP1B’s insertion into the ER membrane through heterologous expression of PTP1B’s tail anchor in wild-type yeast and yeast mutants of major conserved ER insertion pathways: In none of these yeast strains was ER targeting significantly impeded, providing in vivo support for the hypothesis of spontaneous membrane insertion (as previously demonstrated in vitro). Further functional elucidation of the newly recognized mitochondrial pool of PTP1B will likely be important for understanding its complex roles in cellular responses to external stimuli, cell proliferation and diseased states.

## Introduction

The founding member of its family, protein tyrosine phosphatase 1B (PTP1B) [1,2] (the protein product of the gene PTPN1 [3]) is an important regulator of phosphotyrosine signaling in mammalian cells through its dephosphorylation of a range of substrates [4], including the receptors for insulin, leptin and epidermal growth factor (EGF) and their downstream substrates; the tyrosine kinases JAK2 and c-Src; and the tyrosine phosphatase SHP2. PTP1B expression has been detected in several tissues in different mammals [5] and has been proposed as an important target for treatment of diabetes, obesity and cancer [6]. Its general role, particularly in cancer cell signaling, appears to be complex [7].

PTP1B is expressed as two separate splice variants [8], the first identified in rat brain tissue [9] with the second later identified in human placenta [5]. These variants differ only in their terminal amino acids, with the first variant ending in VCFH and the second in FLFNSNT. Unlike the stably expressed FLFNSNT variant, expression of the VCFH variant is highly regulated by growth factor [8]. The subcellular localization of both variants appears to be similar [8]. Both variants consist of an N-terminal catalytic domain and a C-terminal tail anchor [10]. A substrate “trapping mutant” of its catalytic domain [11], the D181A mutant PTP1B^D/A^, has long provided a useful tool for understanding its catalytic mechanism as well as for enhanced detection of its interactions with substrates. PTP1B’s short (≤35 amino acid) C-terminal tail anchor was previously reported to localize it to the membrane of the endoplasmic reticulum (ER) [10,12]. PTP1B’s insertion into the ER has been shown in vitro to proceed in the absence of membrane proteins [13] and in vivo to at least partially involve the chaperones Hsp40/Hsc70 [14], the latter in agreement with other tail anchor proteins [15]. While these studies have already shed light on important aspects of PTP1B’s ER insertion, other factors might contribute as well to increase its insertion efficiency in vivo, including in particular the guided entry of tail anchor proteins (GET/TRC40) pathway [16–23] or other chaperones. More general insertion pathways such as the post-translational mode of the signal recognition particle (SRP) pathway [24] or the Sec62/63 pathway [25,26] might also contribute. The relative importance of these different pathways on PTP1B’s insertion efficiency in vivo is unknown. In addition to the two different splice variants, further diversity of PTP1B, which might also affect its subcellular targeting, is generated through several post-translational modifications that are known to activate or inhibit it [4], including phosphorylation (on multiple serines and tyrosines), oxidation, sumoylation and proteolysis (calpain cleavage).

The subcellular distribution of PTP1B has also been the subject of several prior studies. The restriction of PTP1B to the ER has been argued as a means for regulating its interaction with plasma membrane (PM) versus endocytosed fractions of EGFR [27]. A subcellular gradient of the activity of PTP1B has been proposed to account for observations of its interactions with an artificial substrate [28]. The specific roles of ER-bound PTP1B at adhesions sites [29,30] and cell-cell junctions [31] have also been explored. These investigations highlight potentially important and distinct physiological roles for PTP1B subpopulations distributed across the cell.

Intriguingly, the VCFH isoform of PTP1B has recently been detected within mitochondria extracted from rat brain tissue [32,33] (rats express only this isoform). PTP1B’s potential presence at the mitochondria could be important for regulation of the mitochondrial phosphotyrosine proteome [34], with possible targets including several enzymes in the electron transport chain [35], Src family kinases that localize to the mitochondria [33,36–40], or other well-established substrates of PTP1B that have also been detected at the mitochondria like the EGF receptors ErbB1 [41,42] and ErbB2 [43] and the tyrosine phosphatase SHP2 [33,40,44]. The generality of PTP1B’s mitochondrial localization, though, should be confirmed first (in particular, for other cell lines), before further speculating on how it may reach this organelle or how its interaction there with known or only putative substrates might affect basic mitochondrial functions.

In this study, we report the following novel findings regarding PTP1B’s tail-anchor-mediated targeting to the ER and mitochondria and its role in mitochondrial signaling: (1) Endogenous PTP1B localizes to the mitochondria in multiple mammalian cell lines, (2) PTP1B localizes to the outer mitochondrial membrane via its tail anchor, (3) Heterologous expression of PTP1B’s tail anchor in yeast reveals a potential minor role of the GET/TRC40 pathway in ER insertion, (4) Subcellular partitioning of PTP1B’s tail anchor is highly sensitive to its exact composition and (5) FLIM reveals an interaction of PTP1B with EGFR at the outer mitochondrial membrane. In the final discussion section, our specific results on PTP1B are placed within the context of general tail anchor targeting and mitochondrial phosphotyrosine signaling.

## Results

### Endogenous PTP1B Localizes to the Mitochondria in Multiple Mammalian Cell Lines

**Fig 1:**
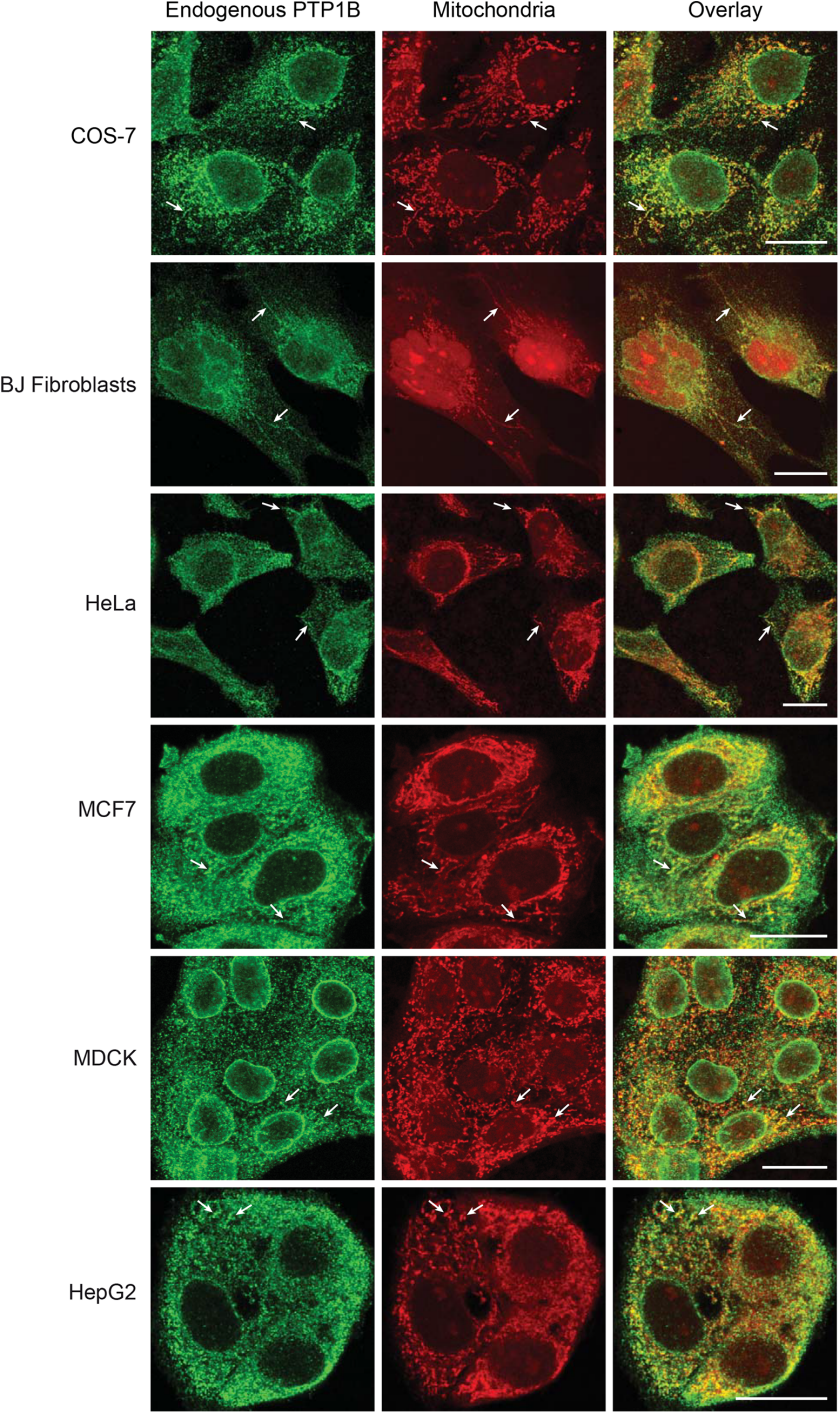
Mitochondrial localization of endogenous PTP1B in multiple mammalian cell lines. Mammalian cells were fixed with PFA and immunostained for endogenous PTP1B using an anti-PTP1B (Ab-1) mouse monoclonal primary antibody (Calbiochem) and a chicken anti-mouse secondary antibody conjugated with Alexa488 (Invitrogen). Mitochondria were stained with MitoTracker Red CMXRos (Invitrogen). COS-7, BJ Fibroblast, HeLa, MCF7, MDCK and HepG2 cells were visualized with confocal microscopy. Representative mitochondria are marked with arrows. Mitochondrial targeting of PTP1B was easier to assess in the cells with a flatter morphology towards the top of the figure as compared with the more compact cells towards the bottom. Scale bars: 20 μm.

Recent evidence from Western blots and electron microscopy has demonstrated the localization of PTP1B in mitochondria that were extracted from rat brain tissue [32,33]. To test whether this mitochondrial localization is of a more general nature, we probed multiple mammalian cell lines (COS-7, BJ Fibroblasts, HeLa, MCF7, MDCK and HepG2) with a mitochondrial marker (MitoTracker Red CMXRos) and a primary antibody specific for endogenous PTP1B (Fig 1). For the COS-7, BJ Fibroblast and HeLa cells, PTP1B accumulation at mitochondrial structures was clearly apparent in many cells, especially those having a flatter morphology with several isolated mitochondria at the cellular periphery. For MCF7, MDCK and HepG2, it became increasingly more difficult to find mitochondria that could be cleanly separated from the more general ER staining of PTP1B. However, close examination revealed significant colocalization of PTP1B with mitochondrial structures. The challenges that we encountered in discriminating mitochondria-specific subpopulations of PTP1B from its more general ER staining in particular cell lines offers a possible explanation for the fact that its mitochondrial localization was not discovered in earlier fluorescence-based studies [10,12].

PTP1B’s clear accumulation in the vicinity of the mitochondria might be explained as merely the accumulation of ER-resident PTP1B at regions of the ER in close apposition to the mitochondria, so-called mitochondria-associated membrane (MAM) sites [45–47]. We attempted to clarify this by preparing COS-7 cells as above but additionally expressing the ER marker mTagBFP-Sec61 (S1 Fig). A zoomed-in view of such cells shows the accumulation of PTP1B to isolated mitochondria and mitochondrial subregions not in apparent immediate proximity to the general ER. However, it is difficult to rule out the possibility of a subregion of the ER extending out to these otherwise isolated mitochondria. Additionally, the resolution limit of confocal microscopy of course prevents distinguishing the ER from the mitochondria at ER-mitochondria junctions. Two further aspects of the antibody staining shown in S1 Fig should be noted that reveal other disadvantages. First, the PTP1B antibody appears to stain only the periphery of many of the mitochondria in the image (including the mitochondrion indicated with the arrow). We have used only mild permeabilization conditions for all of the fixations reported in this study (0.1% Triton X-100 for 5 min). Such mild permeabilization is unlikely to permit access of the antibody to the mitochondrial interior, indicating that the staining we observe in the vicinity of the mitochondria is most likely associated with PTP1B proteins accessible from the cytosol. Unfortunately, harsher permeabilization conditions (more detergent and/or longer detergent incubation) adversely affected the staining (specific signal was much lower or altogether absent) preventing further resolution of this issue with antibody staining. Second, close examination of the zoomed-in images of S1 Fig reveals a highly “speckled” staining of PTP1B (present even in mitochondria-free regions along the nuclear ER), in contrast to the more smoothly distributed ER marker. Such “speckled” staining is typical of antibody-stained images due to incomplete staining of all target proteins and to the random and non-stoichiometric association of the secondary antibody to the primary antibody. The likely artifactual nature of this “speckling” is further addressed in the next section (see the below discussion of S3 Fig).

### PTP1B localizes to the outer mitochondrial membrane via its tail anchor

**Fig 2:**
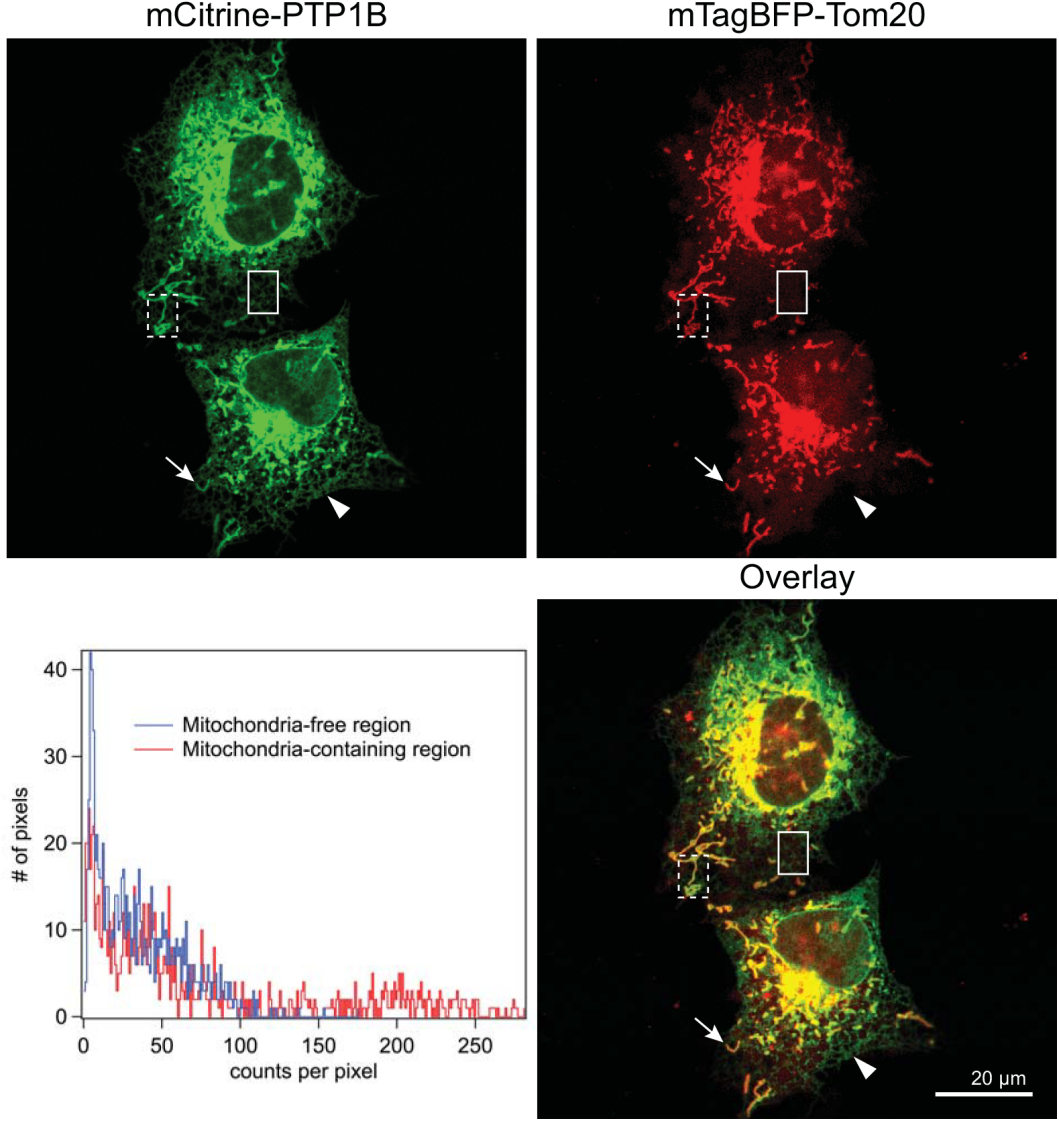
PTP1B localizes to the mitochondria in COS-7 cells. Confocal images of COS-7 cells expressing mCitrine-PTP1B along with the mitochondrial marker Tom20-mTagBFP. The mCitrine-PTP1B chimera localized to the general ER (arrowhead) and at a higher local concentration to the mitochondria (arrow). Scale bar: 20 μm.

Upon its expression in COS-7 cells, a chimera of mCitrine with the second splice variant of PTP1B (ending in FLFNSNT and the central focus of our current study) exhibited the same strikingly high concentration to structures coincident with the mitochondria (Fig 2, arrow indicates an individual mitochondrion) as compared to its general distribution along the ER (Fig 2, arrowhead indicates a mitochondria-free region of the ER). Its higher local concentration to mitochondrial structures was quantitatively verified by plotting the mCitrine-PTP1B brightness distribution (Fig 2 histograms) in a peripheral region free from mitochondria (blue histogram corresponding to the solid box in the images) and in a peripheral region containing mitochondria (red histogram corresponding to the dashed box in the images). The brightness distribution in the mitochondria-free region truncates at roughly 100 counts/pixel, whereas the distribution in the mitochondria-containing region extends to roughly 250 counts/pixel.

Which domain of PTP1B is responsible for its apparent mitochondrial partitioning? An obvious candidate is PTP1B’s ≤35 amino acid C-terminal tail anchoring domain [10,12]. Colocalization of the full-length chimera with a tail-anchor-only chimera (mCherry-PTP1Btail) yielded perfect overlap (panel A in S2 Fig), demonstrating the complete dependence of PTP1B’s subcellular partitioning on its tail anchor. Both constructs were again heavily concentrated at the mitochondria (labeled with Tom20-mTagBFP) as compared to their distribution along the ER, which was observed for the full-length and tail-only chimeras as a faint reticular network distinct from the mitochondrial marker. In a prior study, the subcellular distributions of the second splice variant (PTP1Btail) and the first variant (PTP1Btail^VCFH^) were shown to be identical [8]. We confirm and extend this previous observation, with our high-resolution images showing that both variants partition in exactly the same way to the ER and mitochondria (panel B in S2 Fig).

Further dynamic verification of the colocalization of full-length PTP1B with its tail anchor is shown in S1 Movie for live COS-7 cells. Pulsed-interleaved excitation (PIE, see Materials and Methods) was used to acquire essentially simultaneous (down to 25 ns) dual-color images of COS-7 cells expressing mTurquoise-PTP1B along with mCherry-PTP1Btail. Both constructs exhibited a perfect (and unchanging) overlap over the entire duration of the movie (6 minutes).

To more closely examine the exact distribution of PTP1B along the ER (as determined by its tail anchor), we have coexpressed the tail anchor-containing construct mCherry-PTP1Btail together with mTFP1-ER, which highlights the ER lumen (S3 Fig). In these images a clearly reticular expression of the tail anchor is observed that smoothly tracks the ER marker with no apparent clumping as was observed using antibody staining (S1 Fig). Residual differences between the two images in S3 Fig are likely attributable to their live-cell nature (taken 1 minute apart), optical/refraction differences between the two fluorescence channels or the difference between tracking the ER membrane (PTP1B tail anchor) versus the ER lumen (mTFP1-ER). Since the image in S3 Fig is of an overexpressed PTP1B isoform, it remains possible that the lower expressed *endogenous* PTP1B could be more clumped; further resolution of this issue is however beyond the scope of this manuscript. In S3 Fig, we used mTFP1-ER as the ER marker due to its reliable tracking of the *entire* ER due to its residence in the lumen; ER-membrane-bound Sec61, as was used in S1 Fig, is not guaranteed to track the entire ER membrane. For S1 Fig, Sec61 was a better tracker of the ER membrane as aldehyde fixation induces severe morphological changes in the ER, which were observed as a “blob-like” distribution of mTFP1-ER indicating regions of the ER with large amounts of lumen.

**Fig 3:**
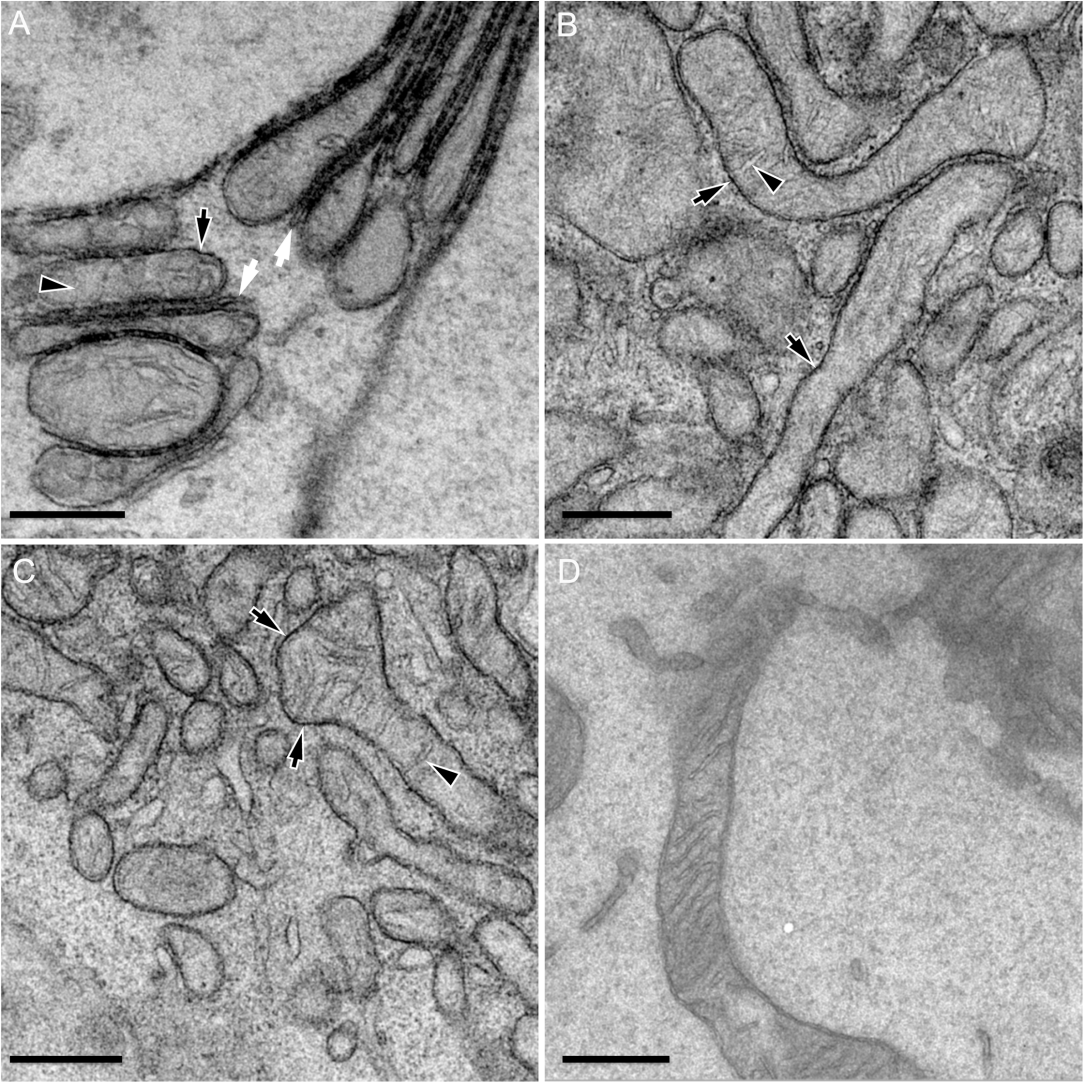
EM imaging of subcellular PTP1B partitioning. (A) High magnification EM image of several mitochondria expressing mTurquoise-APEX-PTP1B in COS-7 cells. The pronounced DAB precipitate was locally concentrated at the OMM (black arrow) and was notably absent from the intracristal spaces and matrix. Strong staining is also observed along ER subregions in direct apposition to mitochondria and therefore consistent with ER MAM sites (white arrows). (B) Similarly strong DAB precipitation was observed at the OMM of a COS-7 cell expressing mTFP1-APEX-PTP1Btail (black arrow). (C) DAB precipitation was observed at the OMM of a COS-7 cell expressing Tom20-APEX-mTurquoise (black arrow). (D) Image of unlabeled mitochondria from an untransfected COS-7 cell serves as a negative control. The black arrowhead indicates the absence of the labeling of the mitochondrial interior. Scale bars: 500 nm.

To better characterize the precise submitochondrial localization of PTP1B, we employed electron microscopy of specific DAB precipitation generated by the ascorbate peroxidase APEX [48] (Fig 3, S4 Fig, see Materials and Methods). Electron microscopy of a full-length chimera (mTurquoise-APEX-PTP1B, Fig 3A) or a tail-anchor-only chimera (mTFP1-APEX-PTP1Btail, Fig 3B) revealed their localization to the outer mitochondrial membrane (OMM) in COS-7 cells. Control images of cells expressing an OMM label (TOM20-APEX-mTurquoise, Fig 3C) showed a similar contrast at the OMM, with other untransfected control cells exhibiting only the lower generic contrast characteristic of lipids (Fig 3D). While the localization of PTP1B to the mitochondria is in agreement with the prior detection of the VCFH variant of PTP1B in mitochondria extracted from rat brain cells, both prior studies further observed PTP1B in the mitochondrial interior based either on biochemical assessment of submitochondrial fractions [32] or electron microscopy of immunogold staining [33]. We see no evidence for this internal pool of PTP1B. Our electron microscopy of the tail-anchor-only chimera (Fig 3B) importantly demonstrates that PTP1B’s OMM localization is completely dependent on its tail anchor. We note that expression of the full-length PTP1B led to the formation of tight aggregates of mitochondria and ER (Fig 3A, S4 Fig) with particularly strong staining at putative MAM sites along the ER. Self-aggregation of the ER was also observed. As the latter effect was previously observed upon overexpression of an ER-resident construct that weakly dimerized [49], the organellar aggregation that we observe is also likely due to a similar dimerization of the mTurquoise-APEX-PTP1B construct. Dimerization of APEX is unlikely, as no such aggregates were observed upon expression of the mTFP1-APEX-PTP1Btail construct. However, dimerization of either mTurquoise or of the full-length PTP1B (either directly through PTP1B-PTP1B dimerization or indirectly through recruitment of two different PTP1B proteins to the same substrate) is possible and is the most likely explanation of the tight intra- and inter-organellar associations that we observe.

### Heterologous expression of PTP1B’s tail anchor in yeast reveals a potential minor role of the GET/TRC40 pathway in ER insertion

**Fig 4:**
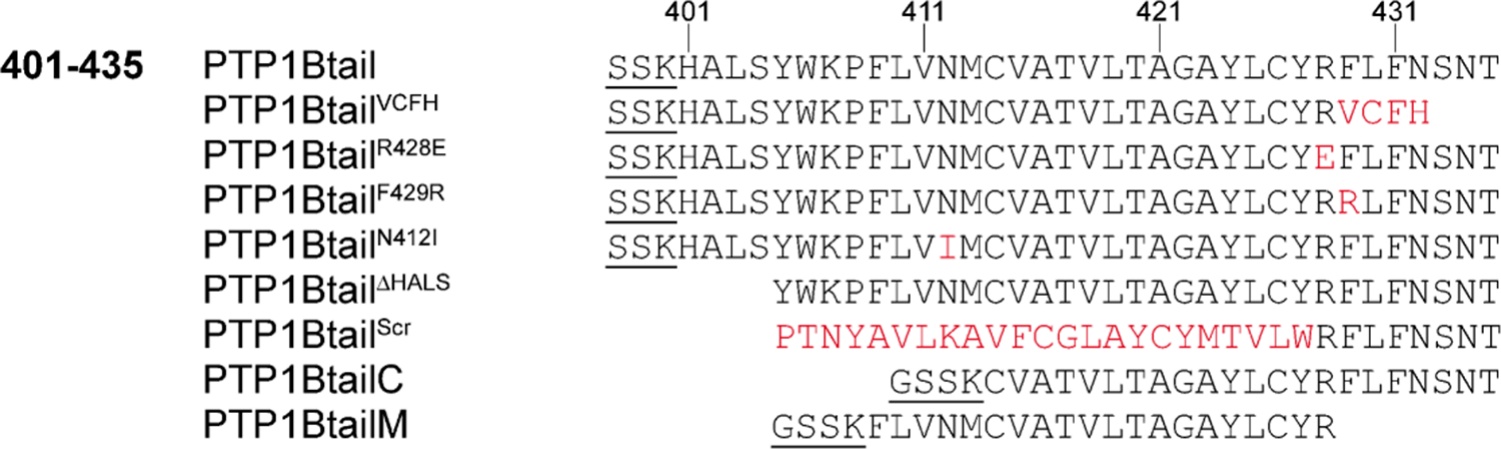
Wild-type and mutant PTP1B tail anchor isoforms examined in this study. See text for further description.

The above results reveal the dependence of PTP1B’s subcellular partitioning to the ER and to the OMM on its C-terminal hydropathic tail anchor (TA). In Fig 4, we give the sequences of the wild-type and mutant isoforms of PTP1B’s tail anchor that we examine in this study: wild-type isoform (PTP1Btail), wild-type splice variant (PTP1Btail^VCFH^), charge-altered isoforms (PTP1Btail^R428E^, PTP1Btail^F429R^), hydropathy-altered isoform (PTP1Btail^N412I^), N-terminally truncated isoform (PTP1Btail^ΔHALS^), scrambled isoform (PTP1Btail^Scr^) and further truncated isoforms (PTP1BtailC and PTP1BtailM). Residues that link these tail isoforms to the N-terminal fluorophore sequence are underlined (when present).

To isolate which pathway(s) might be responsible for targeting PTP1B to the ER and mitochondria, we employed the genetically pliable yeast *S*. *cerevisiae* to study the effect of mutations in largely conserved targeting pathways on PTP1B’s subcellular partitioning. In yeast, a “hydropathic code” appears to be at work [50], with clear differences in overall hydrophobicity of the tail anchors of proteins targeted to the various organelles (S5 Fig). The Kyte-Doolittle hydropathies [51] of a relatively complete set of the tail anchor-containing proteins in yeast [50,52], along with the class of GPI-anchored proteins like Gas1 [53] that contain a putative TMD at their C-terminus before cleavage and attachment of the GPI anchor, are displayed in S5 Fig. Organellar localizations are based on observations of the full-length proteins. The pattern already revealed by this representation is striking, with the clear difference in hydropathy of the mitochondrial versus ER proteins of particular significance for the present study. Based on hydropathy alone, we predicted that the tail anchor of PTP1B (its hydropathy profile is shown in black in S5 Fig) would preferentially localize to the mitochondria or to the nuclear envelope. However, a tail-anchor-containing chimera (yemCitrine-PTP1Btail) localized only to the ER and the vacuolar membranes upon its heterologous expression in yeast (S6 Fig), with no significant concentration at the mitochondria. This result was similar to previous observations of the mammalian protein Bcl2, which has a tail anchor with similar hydropathy (S5 Fig, last panel) that localizes it to the ER/mitochondria in mammalian cells [54,55] but only to the ER in yeast [56].

What could account for this discrepancy in targeting? One of the principal differences between yeast membranes and higher eukaryotic membranes is the production and incorporation of ergosterol versus cholesterol. Membranes that incorporate ergosterol are more ordered and therefore thicker and less fluid than those that incorporate the same concentration of cholesterol [57] and could therefore account for this difference in stability of the tail anchor’s organelle-specific insertion in yeast versus mammalian cells. To test this, we used a recently reported yeast strain in which intracellular ergosterol is replaced by cholesterol through exchange of the two terminal enzymes in the ergosterol production pathway with their cholesterol-specific counterparts [58]. Localization of the tail anchor of PTP1B in this mutant strain (cholesterol) was identical to that in its wild-type (ergosterol) counterpart (S7 Fig), indicating no influence on the presence of specific sterols on the subcellular targeting of the tail anchor of PTP1B. While we found no difference of the localization of the tail anchor in yeast strains that synthesized ergosterol versus cholesterol, differences in the *overall* sterol concentration (or its specific concentration within rafts) might still affect global (or local) membrane thickness and fluidity of specific organelles in mammalian cells versus yeast [59]. In fact, previous in vitro studies have demonstrated that membranes with high sterol content inhibit the spontaneous insertion of the tail anchor proteins CytB5 [60] and PTP1B [13], supporting this hypothesis.

While we could not immediately probe the targeting of the tail anchor of PTP1B to the OMM in yeast (due to its absence there), we note that OMM insertion could be spontaneous (as has been reported for multiple TA proteins [61,62]) or through complete or partial assistance of the translocase of the outer membrane (TOM complex [63,64]).

**Fig 5:**
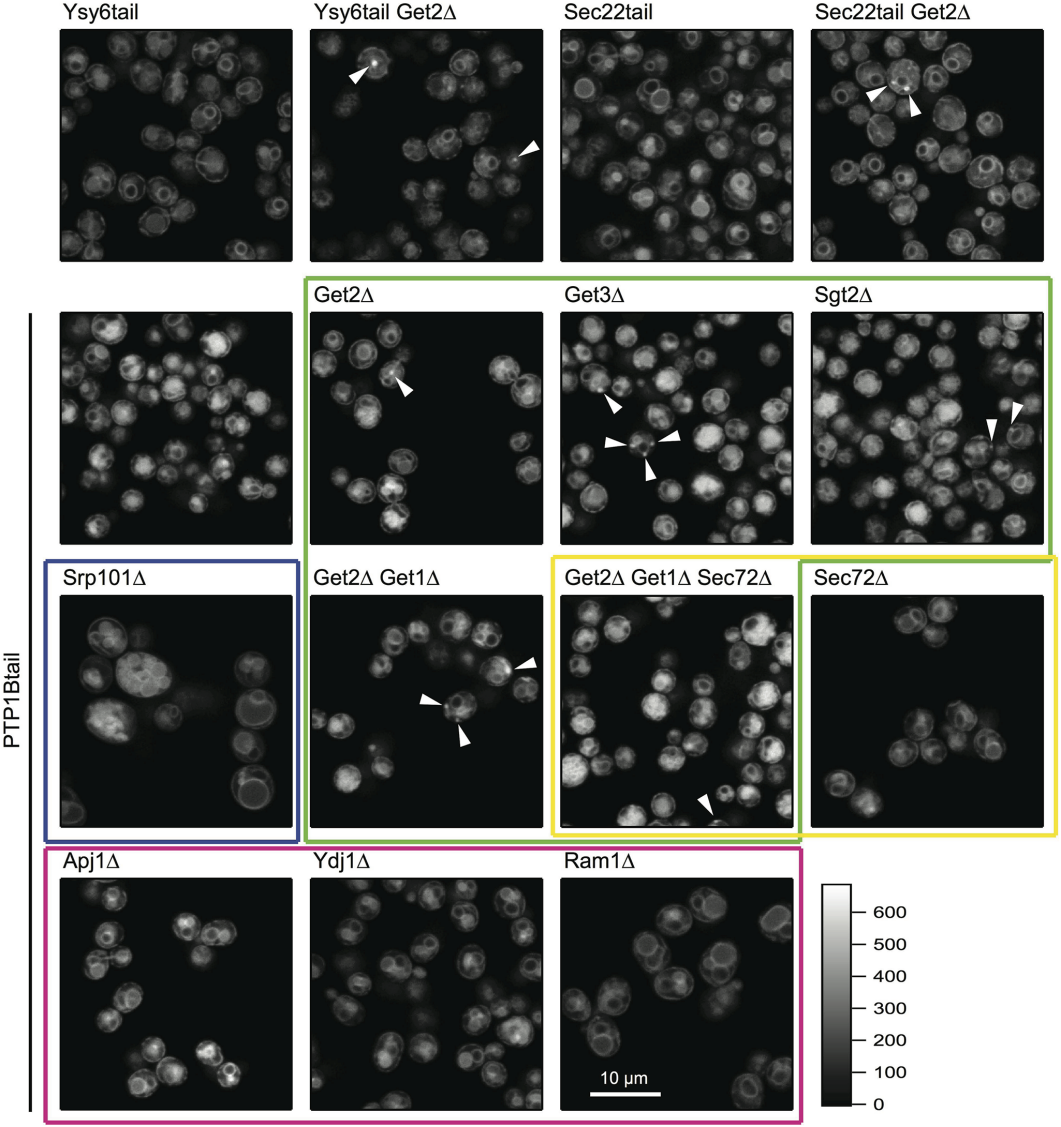
Systematic study of the potential pathways responsible for the subcellular targeting of the tail anchor of PTP1B in yeast. We used an aggregate-formation assay to examine the impact of deletion of the various insertion pathway components in yeast. Aggregate formation in strains expressing tail anchors from Ysy6 and Sec22 upon deletion of the GET pathway component Get2 was used as a control. Comparing wild-type with Get2∆ deletion strains that expressed yemCitrine-Ysy6tail or yemCitrine-Sec22tail, we observed visible aggregates in a fraction of the Get2∆ cells that were consistent with previous findings (though the bulk of these proteins still managed to insert properly). We observed similar aggregates in cells expressing yemCitrine-PTP1Btail. Slightly more aggregates were observed in Get3∆ cells and significantly fewer in the Sgt2∆ cells of this strain. A strain in which both Get2 and Get1 were deleted was similar to the Get2∆ deletion strain. The green box indicates all strains in which one or more GET pathway components were deleted. Deletion of the α subunit of the SRP receptor (Srp101) led to a much larger cell phenotype (blue box), but no change in the ER and vacuolar partitioning of the PTP1B tail anchor. Deletion of Sec72 (yellow box), an essential factor of the Sec62/63 pathway, also did not alter the localization of the PTP1B tail anchor. Deletion of Sec72 in the Get2∆/Get1∆ strain did not further alter the localization of the PTP1B tail anchor beyond that observed in the Get2∆/Get1∆ strain. Finally, deletion of the chaperones Apj1 and Ydj1 (as well as the farnesyl transferase of Ydj1, Ram1) did not alter PTP1B’s subcellular localization (red box). The intensity scale shown at the bottom right and indicating photon counts/pixel is applicable to all images, as is the scale bar shown in the bottom right panel. Scale bar: 10 μm.

To assess which specific mechanism(s) might be responsible for insertion of the tail anchor into the yeast ER, we examined mutant yeast strains that were deleted in important proteins associated with the various distinct tail anchor pathways for ER insertion (Fig 5).

A major mechanism for tail insertion is through the GET/TRC40 pathway [16–20,22,23]. To examine its role in the insertion of the tail anchor of PTP1B, we systematically deleted proteins involved in this pathway in a yeast strain chromosomally expressing yemCitrine-PTP1Btail. Deletions of ER-resident Get2 (functional mammalian homologue CAML [65]), cytosolic chaperone Get3 (mammalian homologue TRC40 [17]), the chaperone-interacting protein Sgt2 (mammalian homologue SGTA [66]) and/or the ER-resident Get1 (mammalian homologue WRB [67]) led to the formation of cytosolic aggregates (arrows in Fig 5), indicating potential involvement of the GET pathway. Similar aggregates were observed under the same experimental conditions for the tail anchors of Ysy6 and Sec22 (Fig 5), which were previously identified as GET pathway targets using the same phenotypic assay [18,20]. Note that there is no increase in the level of cytosolic aggregates in the Get2∆/Get1∆ strain over that observed in the Get2∆ strain, in line with the observation that Get1 expression is already significantly reduced in the absence of its binding partner Get2 [68]. We caution that, despite this clear phenotype of aggregate formation, the GET pathway may still not be *directly* responsible for insertion of any of these tail anchors. Aggregate formation in these mutants could be nucleated by a distinct set of proteins dependent on the GET pathway; once such aggregates are formed, they could generically recruit other tail anchor proteins (including possibly PTP1B, Ysy6 or Sec22) that are not directly dependent on the GET pathway for their insertion (e.g. normally spontaneously inserted). It is important to note that in all of these GET pathway mutants, the majority of the tail anchors of PTP1B, Ysy6 and Sec22 still manages to reach the ER, implying an at most minor role for the GET pathway in their ER insertion.

Evidence for a role of the SRP pathway [24] in the post-translational targeting to the ER of some tail anchor proteins like the β subunit of Sec61 and synaptobrevin has been previously reported [69]. To abrogate the SRP pathway, we deleted Srp101, the essential α subunit of the SRP receptor. Deletion of Srp101 results in a viable strain [70] but one with six-fold slower growth and a roughly three-fold increase in cell size (Fig 5). In such a mutant, the tail anchor still localized entirely to endomembranes consistent with the ER and vacuole.

To investigate the contribution of the Sec62/63 pathway [25,26,71], we deleted the essential component Sec72 of this pathway in yeast. The tail anchor of PTP1B inserted normally in this strain, with the phenotype of a strain triply deleted for Get2/Get1/Sec72 identical to that of the double deletion Get2/Get1 (Fig 5). These results were in line with similar observations of heterologously expressed mammalian Cytochrome B5 (CytB5, hydropathy profile shown in the last panel of S5 Fig) in yeast, which also showed no dependence on the Sec62/63 pathway for its insertion (through use of temperature-sensitive mutants of the Sec62/63 pathway [72]). Our results are also in line with other recent experiments demonstrating no significant role for Sec62/63 in the insertion of specific tail anchored proteins [25].

Chaperones [14,15] may also assist in the insertion of PTP1B into the ER. A possible role for general chaperones in the insertion of the similarly tail anchored CytB5 has previously been hypothesized based on oxidation studies [73]. Along the same lines, deletions of the chaperones Apj1 (DnaJ-family) and Ydj1 (Hsp40), as well as the farnesyl transferase Ram1 of Ydj1 were previously reported to affect insertion of GPI-family mutants through the observation of visible aggregates in many cells [53]. These GPI-family proteins contain C-terminal regions with similar hydropathy to the PTP1B tail anchor (S5 Fig), making their particular chaperones interesting targets for assessing their effect on PTP1B’s membrane insertion. Indeed, a possible role of Hsp40/Hsp70 chaperone-assisted insertion of PTP1B into the ER of mammalian cells has previously been reported [14]. For the PTP1B tail, however, deletion of Apj1, Ydj1 (Hsp40) and Ram1 did not at all affect its localization to the ER and vacuolar membranes (Fig 5).

To conclude, insertion of the tail anchor of PTP1B into the yeast ER appears not to be mediated by the SRP pathway or the Sec62/63 pathways and is assisted in at most a minor way by the GET pathway. Deletion of interesting candidate chaperones also did not impede ER insertion. Our partial or complete ruling out of many of these pathways provides further circumstantial evidence for the possible importance of spontaneous insertion into the ER (as previously shown in vitro for PTP1B [13]).

### Subcellular partitioning of PTP1B’s tail anchor is highly sensitive to its exact composition

In this section, we examine the affect of changes in the tail anchor’s composition—specifically, its TMD length, C-terminal charge and hydropathy—on its subcellular partitioning in mammalian and yeast cells.

The length of the hydrophobic TMD of a tail anchor protein can determine its membrane specificity [74,75]. To explore the role of TMD length on the subcellular targeting of PTP1B, we examined truncations of the tail from either end. A truncation of the N-terminal sequence (HALS) exhibited an identical partitioning to the mitochondria/ER as for the full-length tail (Fig 4, see also panel A of S11 Fig), proving that the 31 amino acids at the C-terminus of PTP1B are already sufficient to account for its localization.

Further truncation of the N-terminus, resulting in the PTP1BtailC construct of only 22 amino acids (Fig 4), was previously shown to be sufficient for its ER targeting [12]. We found, however, that the PTP1BtailC construct localized it not only to the ER, but also to the Golgi and cytosol in COS-7 cells, with no presence at the mitochondria (panels A and B of S8 Fig). The PTP1BtailC chimera was also found at rapidly-moving vesicles (punctate structures in panels A and B of S8 Fig). Unexpectedly, in yeast the PTP1BtailC chimera localized to the ER *and* the mitochondria, with no localization at the Golgi (panels C–E of S8 Fig).

Another tail anchor mutant spanning the “middle” putative-TMD-containing region of the wild-type tail anchor (PTP1BtailM, Fig 4) was also previously shown to be sufficient for its ER targeting [12]. As for the PTP1BtailC anchor above (panels A and B of S8 Fig), we found that the PTP1BtailM anchor localized not only to the ER, but also to the Golgi and cytosol in COS-7 cells, with again no presence at the mitochondria (panels F and G of S8 Fig). Unlike the PTP1BtailC isoform, though, no further localization to rapidly moving vesicles was observed. In yeast, the PTP1BtailM chimera localized identically to the PTP1BtailC isoform to the ER and the mitochondria but not the Golgi (panels H–J of S8 Fig).

In COS-7 cells and in yeast cells, both the PTP1BtailC and PTP1BtailM isoforms are equally able to reach the ER; however, the additional difference in localization to the Golgi (COS-7) or to the mitochondria (yeast) is puzzling. In the case of spontaneous insertion, this might be explained by differences in lipid composition of both the Golgi and the mitochondria in COS-7 versus yeast. Such organellar differences in lipid composition could affect the fluidity of the membrane as well as the width of the lipid bilayer [59] (determined by the properties of the acyl chains, in particular their lengths), both of which could alter the retention of these tail anchor isoforms.

To summarize our findings on altered TMD length, truncations of the 35 amino acid tail anchor from its N- and/or C-termini, while preserving the ability to insert into endomembranes [12], nevertheless generated dramatic differences in *specific* subcellular partitioning that intriguingly differed for yeast versus higher eukaryotes.

The C-terminal charge of tail anchors has also been shown in the past to affect the targeting of tail anchor proteins (see the references below). For PTP1B, the arginine at position 428 out of 435 contributes a single positive charge at the C terminus. To examine the effect of the presence of this charge on the targeting of PTP1B, we expressed mutants for which this positive charge is replaced with a negative charge (PTP1Btail^R428E^, Fig 4) or augmented by an additional neighboring positive charge (PTP1Btail^F429R^, Fig 4). Conversion to a negative charge led to complete restriction of the tail anchor to the ER with no detectable mitochondrial presence in COS-7 cells (panel A of S9 Fig); in yeast, the negative tail anchor localized largely to the ER and only somewhat to the vacuolar membrane (panel B of S9 Fig) in contrast to the more equal ER/vacuolar distribution of the wild-type tail (S6 Fig). Augmentation of the C-terminal positive charge led to complete targeting of the tail anchor to the mitochondria in COS-7 cells (panel C of S9 Fig); in yeast, this tail anchor was largely redirected to the mitochondria but also still somewhat present on the ER and vacuolar membranes (panel D of S9 Fig).

Our results on C-terminal tail anchor charge are consistent with previous results on the tail anchor of CytB5. For CytB5, the wild-type tail has a net charge of −1, which localizes it exclusively to the ER [76]. A CytB5 isoform that is truncated at its C terminus, generating a +1 charged tail, localizes as well to the mitochondria [77]. Exclusive mitochondrial localization is accomplished through addition of another positive charge to this isoform, generating a +2 net charge [78]. Other tail anchor proteins for which C-terminal positive charge plays a significant role in their mitochondrial targeting include synaptobrevin/VAMP-1B [79] and OMP25 [75] in mammalian cells and Fis1 [62] in yeast. This positive-charge-based relocation (and retention) to the mitochondria could be due to the presence of higher net negative charge in these compartments (perhaps due to the presence of the negatively charged mitochondrial-specific lipid cardiolipin [80,81]). Too little positive charge in the wild-type tail anchor of PTP1B (as compared with the more positively charged mutant) prevents it from accessing the mitochondria, which may indicate that yeast mitochondrial membranes have a lower negative charge than mammalian mitochondria.

Finally, as discussed already above, tail anchor hydropathy is also an important determinant of membrane specificity. Based on the yeast “hydropathic code” (S5 Fig), which shows a clear preference of tail anchors with low hydropathy for the mitochondria and with high hydropathy for the ER, we hypothesized that an increase in the hydropathy of the wild-type tail anchor in mammalian cells might be sufficient to shift it away from the mitochondria to the ER. Indeed, a tail anchor with significantly higher hydropathy (PTP1Btail^N412I^, Fig 4) localized exclusively to the ER with no detectable presence at the mitochondria in COS-7 cells (S10 Fig). These results are similar in nature to results obtained for hydropathic mutants of the mitochondrial-localizing yeast tail anchor protein Fis1 [82]. As the wild-type tail anchor of PTP1B is already restricted in its localization to the ER (and vacuolar membrane) in yeast, we expected that this mutation would have no effect; indeed, this mutant tail anchor localized identically to the wild-type isoform in yeast (data not shown).

**Fig 6:**
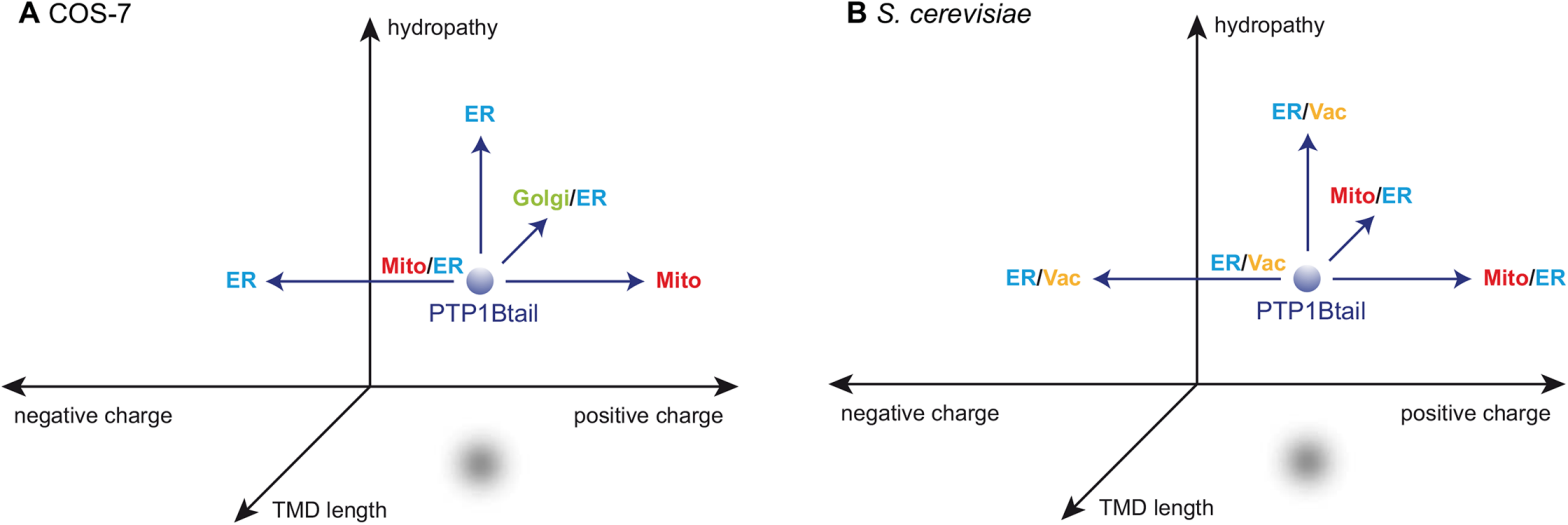
Tail anchor targeting is largely dictated by three different factors: TMD length, C-terminal charge and hydropathy. Our experimental localization results for different tail isoforms in COS-7 cells and yeast cells are summarized. We have shown that the wild-type localization of PTP1Btail to the mitochondria and ER (Mito/ER) is highly sensitive to changes in these three factors (see text and S8 Fig, S9 Fig and S10 Fig).

In the above, we have shown that the subcellular targeting of PTP1B depends sensitively on three generic features of its tail anchor—namely, its TMD length, charge and hydropathy. Our overall findings regarding the localization(s) of the various isoforms that we tested in both mammalian and yeast cells are summarized in Fig 6 and S1 Table. The most significant result is the clear dependence on charge of ER versus mitochondrial partitioning in both mammalian and yeast cells, with negative C-terminal charge leading to complete retention to the ER and (increasingly) positive charge shifting the balance to the mitochondria. That this charge dependence is similar in yeast importantly suggests evolutionarily shared mechanisms for delivery of PTP1B (or closely related isoforms) to the mitochondria. No *mammalian-specific* chaperones are therefore required to explain the mitochondrial localization of the wild-type isoform in mammalian cells but not yeast. Instead, slight differences in the charged lipid composition of these organelles in mammalian versus yeast cells provides the most compelling explanation. In addition to charge, it is clear that tail anchor hydropathy is another important factor that specifically impacts the ER-mitochondrial balance. Mutation of a single amino acid, leading to an increased hydropathy, was sufficient to restrict PTP1B’s tail anchor to the ER in mammalian cells, suggesting that the ER membrane presents a better environment for tail anchors with high hydropathy than the mitochondrial membrane (in line with the “hydropathic code”, S5 Fig). Both charge and hydropathy are therefore independently capable of affecting the ER-to-mitochondrial balance. Finally, the TMD length of the tail anchor introduces yet another important variable; however, the more complex results from these studies (which differ in mammalian cells versus yeast) do not provide as straightforward an interpretation as changes in charge and hydropathy.

To test whether the three features outlined in the above are truly *sufficient* to determine membrane specificity, we constructed a mutant version of the tail anchor for which the TMD was completely scrambled in its amino acid sequence in a way that closely preserved its exact hydropathic profile (by exchanging each original amino acid with a similarly hydrophobic amino acid, Fig 4); TMD length and C-terminal charge were of course also preserved by this scrambling. Efficient cloning of this scrambled isoform using long primers required truncating the original tail anchor by four amino acids. We therefore first tested the effect of this mild truncation on subcellular partitioning. As reported above, the terminal 31 amino acids of the tail (PTP1Btail^ΔHALS^, Fig 4) are already sufficient to account for the “wild-type” targeting of PTP1B to the mitochondria and ER (panel A of S11 Fig). A scrambled tail isoform (PTP1Btail^Scr^, Fig 4) was then constructed that has, by design, a very similar hydropathy profile (panel A of S11 Fig). The scrambled tail localized to the ER and the Golgi, as well as to rapidly moving punctate vesicles, with no discernible presence at the mitochondria (panels B and C of S11 Fig). This striking difference in localization from the original PTP1Btail^ΔHALS^ isoform (which localizes identically to the wild-type anchor) suggests that—in addition to its TMD length, charge and hydropathy (Fig 6)—the *exact* amino acid sequence of the tail also appears to contribute to its subcellular targeting, adding an additional layer of complexity to the tail anchor-mediated subcellular partitioning of PTP1B.

### FLIM reveals an interaction of PTP1B with EGFR at the outer mitochondrial membrane

Tyrosine phosphorylation of multiple proteins in the mitochondria appears to be a significant regulatory mechanism for general mitochondrial functions [34]. The presence of PTP1B at the mitochondria could be important for the local regulation of these phosphotyrosine-containing targets. To examine the possible presence of tyrosine phosphorylation at the mitochondria, we employed two commonly used probes that recognize phosphotyrosines, one containing a double Src homology 2 domain (dSH2-YFP [83]) and the other containing the phosphotyrosine-binding domain of Shc (PTB-mCherry [84]).

As mentioned above, previous studies have revealed a significant mitochondrial population of the non-receptor tyrosine kinase c-Src as well as other members of the Src family [33,36–40]. To probe Src family activity at the mitochondria, we examined the localization of the dSH2-YFP probe in COS-7 cells. We observed no significant mitochondrial localization of this probe either before or after EGF stimulation (S12 Fig). However, it is likely that this probe can only access the cytosolic face of the OMM, implying at any rate no significant active c-Src (or other family members?) at this particular submitochondrial region in COS-7 cells.

Upon expression in COS-7 cells, PTB-mCherry displayed a basal recruitment to the mitochondria that significantly increased upon EGF stimulation (S12 Fig). The PTB-mCherry probe may be detecting important Shc substrates like the receptor tyrosine kinases ErbB1 and ErbB2, for which mitochondrial pools have recently been claimed [41–43].

PTP1B has previously been shown to interact with active ErbB1 [27]. To test whether the OMM-resident pool of PTP1B participates in this interaction, we used confocal time-domain fluorescence lifetime imaging microscopy (FLIM) to visualize the direct interaction of donor-labeled ErbB1 (ErbB1-mCitrine) with an acceptor-labeled D181A trapping mutant [11] of PTP1B (mCherry-PTP1B^D/A^) by Förster Resonance Energy Transfer (FRET). COS-7 cells expressing these constructs were starved overnight and stimulated with EGF. Continuous recording of the donor fluorescence before and during the stimulation allowed dynamic monitoring of the ErbB1-PTP1B interaction across the cell, including at the mitochondria (S13 Fig, S2 Movie). In the top two rows of S13 Fig, control cells expressing only ErbB1-mCitrine are shown. These cells exhibited a stable and spatially uniform lifetime of 3.02 ns (the isolated spots of low lifetime are consistent with autofluorescent particles, which were also often observed in untransfected cells). ErbB1 was not observed to accumulate to the mitochondria (labeled with Tom20-mTagBFP) after EGF stimulation (see further discussion below). In the third and fourth rows of S13 Fig, COS-7 cells coexpressing donor-labeled ErbB1 and the acceptor-labeled trapping mutant (mCherry-PTP1B^D/A^) exhibited a low basal interaction before stimulation that was sharply increased upon the addition of EGF. The full lifetime movie of these cells (S2 Movie) was consistent with previous claims of the restricted interaction of PTP1B with ErbB1 only after receptor internalization by endocytosis [27]. A high level of FRET between ErbB1-mCitrine and mCherry-PTP1B^D/A^ was detectable on perinuclear structures consistent with the mitochondria (labeled with Tom20-mTagBFP). These results suggest an important role for PTP1B in the local dephosphorylation of ErbB1 at the mitochondria, both before and after EGF stimulation.

As an important control, we also examined cells coexpressing ErbB1-mCitrine with an acceptor-labeled chimera containing only the tail anchor of PTP1B, mCherry-PTP1Btail (S14 Fig). In these cells, no basal interaction was detected and no significant increase in interaction was observed following EGF stimulation. This importantly shows that overexpression of the acceptor-labeled tail is insufficient to affect the lifetime of ErbB1-mCitrine on the ER or the mitochondria. The decreased lifetime in S13 Fig for the acceptor-labeled trapping mutant is therefore indicative of a direct interaction of ErbB1-mCitrine with mCherry-PTP1B^D/A^.

To better distinguish the interaction of ErbB1 with PTP1B^D/A^ across the cell, we also performed similar experiments with different chimeras that localized the catalytic domain of the trapping mutant exclusively to the ER (C-terminal fusion of the tail anchor of the Sec61β subunit [14], mCherry- PTP1B^D/A^-ER, first and second rows of S15 Fig) or to the OMM (C-terminal fusion of the +2 positively charged mutant tail anchor of CytB5 that localizes exclusively to the OMM [78], mCherry-PTP1B^D/A^-OMM, third and fourth rows of S15 Fig). For both the ER- and OMM-resident chimeras, a low basal interaction before stimulation and a robust increase in interaction after stimulation were observed only at these respective compartments. These results importantly reveal that the interaction of PTP1B^D/A^ with ErbB1 involves direct interaction of these proteins at the ER or the cytosolic face of the OMM.

As mentioned above, the EGF receptor (both ErbB1 [42] and ErbB2 [43]) has previously been reported to localize within the mitochondria. To probe for active receptor in the mitochondrial interior, we examined the interaction of donor-labeled ErbB1 with acceptor-labeled PTP1B^D/A^ chimeras targeted either to the IMS (N-terminal fusion of Smac domain [85], mCherry-PTP1B^D/A^-IMS, first and second rows of S16 Fig) or to the matrix (N-terminal fusion of COX8A domain [86], mCherry-PTP1B^D/A^-MAT, third and fourth rows of S16 Fig). In neither case were we able to detect a decreased lifetime at the mitochondria. Moreover, as for the control cells shown in the first two rows of S13 Fig, no significant concentration of ErbB1 could be detected at the mitochondria either before or after EGF stimulation.

As previous studies reporting the localization of ErbB1 or ErbB2 to mitochondria were performed using cells that either naturally overexpressed c-Src (breast cancer cells [43]) or were cotransfected with a c-Src construct (10T1/2 cells [41,42]), we undertook a separate set of experiments in which COS-7 cells were transfected with ErbB1-mCitrine, c-Src-mTurquoise and Tom20-mTagBFP. In these experiments, we also observed no detectable mitochondrial presence of the fluorescently labeled c-Src or ErbB1 (data not shown). We also performed a set of experiments using MCF-7 cells, which naturally express high levels of c-Src, but again we were unable to see mitochondrial recruitment of fluorescent ErbB1 either before or after EGF treatment (in cells doubly transfected with ErbB1-mCitrine and Tom20-mTagBFP, data not shown). The discrepancy of our current experiments with previous reports of ErbB1 (and c-Src) at the mitochondria could be due to a number of factors that are beyond the scope of the current study (cell line specificity, an only small fraction of mitochondrial-localizing ErbB1/c-Src or more detailed experimental differences between our live cell studies of fluorophore-labeled proteins and previous studies of endogenous proteins in fixed cells or upon mitochondrial extraction [41,42]).

To summarize, our FLIM experiments collectively reveal a robust interaction of mCherry-PTP1B^D/A^ with ErbB1-mCitrine at the OMM, suggesting a possibly significant role for PTP1B in regulation of pools of ErbB1 localized either directly at, or simply in the vicinity of, the OMM.

## Discussion

Using confocal microscopy of endogenous/overexpressed PTP1B, we have shown that PTP1B localizes to the mitochondria in diverse mammalian cell lines (not only in rat brain cells [32,33]), with this mitochondrial localization of PTP1B determined solely by its tail anchor. From our EM studies, we show that PTP1B is targeted to the OMM by its tail anchor (corroborated by our FLIM studies). Significantly, we do not detect the FLFNSNT variant of PTP1B (the focus of our study) *within* the mitochondria, as observed for the VCFH variant of PTP1B in mitochondria extracted from rat brains [32,33]. This discrepancy could be due to different targeting of these variants or to cell type differences. The former hypothesis seems less likely, though, as both the VCFH and FLFNSNT variants partition absolutely identically to the mitochondria and ER (panel B of S2 Fig). Despite this high degree of similarity, it remains possible that the submitochondrial partitioning of these variants might still differ. Our studies in yeast demonstrate the possibility of an at most minor role for the GET pathway (TRC40 pathway in mammalian cells) in its ER insertion with the deletion of other pathways or chaperones not impeding its ER entry at all, providing further circumstantial evidence for the possible importance of spontaneous insertion. While our studies have collectively reduced the spectrum of possibilities, the *exact* mechanisms (whether spontaneous or protein-mediated) that ultimately control PTP1B’s tail-anchor-driven insertion into the ER and into the OMM will require further exploration. In addition to PTP1B, previous immunogold stainings of other important phosphotyrosine regulators (Lyn [36], c-Src [37], ErbB1 [42] and ErbB2 [43]) have also indicated their presence at and even *within* mitochondria. Based on our results on PTP1B, it is clear that similar complementary studies of the localization of these other key regulators are warranted to test these claims.

Targeted mutagenesis and heterologous expression in yeast were used to reveal intriguing and likely fundamental differences in the tail-anchor targeting of PTP1B to different organelles within a single host cell and for its targeting to the same organelle in widely separated hosts across the evolutionary tree. While the wild-type isoform did not localize to the mitochondria in yeast, introduction of a single additional C-terminal charge (or truncation) were sufficient to shift it to the mitochondria (Fig 6), providing valuable insight into the particular properties of PTP1B’s tail anchor that control its subcellular partitioning. Furthermore, based on our observations in yeast, the lack of mitochondrial targeting of the wild-type isoform is *not* due to the absence in yeast of a mammalian-specific protein-based mechanism but is rather more likely due to a slight difference in the lipid compositions of yeast versus mammalian mitochondria.

Similar systematic exploration of other tail anchors should help confirm the general importance of the three dimensions of TMD length, charge and hydropathy (see Fig 6) on subcellular partitioning. Tail anchor isoforms that reside in certain regions of this 3D phase space may act as “restricted keys” for accessing the lipid bilayer of only a single compartment (e.g. mitochondria alone), whereas tail anchors residing in other regions may act as “skeleton keys” permitting access to multiple compartmental membranes (e.g. ER/mitochondria or ER/Golgi). Design principles based on the tail anchor properties outlined above could be used to construct synthetic tails with high specificity for each organellar membrane in the cell [87,88]. Further examination of these aspects of tail anchor targeting, in addition to a more detailed portrait of the global and local concentrations of particular lipid isoforms (including charged isoforms and isoforms that affect bilayer width/fluidity), should help to account for the observed partitioning of general tail anchor proteins in both higher and lower eukaryotes. While this simple 3D view of TMD length, charge and hydropathy presents a useful reduction of complexity, additional sequence-specific aspects should not be ignored, as we have demonstrated through the different localization of an isoform that has a scrambled amino acid sequence but that otherwise preserves these three properties (S11 Fig).

PTP1B’s tail anchor-dependent presence at the mitochondria likely reflects possibly important roles for it in the regulation of phosphotyrosine-based signaling there (e.g. regulation of mitochondrial EGFR [41–43], Src [33,36–40] and SHP2 [33,40,44], or modulation of the electron transport chain [34,35]). Further investigations of PTP1B’s interactions with known and putative substrates using standard biochemical approaches or advanced microscopy would be useful. PTP1B’s potential regulation of basic mitochondrial functioning could be revealed using assays for cellular oxygen consumption, electron transport chain activity, glucose uptake, lactate production or the ATP/ADP ratio [43]. Finally, methods for detecting different post-translationally modified isoforms of PTP1B (phosphorylated, oxidized, sumoylated, cleaved) would be useful to develop and employ for discerning any potential differences (that may have functional consequences) of mitochondrial PTP1B from the more distributed pool of PTP1B along the ER. Such deeper investigations of the role of the mitochondrial pool of PTP1B on the regulation of local tyrosine-based signalling and fundamental mitochondrial processes should help shed light on its apparently complex roles in both normal and diseased states [6,7].

## Materials and Methods

### Yeast plasmids

The constructs pAK51 (yemCitrine-PTP1Btail), pAK72 (yemCitrine-Ysy6tail) and pAK86 (yemCitrine-Sec22tail) that we used for construction of the respective strains sAK199, sAK238 and sAK264 (S2 Table) were derived from pYM-N17 (see Janke et al. [89]). Briefly, the resistance cassette containing the gene for resistance to ClonNAT was reversed between the SalI/SacI sites of pYM-N17 to generate pYM-N17-Natrev. Through a series of changes in the latter construct, we obtained pAK51, in which the GPD promoter is followed by CGGATTCTAGGCTAGCCGCCGCC (unique NheI site underlined), the sequence for yemCitrine without its stop codon (gift from the Knop lab), a short linker, the sequence for the C-terminal 35 amino acids of PTP1B (tail anchor), followed by a unique EcoRI site and then the Tcyc1 terminator (with its insertion removing the original EcoRI site from pYM-N17-Natrev). To obtain pAK72 (or pAK86), an NheI-containing 5′ primer of yemCitrine was used along with a long EcoRI-containing 3′ primer of yemCitrine containing the C-terminal 31 amino acids of Ysy6 (or C-terminal 31 amino acids of Sec22) was used to generate the PCR product NheI-yemCitrine-Ysy6-EcoRI (or NheI-yemCitrine-Sec22-EcoRI) for replacement of yemCitrine-PTP1Btail between the NheI and EcoRI sites of pAK51. Plasmids pAK87 (yemCitrine-PTP1BtailM) and pAK88 (yemCitrine-PTP1BtailC), which contained the amino acid sequences given in Fig 4, were constructed in a similar fashion. Plasmid pAK104 (yemCitrine-PTP1Btail^R428E^) was constructed by double point mutation of the codon for arginine in pAK51 (AGG→GAG) as well as pAK111 (yemCitrine-PTP1Btail^F429R^) by double point mutation of the indicated codon for phenylalanine (TTC→CGC). Plasmid pAK109 (yemCitrine-PTP1Btail^N412I^) was constructed by point mutation of the indicated codon for asparagine (AAC→ATC).

The expression plasmid pAK100 (p415-yemCitrine-PTP1Btail) was constructed by PCR of yemCitrine-PTP1Btail from pAK51 flanked by SalI and XhoI restriction sites for insertion into SalI/XhoI-cut p415-GPD-mCherry (gift from M. Knop).

### Mammalian plasmids

All mammalian plasmids listed below utilized pcDNA3.1(+/-) backbones (Clontech) with the indicated genes fused with standard genetically encoded fluorophores (see Walther et al. [90] for citations) and expressed using a cytomegalovirus (CMV) promoter.

The following plasmids used in this study were: pmTFP1-ER (Allele Biotechnology), mCitrine-PTP1B (described in Yudushkin et al. [28]), Tom20-mTagBFP and mTagBFP-Sec61β (gifts of R. Stricker and E. Zamir), GalNAcT2-mTurquoise (gift of K. van Eickels), mTurquoise-PTP1B and ErbB1-mTurquoise (gifts of J. Luig), YFP-dSH2 (two consecutive phosphotyrosine-binding Src-homology 2 domains derived from pp60^c-Src^ described in Kirchner et al. [83]), PTB-mCherry (mCherry version of PTB-YFP described in Offterdinger et al. [84], gift of J. Ibach and P. Verveer), ErbB1-mCitrine (monomeric version of ErbB1-Citrine described in Offterdinger & Bastiaens [91], gift of J. Ibach and P. Verveer) and mCherry-PTP1B^D/A^ (described in Haj et al. [31]).

The following plasmids were constructed for this study. Plasmid pJM24 (mTFP1-PTP1Btail) was constructed by fusion PCR of mTFP1 with PTP1Btail (35 C-terminal amino acids of PTP1B) and insertion into a pcDNA backbone. Plasmid pAK63 (mCherry-PTP1Btail) was constructed by swapping mCherry for mTFP1 in pJM24. Plasmids pAK115 (mCherry-PTP1Btail^VCFH^), Plasmids pAK99 (mCherry-PTP1Btail^ΔHALS^), pAK93 (mCherry-PTP1Btail^Scr^), pAK92 (mCherry-PTP1BtailC) and pAK91 (mCherry-PTP1BtailM) were constructed by PCR of mCherry with overlapping 5′ (NheI-containing) and 3′ (EcoRI-containing) primers that included the entire respective coding regions for the amino acid sequences of PTP1Btail^VCFH^, PTP1Btail^ΔHALS^, PTP1Btail^Scr^, PTP1BtailC and PTP1BtailM (Fig 4). Plasmid pAK101 (mCherry-PTP1Btail^R428E^) was constructed by double point mutation of the codon for arginine in pAK63 (AGG→GAG). Plasmid pAK110 (mCherry-PTP1Btail^F429R^) by double point mutation of the indicated codon for phenylalanine (TTC→CGC). Plasmid pAK108 (mCherry-PTP1Btail^N412I^) was constructed by point mutation of the indicated codon for asparagine (AAC→ATC). Plasmid pAK47 (mCherry-PTP1B^D/A^-ER) was constructed by replacement of mTagBFP with mCherry-PTP1B^D/A^ in mTagBFP-Sec61β (gift from R. Stricker and E. Zamir). Plasmid pAK49 (mCherry-PTP1B^D/A^-OMM) was constructed by replacement of mTagBFP with mCherry-PTP1B^D/A^ in mTagBFP-CytB5mito (gift from R. Stricker and E. Zamir, contains an OMM-targeting mutant of the tail anchor of CytB5 [78]). Plasmid pAK83 (mCherry-PTP1B^D/A^-MAT) was constructed by replacement of mTagBFP with mCherry-PTP1B^D/A^ in COX8a-mTagBFP (gift from R. Stricker and E. Zamir). Plasmid pAK94 (mCherry-PTP1B^D/A^-IMS) was constructed by replacement of N-terminal COX8a sequence in the plasmid mCherry-PTP1B^D/A^-MAT with the N-terminus of the IMS-localizing protein Smac/DIABLO [85]. Plasmids pAK172 (mTurquoise-APEX-PTP1B) and pAK173 (mTFP1-APEX-PTP1tail) were constructed by insertion of *SacI*–flanked PCR inserts containing APEX into the *SacI* sites of mTurquoise-PTP1B and mTFP1-PTP1Btail, respectively. pAK174 (Tom20-APEX-mTurquoise) was similarly obtained by insertion of an *AgeI*–flanked PCR insert containing APEX into the *AgeI* site of Tom20-APEX-mTurquoise.

### Construction of the Yeast Strains

For preparation of competent yeast and their transformation through homologous recombination of PCR products, we followed standard protocols [89]. For homologous recombination-based insertion into the chromosomal leu2 locus of the wild-type strain ESM356-1 [92] we used appropriately modified versions of the primers ISce1-Nat-A and ISce1-Nat-B (generating the leu2Δ0 deletion) described in Khmlenskii et al. [93] PCR-based insertion of the following plasmids generated the corresponding yeast strains: yemCitrine-PTP1Btail (pAK51, sAK199), yemCitrine-Ysy6tail (pAK72, sAK238), yemCitrine-Sec22tail (pAK86, sAK264), yemCitrine-PTP1BtailM (pAK87, sAK257), yemCitrine-PTP1BtailC (pAK88, sAK258), yemCitrine-PTP1Btail^R428E^ (pAK104, sAK277) and yemCitrine-PTP1Btail^F429R^ (pAK111, sAK281). For C-terminal chromosomal tagging of Ste2, Cox4, Sec7, Cwp2 with mCherry in the indicated strains (sAK201, sAK202, sAK203, sAK204, sAK272, sAK273, sAK274), we used plasmid pFA6a-mCherry-KanMX (gift from M. Knop) and appropriate S3 and S2 primers [89] for each targeted locus. For deletions of specific insertion pathway proteins in the indicated strains in S2 Table, we used appropriately designed S1 and S2 primers [89] for PCR of the selection factor-containing plasmids pFA6a-klUra3 (gift from M. Knop), pFA6a-HIS3-Mx6 (gift from M. Knop) and pFA6a-hphNT1 [89].

For transformation of the plasmid pAK100 (p415-yemCitrine-PTP1Btail) into the strains RH2881 and RH6829 [58] to respectively generate the strains pAK275 and pAK276, we used the standard lithium acetate-based protocol [94].

### Transfection, Staining, Fixation and Immunostaining

Fixation, staining and immunostaining of the multiple mammalian cell lines shown in Fig 1 was carried out as follows. COS-7 cells (African green monkey fibroblast-like kidney cells; ATCC CRL-1651), BJ Fibroblast cells (human foreskin; origin described by Hahn et al. [95]), HeLa cells (human cervical cancer; DSMZ ACC 57), MCF7 cells (human breast cancer; ATCC HTB-22), MDCK cells (Madin-Darby canine kidney; ATCC CCL-34) and HepG2 cells (human liver carcinoma; DSMZ ACC 180) were cultured in growth medium: Dulbecco modified Eagle medium (DMEM; PAN) containing 10% fetal bovine serum (FBS), 1% L-glutamine and 1% non-essential amino acids (NEAA). After staining of the cells with MitoTracker Red CMXRos (250 nM; Invitrogen) for 15 minutes at 37°C, they were washed once with phosphate-buffered saline (PBS) and fixed with 4% paraformaldehyde in PBS (pH 7.5) for 5 minutes at room temperature. The fixed cells were washed three times with tris-buffered saline (TBS), permeabilized with 0.1% Triton X-100 in TBS for 5 minutes at room temperature and then washed again three times with TBS. Blocking was achieved by incubation with 2% bovine serum albumin (BSA) in PBS for 30 minutes at room temperature. Next, the primary antibody PTPase 1B (Ab-1) mouse mAB (Fg61G) (Calbiochem, 1:100 dilution) was applied for 60 minutes at room temperature. Unbound antibody was removed by washing three times with PBS. Secondary antibody incubation was performed for 30 minutes at room temperature with Alexa-Fluor-488 chicken anti-mouse IgG (Invitrogen, 1:200 dilution). Finally, cells were washed three times with PBS followed by their observation with confocal microscopy.

COS-7 cells in S1 Fig were transiently transfected with a plasmid containing mTagBFP-Sec61 using Fugene6 (Promega) and then fixed and antibody stained as described above. COS-7 cells shown in Fig 2, S2 Fig, S8 Fig, S9 Fig, S10 Fig and S11 Fig were transiently transfected using Fugene6 (Promega) with the plasmid constructs detailed in the figure captions before their fixation as described above.

COS-7 cells shown in S3 Fig, S12 Fig, S13 Fig, S14 Fig, S15 Fig and S16 Fig, as well as in S1 Movie and S2 Movie, were transiently transfected with the indicated constructs detailed in the figure captions. After 6–8 hours of transfection, the medium was exchanged for growth medium (as described above, S3 Fig and S1 Movie) or starvation medium (DMEM containing phenol red plus 0.1% BSA, S12 Fig, S13 Fig, S14 Fig, S15 Fig, S16 Fig and S2 Movie) overnight. Immediately before live cell monitoring with fluorescence microscopy (described below), the medium was exchanged for imaging medium (low-bicarbonate DMEM without phenol red; PAN). Stimulation of the starved cells was achieved by addition of EGF at a concentration of 100 ng/mL.

### Confocal Microscopy

All fluorescence images shown in this manuscript (aside from the widefield images in Fig 3, see section Electron Microscopy below) were obtained using an Olympus Fluoview^TM^ FV1000 confocal microscope (Olympus Life Science Europa, Hamburg, Germany) equipped with an integrated module for time-domain lifetime measurements (PicoQuant GmbH, Berlin, Germany) and a custom-made environmental chamber maintained at 37°C for the live cell experiments (as the cell media contained HEPES, an additional CO_2_ environment was not necessary). The pinhole was set to 100 μm (roughly one Airy unit) for all images. Continuous 458/488/515 nm lines were produced by an Argon ion laser (Melles Griot, Albuquerque, New Mexico) with an additional 561 nm line was produced by a diode-pumped solid state laser (Melles Griot) in an epifluorescence setting. The integrated module for lifetime measurements consisted of additional five separate pulsed diode lasers (405/440/470/510/532 nm) controlled by a PDL828 “Sepia II” driver. Dichroic elements from Chroma (Rockingham, VT) or Omega Optical (Brattleboro, VT) for detection of the emitted light were chosen with at least 10 nm distance from the relevant excitation wavelength and sufficient distance from the emission profiles of redder fluorophores that may have also been present in the cells. Individual photon arrivals were detected using a SPAD (PDM Series, Micro Photon Devices, Bolzano, Italy) and were recorded by a PicoHarp 300 TCSPC module that could be operated up to a maximum of roughly 10^6^ counts/s. Optimal settings for measuring cells that express all four of the following fluorophores would correspond to the following configuration for our setup: mTagBFP (EX: 405 nm, EM: 420–460 nm), mTurquoise/mTFP1 (EX: 458 nm, EM: 470–490 nm), mCitrine (EX: 510 nm, EM: 524–550 nm) and mCherry (EX: 561 nm, EM: 570–625). All images were obtained at a resolution of either 512×512 or 256×256 and were often additionally linearly contrasted and cropped (and, where indicated, overlaid) within FIJI [96] to generate the final displayed images.

### Fluorescence Lifetime Imaging Microscopy

Fluorescence Lifetime Imaging Microscopy (FLIM) images were obtained and analysed as previously described [90]. For the FLIM images shown in S13 Fig, S14 Fig, S15 Fig and S16 Fig, pulsed light at 510 nm was used to excite mCitrine in cells expressing ErbB1-mCitrine with single emitted photons between 524–550 nm detected using a SPAD and recorded at up to 10^6^ counts/s (see the section above on Confocal Microscopy). The time-correlated single photon counting (TCSPC) histogram for the entire continuous recording (2 min before EGF stimulation plus 14 minutes after) was used to fit (by χ^2^ minimization) the lifetimes of isolated donors (*τ*_1_) and donors undergoing FRET with the acceptor (*τ*_2_), with single pixel FRET fractions α (along with the constant background) fit using “maximum fidelity” [90].

### Pulsed Interleaved Excitation

For S1 Movie, we employed pulsed interleaved excitation (PIE) to quantitatively assess the colocalization of mTurquoise-PTP1B and mCherry-PTP1Btail across live COS-7 cells. PIE was carried out using our Olympus/PicoQuant FLIM setup (described above). Briefly, pulses of 440 nm and 532 nm light were alternated with a 25 ns pulse interval. The emission light was split using a 560 nm dichroic into two SPADs, one with a 460–500 nm emission filter for collecting the mTurquoise fluorescence (SPAD1) and one with a 570–625 nm emission filter for collecting the mCherry fluorescence (SPAD2). As the mTurquoise fluorescence following the 440 nm excitation pulse will also be present in SPAD2, the events required further filtering, which was performed using a modified version of our custom-written analysis code *p*FLIM [90]. Only events in SPAD1 following the 440 nm pulses were retained (mTurquoise-specific fluorescence). Similarly, only events in SPAD2 following the 532 nm pulses were retained (mCherry-specific fluorescence). This filtering removed the crosstalk between the two channels (as can be seen from our negative control cells in S1 Movie, for which the Golgi marker GalNAcT2-mTurquoise was coexpressed with mCherry-PTP1Btail). The final events in each SPAD were then used to generate the individual frames for each fluorophore shown in S1 Movie. The red/green overlay images were generated using FIJI [96] and provide a quantitative and robust measure of the instantaneous ratio of each fluorophore in each pixel.

### Electron Microscopy

The electron microscopy imaging of DAB-stained COS-7 cells using the engineered APEX (ascorbate peroxidase) were carried out as previously described [48]. Briefly, COS-7 cells transfected using Lipofectamine 2000 (Invitrogen, Germany) with plasmids containing either mTurquoise-APEX-PTP1B, mTFP1-APEX-PTP1Btail or Tom20-APEX-mTurquoise were washed with prewarmed PBS (phosphate-buffered saline, pH 7.4) and fixed using a mixture of glutaraldehyde (1%) and paraformaldehyde (2%). Cells were rinsed 3× with PBS and incubated with the blocking solution containing 100 mM glycine in PBS for 20 min to quench unreactive fixative. For the subsequent APEX development, cells were washed with PBS (pH 7.4) three times and then incubated in freshly prepared buffered solution of 2 mg/ml DAB (3,3’-diaminobenzidine tertahydrochloride, Polysciences, Germany). Cells were subsequently post-fixed with 1% osmium tetroxide reduced by 1.5% potassium ferrocyanide for 30 minutes. Specimens were washed with distilled water for 3 min and dehydrated using a graded ethanol series (50%, 70%, 90%, 3x 100%) for 3 min for each step. Finally, cells were embedded in Epon 812 (Serva, Germany). The area of interest was trimmed and cut on the Ultracut S microtome (Leica, Germany) to get thin (70 nm) slices. Samples were examined on a JEOL-1400 transmission electron microscope (80 kV, JEOL, Japan) equipped with a 2k CCD camera (TVIPS GmbH, Germany).

## Supporting Information

S1 Fig Mitochondrial versus ER localization of endogenous PTP1B in COS-7 cells. COS-7 cells were prepared for immunostaining as in Fig 1 with the only difference being their additional expression of an ER marker (mTagBFP-Sec61). In the top row, the mitochondrial localization of endogenous PTP1B is revealed through its colocalization with the mitochondrial stain MitoTracker Red CMXRos (Invitrogen). In the second row and the third row (zoomed-in view of the white box in the second row), the discrepancy in the overlay of the PTP1B signal and the ER marker allows identification of membrane-bound PTP1B that is apparently not in proximity to the ER. The structure indicated with an arrow clearly colocalizes with the mitochondria in the top row but not with the ER in the second and third rows. Scale bars: 20 μm and 5 μm, respectively.
(PDF)

S2 Fig Subcellular partitioning of PTP1B is completely determined by its tail anchor. (A) COS-7 cells expressing mCitrine-PTP1B (green), mCherry-PTP1Btail (red) and Tom20-mTagBFP (cyan) were visualized by confocal microscopy. The lower right image represents the overlay of the mCitrine-PTP1B (green) and mCherry-PTP1Btail (red) images. As cells were transiently transfected, not all constructs were expressed in all cells (the cell occupying the upper left corner, for example, does not express the mCitrine-PTP1B construct). (B) COS-7 cells expressing mTFP1-PTP1Btail (green), mCherry-PTP1Btail^VCFH^ (red) and Tom20-mTagBFP (cyan) were visualized by confocal microscopy. The lower right image represents the overlay of the mTFP1-PTP1Btail (green) and mCherry-PTP1Btail^VCFH^ (red) images. Scale bars: 20 μm.
(PDF)

S3 Fig Closer examination of the distribution of the PTP1B tail anchor along the ER. Confocal microscopy of a COS-7 cell expressing mCherry-PTP1Btail (green) and the ER lumenal marker mTFP1-ER (red). The overlay of both channels is also displayed. Especially clear regions of overlap (free from mitochondria) are highlighted (boxes). Scale bar: 10 μm.
(PDF)

S4 Fig Effects of PTP1B overexpression on ER and mitochondrial morphologies. (A) Overexpression of mTurquoise-APEX-PTP1B causes aggregation of mitochondrial and ER membranes. The large feature (upper left) is consistent with self-aggregation of the ER. Mitochondrial cristae (inner mitochondrial membrane) are not stained (black arrowhead). (B) Overexpression of mTurquoise-APEX-PTP1B leads as well to alteration of the mitochondrial interior in many mitochondria (black arrowhead). The white arrows indicate regions of higher staining along the ER that are in direct apposition to mitochondria (ER MAM sites). Scale bars: 500 nm.
(PDF)

S5 Fig Predicted targeting of the PTP1B tail anchor in yeast based on its hydropathy. A library of previously identified C-terminal tail-anchor-containing proteins in yeast is displayed based on the list of Burri & Lithgow^55^ (with additional inclusion of Gem1^57^ in the mitochondria panel) along with the list of 56 GPI-anchored proteins given in Ast et al.^58^, which were removed from the list of Burri & Lithgow due to their GPI anchorage. The hydropathies of the C termini of these proteins are displayed based on the Kyte-Doolittle method^56^ (boxcar smoothing of n = 7), with all protein sequences centered at the amino acid position corresponding to peak hydropathy. The hydropathy profile of the PTP1B tail anchor is shown in all figures (black). In the bottom right panel, the hydropathy profiles of canonical tail anchor proteins from yeast (Fis1, Ysy6, Sec22, Gas1) and from mammalian cells (Bcl2, CytB5, Sec61β, VAMP-1B) are shown, as well as the high hydropathy tail isoform PTP1B^N412I^ (N412I, which has not been shifted to its maximum hydropathy, but is instead in register with the PTP1B tail).
(PDF)

S6 Fig Subcellular partitioning of the PTP1B tail anchor in yeast. Confocal microscopy of specific strains of *S*. *cerevisiae* (ESM356-1 background strain[92]) that chromosomally express yemCitrine-PTP1Btail and markers for either the ER (Cwp2-mCherry), vacuole (Ste2-mCherry), mitochondria (Cox4-mCherry) or Golgi (Sec7-mCherry). Scale bar: 20 μm.
(PDF)

S7 Fig Dependence of the targeting of the PTP1B tail anchor on the type of sterol. A wild-type strain of the yeast *S*. *cerevisiae* that produces ergosterol (RH2881^63^) as well as a mutant strain that produces cholesterol as its dominant sterol (RH6829^63^), with membrane composition therefore more similar to mammalian cells, were transformed with the plasmid p415-yemCitrine-PTP1Btail. In both strains, yemCitrine-PTP1Btail localized to the perinuclear/cortical membranes of the ER and to the vacuolar membrane. Scale bars: 5 μm.
(PDF)

S8 Fig Localization of N- and/or C-terminal truncations of the PTP1B tail anchor in COS-7 cells and in yeast. (A–E) Coexpression of the N-terminally truncated and fluorophore-labeled PTP1BtailC (Fig 4) in COS-7 cells (mCherry-PTP1BtailC) along with either the mitochondrial marker (left label, “Mito”) Tom20-mTagBFP (A) or the Golgi marker (“Golgi”) GalNAcT2-mTurquoise (B); and in yeast cells (yemCitrine-PTP1BtailC) along with either the ER marker (“ER”) Cwp2-mCherry (C), the mitochondrial marker (“Mito”) Cox4-mCherry (D) or the Golgi marker (“Golgi”) Sec7-mCherry (E). (F–J) Similar coexpression of the N- and C-terminally truncated and fluorophore-labeled PTP1BtailM (Fig 4) in COS-7 cells (mCherry-PTP1BtailM, F and G) and in yeast (yemCitrine-PTP1BtailM, H–J). Scale bars: 20 μm.
(PDF)

S9 Fig Localization of charge-altered isoforms of the PTP1B tail anchor in COS-7 cells and in yeast. (A,B) Coexpression of the fluorphore-labeled, negatively charged tail isoform PTP1Btail^R428E^ (Fig 4) in COS-7 cells (mCherry-PTP1Btail^R428E^) along with the mitochondrial marker (left label, “Mito”) Tom20-mTagBFP (A) and in yeast cells (yemCitrine-PTP1Btail^R428E^) along with the mitochondrial marker Cox4-mCherry (B). (C,D) Coexpression of the fluorophore-labeled, highly positively charged tail isoform PTP1Btail^F429R^ (Fig 4) in COS-7 cells (mCherry-PTP1Btail^F429R^) along with the mitochondrial marker Tom20-mTagBFP (C) and in yeast cells (yemCitrine-PTP1Btail^F429R^) along with the mitochondrial marker Cox4-mCherry (D). Scale bars: 20 μm.
(PDF)

S10 Fig Localization of a PTP1B tail isoform with high hydropathy in COS-7 cells. The wild-type tail anchor chimera mTFP1-PTP1Btail is shown (green) alongside tail isoform chimera mCherry-PTP1Btail^N412I^ having higher hydropathy (red) in COS-7 cells. Their red/green overlay is also displayed as well as the mitochondrial marker Tom20-mTagBFP. Scale bar: 20 μm.
(PDF)

S11 Fig Localization of a scrambled PTP1B tail isoform in COS-7 cells. (A) Subcellular partitioning of a slightly truncated tail isoform PTP1Btail^ΔHALS^ (Fig 4, red) and the original tail anchor (green) were identical (see overlay), with both accumulating strongly at the mitochondria (as marked using Tom20-mTagBFP, cyan). Differences at the cell peripheries are due to optical refraction artifacts. The hydropathy profiles of the PTP1Btail^ΔHALS^ (black) and a scrambled isoform PTP1Btail^Scr^ (Fig 4, dashed magenta) are also shown. (B–C) Coexpression of the fluorophore-labeled PTP1Btail^Scr^ in COS-7 cells (mCherry-PTP1Btail^Scr^) along with either the mitochondrial marker Tom20-mTagBFP (B) or the Golgi marker GalNAcT2-mTurquoise (C). Scale bars: 20 μm.
(PDF)

S12 Fig Visualizing mitochondrial phosphotyrosine before and after EGF stimulation. COS-7 cells expressing dSH2-YFP, PTB-mCherry and the mitochondrial marker Tom20-mTagBFP were starved and imaged before (0 min) and after (5 min and 20 min) their stimulation with EGF (100 ng/mL). No mitochondrial localization of dSH2-YFP was observed. In contrast, a basal accumulation of PTB-mCherry at the mitochondria was observed that increased following EGF stimulation. Scale bar: 20 μm.
(PDF)

S13 Fig Dynamic FLIM-based monitoring of the subcellular interaction of ErbB1-mCitrine with mCherry-PTP1B^D/A^ in COS-7 cells following EGF stimulation. Donor lifetime images of ErbB1-mCitrine are shown before (-2 min to 0 min) and after (2 min to 4 min, 12 min to 14 min) stimulation with EGF in cells expressing the donor-labeled ErbB1 and the mitochondrial marker Tom20-mTagBFP in the first and second rows (lifetime control cells, representative of n = 3 recordings). In the column “Histogram/Acceptor”, the corresponding TCSPC histograms of the donor lifetime are shown (“Res” stands for the normalized fit residuals, see Walther et al. for further details^90^) and acceptor images are also displayed (acquired immediately after recording of the donor data). A lifetime of 3.02 ns was obtained from fitting the histogram of the entire 16-minute recording, assuming a fixed donor-only lifetime of 3.05 ns (blue TCSPC histogram) and a FRET lifetime of 1.51 ns (orange TCSPC histogram), which were determined from double lifetime fitting of the cells shown in the third and fourth rows. A generally low FRET fraction (consistent with zero) was obtained across the images. FLIM images of COS-7 cells expressing ErbB1-mCitrine, mCherry-PTP1B^D/A^ and Tom20-mTagBFP are shown in the third and fourth rows (representative of n = 5 recordings). The image of acceptor mCherry-PTP1B^D/A^ is given in the bottom row and fourth column (“Histogram/Acceptor”) to be compared with the Tom20-mTagBFP image to its right. Here, a basal interaction (of varying strength) is detected that increases in all of the cells after EGF stimulation. An average lifetime of 2.78 ns over the entire 16-minute movie was obtained, which was significantly lower than the 3.02 ns lifetime of the donor-only control cells. A specific decrease in lifetime at the mitochondria (arrows) was clearly observed in the cells on the right. The Tom20-mTagBFP mislocalized in the cell on the left (large aggregate) preventing determination of the mitochondria in this cell. Color scale in the top left image gives the FRET fraction α. The “intensity weighted” images of the donor in the second and fourth rows correspond to an additional weighting of the respective images in the first and third rows by the observed donor counts in each pixel^90^. Scale bars: 30 μm.
(PDF)

S14 Fig Control for dynamic FLIM-based monitoring of subcellular ErbB1 interaction with PTP1B^D/A^. Donor lifetime images of COS-7 cells expressing ErbB1-mCitrine (donor), mCherry-PTP1Btail (acceptor) and the mitochondrial marker Tom20-mTagBFP (representative of n = 3 recordings, see S13 Fig for further details). The average lifetime of the entire 16 minute recording was 2.98 ns. A generally low FRET fraction α across the cells was detected that was similar to the negative control shown in the first two rows of S13 Fig. Despite the similar localization and expression of the tail-only acceptor-labeled chimera, no lifetime reduction either before or after EGF stimulation was detectable of the donor-labeled ErbB1-mCitrine. This control, therefore, importantly demonstrates that the reduced lifetime in the bottom two rows of S13 Fig reflects the direct interaction of ErbB1-mCitrine with the catalytic domain of mCherry-labeled PTP1B^D/A^. Scale bar: 30 μm.
(PDF)

S15 Fig Dynamic FLIM-based monitoring of the interaction of ErbB1-mCitrine with mCherry-labeled PTP1B^D/A^ targeted to either the ER or the outer mitochondrial membrane. In the first two rows, donor lifetime images of COS-7 cells expressing ErbB1-mCitrine, mCherry-PTP1B^D/A^-ER and the mitochondrial marker Tom20-mTagBFP are displayed before and after EGF stimulation (representative of n = 3 recordings, see S13 Fig for further details). A robust decrease in lifetime was detectable upon EGF stimulation, revealing the specific interaction of ErbB1 with ER-localized PTP1B. In the third and fourth rows, donor lifetime images of COS-7 cells expressing ErbB1-mCitrine, mCherry-PTP1B^D/A^-OMM and the mitochondrial marker Tom20-mTagBFP are displayed before and after EGF stimulation (representative of n = 4 recordings). A robust decrease in lifetime was detected upon EGF stimulation only in the vicinity of the mitochondria (arrows; note the high degree of overlap of the mCherry-PTP1B^D/A^-OMM construct and the mitochondrial marker Tom20-mTagBFP). Despite the only slight reduction of the lifetime of the entire 16 minute recording (2.89 ns for the first two rows, 2.96 ns for the last two rows), local lifetime reductions were clearly detected that coincided with the acceptor localization (ER in top two rows, mitochondria in bottom two rows). Scale bars: 30 μm.
(PDF)

S16 Fig Dynamic FLIM-based monitoring of the interaction of ErbB1-mCitrine with mCherry-labeled PTP1B^D/A^ targeted to either the intermembrane space (IMS) or the mitochondrial matrix (MAT). In the first two rows, donor lifetime images of COS-7 cells expressing ErbB1-mCitrine, mCherry-PTP1B^D/A^-IMS and the mitochondrial marker Tom20-mTagBFP are displayed before and after EGF stimulation (representative of n = 8 recordings, see S13 Fig for further details). No significant decrease in lifetime was detectable upon EGF stimulation, either generally across the cell or specifically at the mitochondria (arrows), and also no recruitment of ErbB1 to the mitochondria. In the third and fourth rows, donor lifetime images of COS-7 cells expressing ErbB1-mCitrine, mCherry-PTP1B^D/A^-MAT and the mitochondrial marker Tom20-mTagBFP are displayed before and after EGF stimulation (representative of n = 8 recordings). While there was a slightly decreased lifetime across some cells (reflected by the slightly lower lifetime of 2.95 ns obtained from fitting the entire histogram corresponding to the 16 minute recording), there was no additional decrease at the mitochondria (arrows) and also no observed recruitment of ErbB1 to the mitochondria. Scale bars: 30 μm.
(PDF)

S1 Movie Movie of mTurquoise-PTP1B and mCherry-PTP1Btail. COS-7 cells expressing mTurquoise-PTP1B and mCherry-PTP1Btail (top row) or GalNAcT2-mTurquoise and mCherry-PTP1Btail (bottom row) were confocally imaged using pulsed-interleaved excitation (see Materials and Methods). The cell was continuously tracked for 6 minutes (10 seconds/frame; 60 frames total). The width of the images corresponds to either 132.5 μm (top row) or 151.4 μm (bottom row).
(AVI)

S2 Movie Movie of the FRET-based interaction of ErbB1-mCitrine with mCherry-PTP1B^D/A^ as measured with FLIM. Each frame corresponds to 2 minutes. The individual frames before stimulation (−2 min to 0 min) and after stimulation (2 min to 4 min, 12 min to 14 min) are identical to those shown in the fourth row of S13 Fig (color scale represents the FRET fraction α).
(AVI)

S1 Table Localization in COS-7 (C) and yeast (Y) of different fluorophore-labeled PTP1B constructs. (PDF)

S2 Table Yeast strains. All strains with an AK prefix—and therefore not ESM356-1^92^, RH2881^63^ and RH6829^63^—were generated for this study.
(PDF)

## References

[1] Tonks NK, Diltz CD, Fischer EH. Purification of the major protein-tyrosine-phosphatases of human placenta. J Biol Chem. 1988;263: 6722–6730. 2834386

[2] Tonks NK, Diltz CD, Fischer EH. Characterization of the major protein-tyrosine-phosphatases of human placenta. J Biol Chem. 1988;263: 6731–6737. 2834387

[3] Brown-Shimer S, Johnson KA, Lawrence JB, Johnson C, Bruskin A, Green NR, et al Molecular cloning and chromosome mapping of the human gene encoding protein phosphotyrosyl phosphatase 1B. Proc Natl Acad Sci U S A. 1990;87: 5148–5152. 2164224 10.1073/pnas.87.13.5148 PMC54279

[4] Yip S-C, Saha S, Chernoff J. PTP1B: a double agent in metabolism and oncogenesis. Trends Biochem Sci. 2010;35: 442–449. 10.1016/j.tibs.2010.03.004 20381358 PMC2917533

[5] Chernoff J, Schievella AR, Jost CA, Erikson RL, Neel BG. Cloning of a cDNA for a major human protein-tyrosine-phosphatase. Proc Natl Acad Sci. 1990;87: 2735–2739. 2157211 10.1073/pnas.87.7.2735 PMC53765

[6] Combs AP. Recent Advances in the Discovery of Competitive Protein Tyrosine Phosphatase 1B Inhibitors for the Treatment of Diabetes, Obesity, and Cancer. J Med Chem. 2010;53: 2333–2344. 10.1021/jm901090b 20000419

[7] Lessard L, Stuible M, Tremblay ML. The two faces of PTP1B in cancer. Biochim Biophys Acta BBA—Proteins Proteomics. 2010;1804: 613–619. 10.1016/j.bbapap.2009.09.018 19782770

[8] Shifrin VI, Neel BG. Growth factor-inducible alternative splicing of nontransmembrane phosphotyrosine phosphatase PTP-1B pre-mRNA. J Biol Chem. 1993;268: 25376–25384. 8244970

[9] Guan KL, Haun RS, Watson SJ, Geahlen RL, Dixon JE. Cloning and expression of a protein-tyrosine-phosphatase. Proc Natl Acad Sci. 1990;87: 1501–1505. 10.1073/pnas.87.4.1501 2154749 PMC53503

[10] Frangioni JV, Beahm PH, Shifrin V, Jost CA, Neel BG. The nontransmembrane tyrosine phosphatase PTP-1B localizes to the endoplasmic reticulum via its 35 amino acid C-terminal sequence. Cell. 1992;68: 545–560. 10.1016/0092-8674(92)90190-N 1739967

[11] Flint AJ, Tiganis T, Barford D, Tonks NK. Development of “substrate-trapping” mutants to identify physiological substrates of protein tyrosine phosphatases. Proc Natl Acad Sci U S A. 1997;94: 1680–1685. 9050838 10.1073/pnas.94.5.1680 PMC19976

[12] Anderie I, Schulz I, Schmid A. Characterization of the C-terminal ER membrane anchor of PTP1B. Exp Cell Res. 2007;313: 3189–3197. 10.1016/j.yexcr.2007.05.025 17643420

[13] Brambillasca S, Yabal M, Makarow M, Borgese N. Unassisted translocation of large polypeptide domains across phospholipid bilayers. J Cell Biol. 2006;175: 767–777. 10.1083/jcb.200608101 17130291 PMC2064676

[14] Rabu C, Wipf P, Brodsky JL, High S. A Precursor-specific Role for Hsp40/Hsc70 during Tail-anchored Protein Integration at the Endoplasmic Reticulum. J Biol Chem. 2008;283: 27504–27513. 10.1074/jbc.M804591200 18667436 PMC2562055

[15] Abell BM, Rabu C, Leznicki P, Young JC, High S. Post-translational integration of tail-anchored proteins is facilitated by defined molecular chaperones. J Cell Sci. 2007;120: 1743–1751. 10.1242/jcs.002410 17456552

[16] Schuldiner M, Collins SR, Thompson NJ, Denic V, Bhamidipati A, Punna T, et al Exploration of the Function and Organization of the Yeast Early Secretory Pathway through an Epistatic Miniarray Profile. Cell. 2005;123: 507–519. 10.1016/j.cell.2005.08.031 16269340

[17] Stefanovic S, Hegde RS. Identification of a Targeting Factor for Posttranslational Membrane Protein Insertion into the ER. Cell. 2007;128: 1147–1159. 10.1016/j.cell.2007.01.036 17382883

[18] Schuldiner M, Metz J, Schmid V, Denic V, Rakwalska M, Schmitt HD, et al The GET Complex Mediates Insertion of Tail-Anchored Proteins into the ER Membrane. Cell. 2008;134: 634–645. 10.1016/j.cell.2008.06.025 18724936 PMC2572727

[19] Borgese N, Fasana E. Targeting pathways of C-tail-anchored proteins. Biochim Biophys Acta BBA—Biomembr. 2011;1808: 937–946. 10.1016/j.bbamem.2010.07.010 20646998

[20] Wang F, Whynot A, Tung M, Denic V. The Mechanism of Tail-Anchored Protein Insertion into the ER Membrane. Mol Cell. 2011;43: 738–750. 10.1016/j.molcel.2011.07.020 21835666 PMC3614002

[21] Hegde RS, Keenan RJ. Tail-anchored membrane protein insertion into the endoplasmic reticulum. Nat Rev Mol Cell Biol. 2011;12: 787–798. 10.1038/nrm3226 22086371 PMC3760496

[22] Chartron JW, Clemons WM Jr, Suloway CJ. The complex process of GETting tail-anchored membrane proteins to the ER. Curr Opin Struct Biol. 2012;22: 217–224. 10.1016/j.sbi.2012.03.001 22444563 PMC3359790

[23] Denic V. A portrait of the GET pathway as a surprisingly complicated young man. Trends Biochem Sci. 2012;37: 411–417. 10.1016/j.tibs.2012.07.004 22951232 PMC3459580

[24] Keenan RJ, Freymann DM, Stroud RM, Walter P. The Signal Recognition Particle. Annu Rev Biochem. 2001;70: 755–775. 10.1146/annurev.biochem.70.1.755 11395422

[25] Lang S, Benedix J, Fedeles SV, Schorr S, Schirra C, Schäuble N, et al Different effects of Sec61α, Sec62 and Sec63 depletion on transport of polypeptides into the endoplasmic reticulum of mammalian cells. J Cell Sci. 2012;125: 1958–1969. 10.1242/jcs.096727 22375059 PMC4074215

[26] Johnson N, Powis K, High S. Post-translational translocation into the endoplasmic reticulum. Biochim Biophys Acta BBA—Mol Cell Res. 2013;1833: 2403–2409. 10.1016/j.bbamcr.2012.12.008 23266354

[27] Haj FG, Verveer PJ, Squire A, Neel BG, Bastiaens PIH. Imaging Sites of Receptor Dephosphorylation by PTP1B on the Surface of the Endoplasmic Reticulum. Science. 2002;295: 1708–1711. 10.1126/science.1067566 11872838

[28] Yudushkin IA, Schleifenbaum A, Kinkhabwala A, Neel BG, Schultz C, Bastiaens PIH. Live-Cell Imaging of Enzyme-Substrate Interaction Reveals Spatial Regulation of PTP1 B. Science. 2007;315: 115–119. 10.1126/science.1134966 17204654

[29] Hernández MV, Sala MGD, Balsamo J, Lilien J, Arregui CO. ER-bound PTP1B is targeted to newly forming cell-matrix adhesions. J Cell Sci. 2006;119: 1233–1243. 10.1242/jcs.02846 16522684

[30] Burdisso JE, González Á, Arregui CO. PTP1B promotes focal complex maturation, lamellar persistence and directional migration. J Cell Sci. 2013;126: 1820–1831. 10.1242/jcs.118828 23444382

[31] Haj FG, Sabet O, Kinkhabwala A, Wimmer-Kleikamp S, Roukos V, Han H-M, et al Regulation of Signaling at Regions of Cell-Cell Contact by Endoplasmic Reticulum-Bound Protein-Tyrosine Phosphatase 1B. PLoS ONE. 2012;7: e36633 10.1371/journal.pone.0036633 22655028 PMC3360045

[32] Augereau O, Claverol S, Boudes N, Basurko M-J, Bonneu M, Rossignol R, et al Identification of tyrosine-phosphorylated proteins of the mitochondrial oxidative phosphorylation machinery. Cell Mol Life Sci. 2005;62: 1478–1488. 10.1007/s00018-005-5005-7 15924266 PMC11139224

[33] Arachiche A, Augereau O, Decossas M, Pertuiset C, Gontier E, Letellier T, et al Localization of PTP-1B, SHP-2, and Src Exclusively in Rat Brain Mitochondria and Functional Consequences. J Biol Chem. 2008;283: 24406–24411. 10.1074/jbc.M709217200 18583343 PMC3259839

[34] Cesaro L, Salvi M. Mitochondrial tyrosine phosphoproteome: New insights from an up‐to‐date analysis. BioFactors. 2010;36: 437–450. 10.1002/biof.123 21072759

[35] Salvi M, Brunati AM, Toninello A. Tyrosine phosphorylation in mitochondria: A new frontier in mitochondrial signaling. Free Radic Biol Med. 2005;38: 1267–1277. 10.1016/j.freeradbiomed.2005.02.006 15855046

[36] Salvi M, Brunati AM, Bordin L, La Rocca N, Clari G, Toninello A. Characterization and location of Src-dependent tyrosine phosphorylation in rat brain mitochondria. Biochim Biophys Acta BBA—Mol Cell Res. 2002;1589: 181–195. 10.1016/S0167-4889(02)00174-X 12007793

[37] Miyazaki T, Neff L, Tanaka S, Horne WC, Baron R. Regulation of cytochrome c oxidase activity by c-Src in osteoclasts. J Cell Biol. 2003;160: 709–718. 10.1083/jcb.200209098 12615910 PMC2173369

[38] Tibaldi E, Brunati AM, Massimino ML, Stringaro A, Colone M, Agostinelli E, et al Src‐Tyrosine kinases are major agents in mitochondrial tyrosine phosphorylation. J Cell Biochem. 2008;104: 840–849. 10.1002/jcb.21670 18247338

[39] Hébert Chatelain E, Dupuy J-W, Letellier T, Dachary-Prigent J. Functional impact of PTP1B-mediated Src regulation on oxidative phosphorylation in rat brain mitochondria. Cell Mol Life Sci. 2010;68: 2603–2613. 10.1007/s00018-010-0573-6 21063895 PMC11115002

[40] Zang QS, Martinez B, Yao X, Maass DL, Ma L, Wolf SE, et al Sepsis-Induced Cardiac Mitochondrial Dysfunction Involves Altered Mitochondrial-Localization of Tyrosine Kinase Src and Tyrosine Phosphatase SHP2. PLoS ONE. 2012;7: e43424 10.1371/journal.pone.0043424 22952679 PMC3428365

[41] Boerner JL, Demory ML, Silva C, Parsons SJ. Phosphorylation of Y845 on the Epidermal Growth Factor Receptor Mediates Binding to the Mitochondrial Protein Cytochrome c Oxidase Subunit II. Mol Cell Biol. 2004;24: 7059–7071. 10.1128/MCB.24.16.7059-7071.2004 15282306 PMC479738

[42] Demory ML, Boerner JL, Davidson R, Faust W, Miyake T, Lee I, et al Epidermal Growth Factor Receptor Translocation to the Mitochondria. J Biol Chem. 2009;284: 36592–36604. 10.1074/jbc.M109.000760 19840943 PMC2794774

[43] Ding Y, Liu Z, Desai S, Zhao Y, Liu H, Pannell LK, et al Receptor tyrosine kinase ErbB2 translocates into mitochondria and regulates cellular metabolism. Nat Commun. 2012;3: 1271 10.1038/ncomms2236 23232401 PMC3521558

[44] Salvi M, Stringaro A, Brunati AM, Agostinelli E, Arancia G, Clari G, et al Tyrosine phosphatase activity in mitochondria: presence of Shp-2 phosphatase in mitochondria. Cell Mol Life Sci CMLS. 2004;61: 2393–2404. 10.1007/s00018-004-4211-z 15378208 PMC11138707

[45] Giorgi C, De Stefani D, Bononi A, Rizzuto R, Pinton P. Structural and functional link between the mitochondrial network and the endoplasmic reticulum. Int J Biochem Cell Biol. 2009;41: 1817–1827. 10.1016/j.biocel.2009.04.010 19389485 PMC2731816

[46] Lebiedzinska M, Szabadkai G, Jones AWE, Duszynski J, Wieckowski MR. Interactions between the endoplasmic reticulum, mitochondria, plasma membrane and other subcellular organelles. Int J Biochem Cell Biol. 2009;41: 1805–1816. 10.1016/j.biocel.2009.02.017 19703651

[47] Rowland AA, Voeltz GK. Endoplasmic reticulum–mitochondria contacts: function of the junction. Nat Rev Mol Cell Biol. 2012;13: 607–625. 10.1038/nrm3440 22992592 PMC5111635

[48] Martell JD, Deerinck TJ, Sancak Y, Poulos TL, Mootha VK, Sosinsky GE, et al Engineered ascorbate peroxidase as a genetically encoded reporter for electron microscopy. Nat Biotechnol. 2012;30: 1143–1148. 10.1038/nbt.2375 23086203 PMC3699407

[49] Snapp EL, Hegde RS, Francolini M, Lombardo F, Colombo S, Pedrazzini E, et al Formation of stacked ER cisternae by low affinity protein interactions. J Cell Biol. 2003;163: 257–269. 10.1083/jcb.200306020 14581454 PMC2173526

[50] Burri L, Lithgow T. A Complete Set of SNAREs in Yeast. Traffic. 2004;5: 45–52. 10.1046/j.1600-0854.2003.00151.x 14675424

[51] Kyte J, Doolittle RF. A simple method for displaying the hydropathic character of a protein. J Mol Biol. 1982;157: 105–132. 7108955 10.1016/0022-2836(82)90515-0

[52] Frederick RL, McCaffery JM, Cunningham KW, Okamoto K, Shaw JM. Yeast Miro GTPase, Gem1p, regulates mitochondrial morphology via a novel pathway. J Cell Biol. 2004;167: 87–98. 10.1083/jcb.200405100 15479738 PMC2172521

[53] Ast T, Cohen G, Schuldiner M. A Network of Cytosolic Factors Targets SRP-Independent Proteins to the Endoplasmic Reticulum. Cell. 2013;152: 1134–1145. 10.1016/j.cell.2013.02.003 23452858

[54] Krajewski S, Tanaka S, Takayama S, Schibler MJ, Fenton W, Reed JC. Investigation of the subcellular distribution of the bcl-2 oncoprotein: residence in the nuclear envelope, endoplasmic reticulum, and outer mitochondrial membranes. Cancer Res. 1993;53: 4701–4714. 8402648

[55] Lithgow T, van Driel R, Bertram JF, Strasser A. The protein product of the oncogene bcl-2 is a component of the nuclear envelope, the endoplasmic reticulum, and the outer mitochondrial membrane. Cell Growth Differ. 1994;5: 411 8043515

[56] Egan B, Beilharz T, George R, Isenmann S, Gratzer S, Wattenberg B, et al Targeting of tail-anchored proteins to yeast mitochondria in vivo. FEBS Lett. 1999;451: 243–248. 10.1016/S0014-5793(99)00581-5 10371198

[57] Cournia Z, Ullmann GM, Smith JC. Differential Effects of Cholesterol, Ergosterol and Lanosterol on a Dipalmitoyl Phosphatidylcholine Membrane: A Molecular Dynamics Simulation Study. J Phys Chem B. 2007;111: 1786–1801. 10.1021/jp065172i 17261058

[58] Souza CM, Schwabe TME, Pichler H, Ploier B, Leitner E, Guan XL, et al A stable yeast strain efficiently producing cholesterol instead of ergosterol is functional for tryptophan uptake, but not weak organic acid resistance. Metab Eng. 2011;13: 555–569. 10.1016/j.ymben.2011.06.006 21741494

[59] Bretscher MS, Munro S. Cholesterol and the Golgi apparatus. Science. 1993;261: 1280–1281. 8362242 10.1126/science.8362242

[60] Brambillasca S, Yabal M, Soffientini P, Stefanovic S, Makarow M, Hegde RS, et al Transmembrane topogenesis of a tail-anchored protein is modulated by membrane lipid composition. EMBO J. 2005;24: 2533–2542. 10.1038/sj.emboj.7600730 15973434 PMC1176458

[61] Setoguchi K, Otera H, Mihara K. Cytosolic factor- and TOM-independent import of C-tail-anchored mitochondrial outer membrane proteins. EMBO J. 2006;25: 5635–5647. 10.1038/sj.emboj.7601438 17110923 PMC1698885

[62] Kemper C, Habib SJ, Engl G, Heckmeyer P, Dimmer KS, Rapaport D. Integration of tail-anchored proteins into the mitochondrial outer membrane does not require any known import components. J Cell Sci. 2008;121: 1990–1998. 10.1242/jcs.024034 18495843

[63] Endo T, Yamano K. Multiple pathways for mitochondrial protein traffic. Biol Chem. 2009;390: 723–730. 10.1515/BC.2009.087 19453276

[64] Dukanovic J, Rapaport D. Multiple pathways in the integration of proteins into the mitochondrial outer membrane. Biochim Biophys Acta BBA—Biomembr. 2011;1808: 971–980. 10.1016/j.bbamem.2010.06.021 20599689

[65] Yamamoto Y, Sakisaka T. Molecular Machinery for Insertion of Tail-Anchored Membrane Proteins into the Endoplasmic Reticulum Membrane in Mammalian Cells. Mol Cell. 2012;48: 387–397. 10.1016/j.molcel.2012.08.028 23041287

[66] Chang Y-W, Chuang Y-C, Ho Y-C, Cheng M-Y, Sun Y-J, Hsiao C-D, et al Crystal Structure of Get4-Get5 Complex and Its Interactions with Sgt2, Get3, and Ydj1. J Biol Chem. 2010;285: 9962–9970. 10.1074/jbc.M109.087098 20106980 PMC2843242

[67] Vilardi F, Lorenz H, Dobberstein B. WRB is the receptor for TRC40/Asna1-mediated insertion of tail-anchored proteins into the ER membrane. J Cell Sci. 2011;124: 1301–1307. 10.1242/jcs.084277 21444755 PMC3115773

[68] Mariappan M, Mateja A, Dobosz M, Bove E, Hegde RS, Keenan RJ. The mechanism of membrane-associated steps in tail-anchored protein insertion. Nature. 2011;477: 61–66. 10.1038/nature10362 21866104 PMC3760497

[69] Abell BM, Pool MR, Schlenker O, Sinning I, High S. Signal recognition particle mediates post-translational targeting in eukaryotes. EMBO J. 2004;23: 2755–2764. 10.1038/sj.emboj.7600281 15229647 PMC514945

[70] Ogg SC, Poritz MA, Walter P. Signal recognition particle receptor is important for cell growth and protein secretion in Saccharomyces cerevisiae. Mol Biol Cell. 1992;3: 895–911. 1327299 10.1091/mbc.3.8.895 PMC275647

[71] Lakkaraju AKK, Thankappan R, Mary C, Garrison JL, Taunton J, Strub K. Efficient secretion of small proteins in mammalian cells relies on Sec62-dependent posttranslational translocation. Mol Biol Cell. 2012;23: 2712–2722. 10.1091/mbc.E12-03-0228 22648169 PMC3395660

[72] Yabal M, Brambillasca S, Soffientini P, Pedrazzini E, Borgese N, Makarow M. Translocation of the C Terminus of a Tail-anchored Protein across the Endoplasmic Reticulum Membrane in Yeast Mutants Defective in Signal Peptide-driven Translocation. J Biol Chem. 2003;278: 3489–3496. 10.1074/jbc.M210253200 12446686

[73] Colombo SF, Longhi R, Borgese N. The role of cytosolic proteins in the insertion of tail-anchored proteins into phospholipid bilayers. J Cell Sci. 2009;122: 2383–2392. 10.1242/jcs.049460 19531581

[74] Pedrazzini E, Villa A, Borgese N. A mutant cytochrome b5 with a lengthened membrane anchor escapes from the endoplasmic reticulum and reaches the plasma membrane. Proc Natl Acad Sci U S A. 1996;93: 4207–4212. 8633042 10.1073/pnas.93.9.4207 PMC39513

[75] Horie C, Suzuki H, Sakaguchi M, Mihara K. Characterization of Signal That Directs C-Tail-anchored Proteins to Mammalian Mitochondrial Outer Membrane. Mol Biol Cell. 2002;13: 1615–1625. 10.1091/mbc.01-12-0570 12006657 PMC111131

[76] D’Arrigo A, Manera E, Longhi R, Borgese N. The specific subcellular localization of two isoforms of cytochrome b5 suggests novel targeting pathways. J Biol Chem. 1993;268: 2802–2808. 8428954

[77] Kuroda R, Ikenoue T, Honsho M, Tsujimoto S, Mitoma J, Ito A. Charged Amino Acids at the Carboxyl-Terminal Portions Determine the Intracellular Locations of Two Isoforms of Cytochrome b5. J Biol Chem. 1998;273: 31097–31102. 10.1074/jbc.273.47.31097 9813010

[78] Borgese N, Gazzoni I, Barberi M, Colombo S, Pedrazzini E. Targeting of a Tail-anchored Protein to Endoplasmic Reticulum and Mitochondrial Outer Membrane by Independent but Competing Pathways. Mol Biol Cell. 2001;12: 2482–2496. 11514630 10.1091/mbc.12.8.2482 PMC58608

[79] Isenmann S, Khew-Goodall Y, Gamble J, Vadas M, Wattenberg BW. A Splice-Isoform of Vesicle-associated Membrane Protein-1 (VAMP-1) Contains a Mitochondrial Targeting Signal. Mol Biol Cell. 1998;9: 1649–1660. 10.1091/mbc.9.7.1649 9658161 PMC25402

[80] van Meer G, Voelker DR, Feigenson GW. Membrane lipids: where they are and how they behave. Nat Rev Mol Cell Biol. 2008;9: 112–124. 10.1038/nrm2330 18216768 PMC2642958

[81] Osman C, Voelker DR, Langer T. Making heads or tails of phospholipids in mitochondria. J Cell Biol. 2011;192: 7–16. 10.1083/jcb.201006159 21220505 PMC3019561

[82] Beilharz T, Egan B, Silver PA, Hofmann K, Lithgow T. Bipartite Signals Mediate Subcellular Targeting of Tail-anchored Membrane Proteins in Saccharomyces cerevisiae. J Biol Chem. 2003;278: 8219–8223. 10.1074/jbc.M212725200 12514182

[83] Kirchner J, Kam Z, Tzur G, Bershadsky AD, Geiger B. Live-cell monitoring of tyrosine phosphorylation in focal adhesions following microtubule disruption. J Cell Sci. 2003;116: 975–986. 10.1242/jcs.00284 12584242

[84] Offterdinger M, Georget V, Girod A, Bastiaens PIH. Imaging Phosphorylation Dynamics of the Epidermal Growth Factor Receptor. J Biol Chem. 2004;279: 36972–36981. 10.1074/jbc.M405830200 15215236

[85] Ozawa T, Natori Y, Sako Y, Kuroiwa H, Kuroiwa T, Umezawa Y. A Minimal Peptide Sequence That Targets Fluorescent and Functional Proteins into the Mitochondrial Intermembrane Space. ACS Chem Biol. 2007;2: 176–186. 10.1021/cb600492a 17348629

[86] Capaldi RA. Structure and assembly of cytochrome c oxidase. Arch Biochem Biophys. 1990;280: 252–262. 2164355 10.1016/0003-9861(90)90327-u

[87] Whitley P, Grahn E, Kutay U, Rapoport TA, Heijne G von. A 12-Residue-long Polyleucine Tail Is Sufficient to Anchor Synaptobrevin to the Endoplasmic Reticulum Membrane. J Biol Chem. 1996;271: 7583–7586. 10.1074/jbc.271.13.7583 8631791

[88] Pelham HRB. SNAREs and the Secretory Pathway—Lessons from Yeast. Exp Cell Res. 1999;247: 1–8. 10.1006/excr.1998.4356 10047442

[89] Janke C, Magiera MM, Rathfelder N, Taxis C, Reber S, Maekawa H, et al A versatile toolbox for PCR-based tagging of yeast genes: new fluorescent proteins, more markers and promoter substitution cassettes. Yeast. 2004;21: 947–962. 10.1002/yea.1142 15334558

[90] Walther K, Papke B, Sinn MB, Michel K, Kinkhabwala A. Precise measurement of protein interacting fractions with fluorescence lifetime imaging microscopy. Mol Biosyst. 2011;7: 322–336. 10.1039/c0mb00132e 21221430

[91] Offterdinger M, Bastiaens PI. Prolonged EGFR Signaling by ERBB2-Mediated Sequestration at the Plasma Membrane. Traffic. 2008;9: 147–155. 10.1111/j.1600-0854.2007.00665.x 17956594

[92] Pereira G, Tanaka TU, Nasmyth K, Schiebel E. Modes of spindle pole body inheritance and segregation of the Bfa1p–Bub2p checkpoint protein complex. EMBO J. 2001;20: 6359–6370. 10.1093/emboj/20.22.6359 11707407 PMC125717

[93] Khmelinskii A, Meurer M, Duishoev N, Delhomme N, Knop M. Seamless Gene Tagging by Endonuclease-Driven Homologous Recombination. PLoS ONE. 2011;6: e23794 10.1371/journal.pone.0023794 21915245 PMC3161820

[94] Knop M, Siegers K, Pereira G, Zachariae W, Winsor B, Nasmyth K, et al Epitope tagging of yeast genes using a PCR-based strategy: more tags and improved practical routines. Yeast. 1999;15: 963–972. 10.1002/(SICI)1097-0061(199907)15:10B<963::AID-YEA399>3.0.CO;2-W 10407276

[95] Hahn WC, Counter CM, Lundberg AS, Beijersbergen RL, Brooks MW, Weinberg RA. Creation of human tumour cells with defined genetic elements. Nature. 1999;400: 464–468. 10.1038/22780 10440377

[96] Schindelin J, Arganda-Carreras I, Frise E, Kaynig V, Longair M, Pietzsch T, et al Fiji: an open-source platform for biological-image analysis. Nat Methods. 2012;9: 676–682. 10.1038/nmeth.2019 22743772 PMC3855844

