# Correction: Subcellular Partitioning of Protein Tyrosine Phosphatase 1B to the Endoplasmic Reticulum and Mitochondria Depends Sensitively on the Composition of Its Tail Anchor

**DOI:** 10.1371/journal.pone.0154908

**Published:** 2016-04-28

**Authors:** Julia Fueller, Mikhail V. Egorov, Kirstin A. Walther, Ola Sabet, Jana Mallah, Markus Grabenbauer, Ali Kinkhabwala

A reference is omitted from the first sentence in the fifth paragraph of the Introduction. There is also an error in this sentence. The sentence should read: In this study, we report the following findings regarding PTP1B’s tail-anchor-mediated targeting to the ER and mitochondria and its role in mitochondrial signaling: (1) Endogenous PTP1B localizes to the mitochondria in multiple mammalian cell lines (see also O’Donovan et al., 2013), (2) PTP1B localizes to the outer mitochondrial membrane via its tail anchor, (3) Heterologous expression of PTP1B’s tail anchor in yeast reveals a potential minor role of the GET/TRC40 pathway in ER insertion, (4) Subcellular partitioning of PTP1B’s tail anchor is highly sensitive to its exact composition and (5) FLIM reveals an interaction of PTP1B with EGFR at the outer mitochondrial membrane.

The authors would like to inform readers that they mistakenly characterized their finding as "novel" that, in addition to cells extracted from rat brain tissue, endogenous PTP1B also “localizes to the mitochondria in multiple mammalian cell lines”. O’Donovan et al. (2013) had previously reported the mitochondrial localization of both endogenous and exogenously expressed PTP1B in chronic myeloid leukemia cell lines. O’Donovan et al. used subcellular fractionation followed by SDS-PAGE and western blot analysis to identify the mitochondrial pools of endogenous PTP1B as well as its phosphorylated form. They further microscopically confirmed the mitochondrial localization of PTP1B by exogenous expression of EGFR-PTP1B together with MitoTracker Fluor staining. While the current authors' results are not the first to show the mitochondrial localization of PTP1B in mammalian cell lines other than rat brain cells, the additional supporting evidence presented for COS-7, BJ Fibroblasts, HeLa, MCF7, MDCK, and HepG2 cell lines nevertheless serves to further generalize the findings of O'Donovan et al. (2013) regarding this mitochondrial pool of PTP1B.

The reference is: DS O’Donovan, S MacFhearraigh, J Whitfield, LB Swigart, GI Evan, MM Mc Gee. Sequential Cdk1 and Plk1 phosphorylation of Protein Tyrosine Phosphatase 1B promotes mitotic cell death. Cell Death and Disease. 2013; 4: e468.
