# Supplementary material for: Subcellular Partitioning of Protein Tyrosine Phosphatase 1B to the Endoplasmic Reticulum and Mitochondria Depends Sensitively on the Composition of Its Tail Anchor
Source: PLoS One. 2015 Oct 2;10(10):e0139429. doi: 10.1371/journal.pone.0139429 (PMC4592070; doi:10.1371/journal.pone.0139429)
Supplement: S1 Fig — COS-7 cells were prepared for immunostaining as in Fig 1 with the only difference being their additional expression of an ER marker (mTagBFP-Sec61). In the top row, the mitochondrial localization of endogenous PTP1B is revealed through its colocalization with the mitochondrial stain MitoTracker Red CMXRos (Invitrogen). In the second row and the third row (zoomed-in view of the white box in the second row), the discrepancy in the overlay of the PTP1B signal and the ER marker allows identification of membrane-bound PTP1B that is apparently not in proximity to the ER. The structure indicated with an arrow clearly colocalizes with the mitochondria in the top row but not with the ER in the second and third rows. Scale bars: 20 μm and 5 μm, respectively. (PDF) [file pone.0139429.s001.pdf]

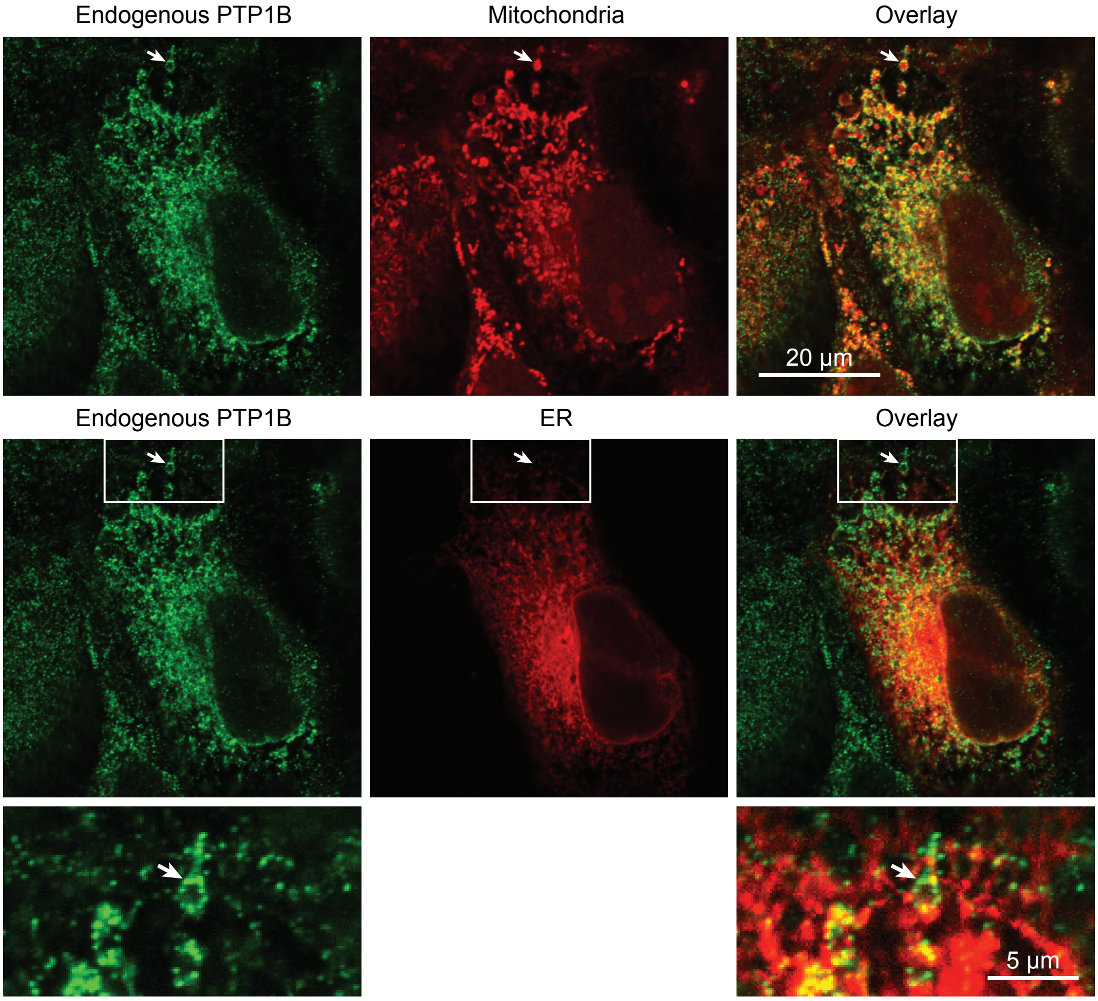

S1 Figure. Mitochondrial versus ER localization of endogenous PTP1B in COS-7 cells.
COS-7 cells were prepared for immunostaining as in Fig. 1 with the only difference being their additional expression of an ER marker (mTagBFP-Sec61). In the top row, the mitochondrial localization of endogenous PTP1B is revealed through its colocalization with the mitochondrial stain MitoTracker Red CMXRos (Invitrogen). In the second row and the third row (zoomed-in view of the white box in the second row), the discrepancy in the overlay of the PTP1B signal and the ER marker allows identification of membrane-bound PTP1B that is apparently not in proximity to the ER. The structure indicated with an arrow clearly colocalizes with the mitochondria in the top row but not with the ER in the second and third rows. Scale bars: 20 µm and 5 µm, respectively.
