# Supplementary material for: Subcellular Partitioning of Protein Tyrosine Phosphatase 1B to the Endoplasmic Reticulum and Mitochondria Depends Sensitively on the Composition of Its Tail Anchor
Source: PLoS One. 2015 Oct 2;10(10):e0139429. doi: 10.1371/journal.pone.0139429 (PMC4592070; doi:10.1371/journal.pone.0139429)
Supplement: S2 Fig — (A) COS-7 cells expressing mCitrine-PTP1B (green), mCherry-PTP1Btail (red) and Tom20-mTagBFP (cyan) were visualized by confocal microscopy. The lower right image represents the overlay of the mCitrine-PTP1B (green) and mCherry-PTP1Btail (red) images. As cells were transiently transfected, not all constructs were expressed in all cells (the cell occupying the upper left corner, for example, does not express the mCitrine-PTP1B construct). (B) COS-7 cells expressing mTFP1-PTP1Btail (green), mCherry-PTP1BtailVCFH (red) and Tom20-mTagBFP (cyan) were visualized by confocal microscopy. The lower right image represents the overlay of the mTFP1-PTP1Btail (green) and mCherry-PTP1BtailVCFH (red) images. Scale bars: 20 μm. (PDF) [file pone.0139429.s002.pdf]

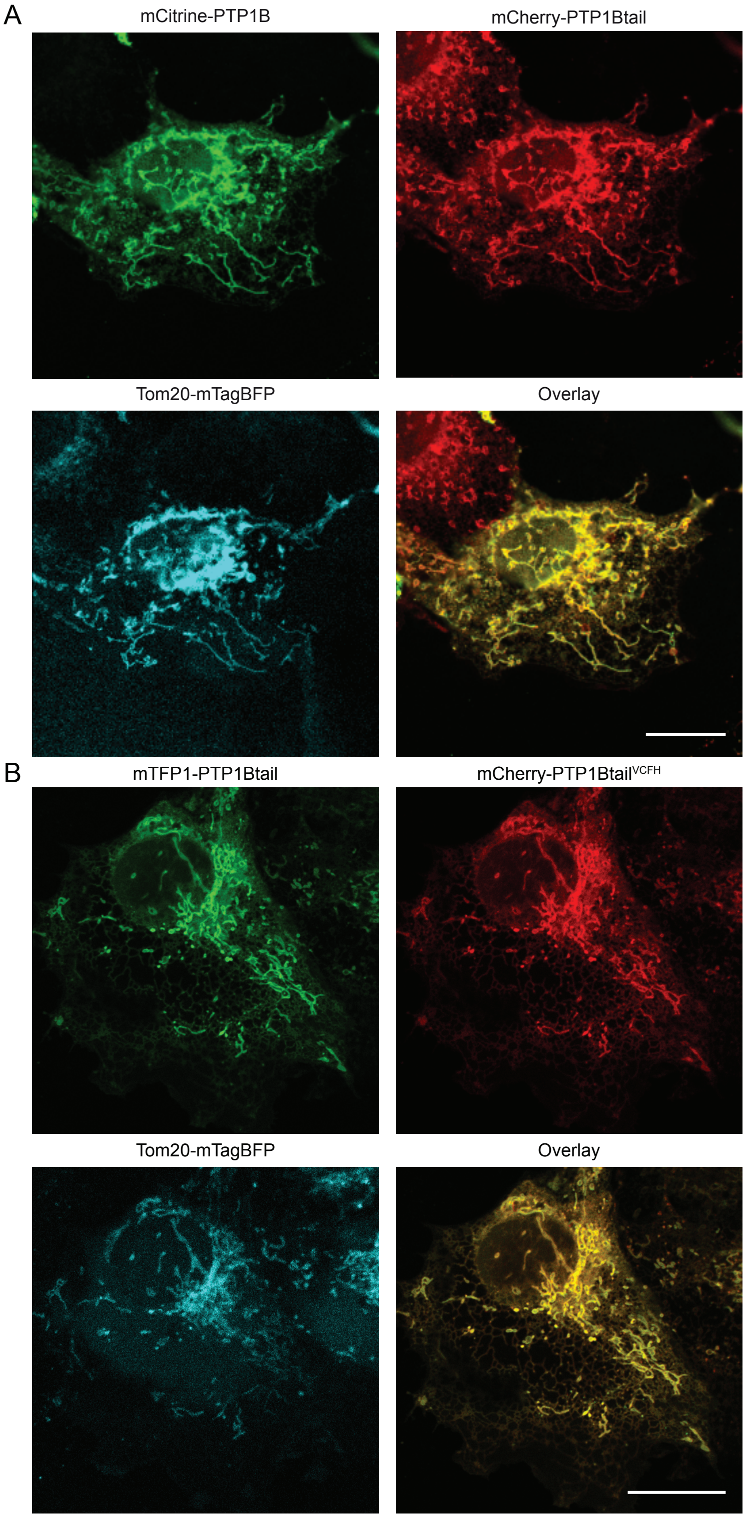

S2 Figure. Subcellular partitioning of PTP1B is completely determined by its tail anchor.
(A) COS-7 cells expressing mCitrine-PTP1B (green), mCherry-PTP1Btail (red) and Tom20-mTagBFP (cyan) were visualized by confocal microscopy. The lower right image represents the overlay of the mCitrine-PTP1B (green) and mCherry-PTP1Btail (red) images. As cells were transiently transfected, not all constructs were expressed in all cells (the cell occupying the upper left corner, for example, does not express the mCitrine-PTP1B construct). (B) COS-7 cells expressing mTFP1-PTP1Btail (green), mCherry-PTP1Btail^{VCFH} (red) and Tom20-mTagBFP (cyan) were visualized by confocal microscopy. The lower right image represents the overlay of the mTFP1-PTP1Btail (green) and mCherry-PTP1Btail^{VCFH} (red) images. Scale bars: 20 μm.
