# Supplementary material for: Subcellular Partitioning of Protein Tyrosine Phosphatase 1B to the Endoplasmic Reticulum and Mitochondria Depends Sensitively on the Composition of Its Tail Anchor
Source: PLoS One. 2015 Oct 2;10(10):e0139429. doi: 10.1371/journal.pone.0139429 (PMC4592070; doi:10.1371/journal.pone.0139429)
Supplement: S3 Fig — Confocal microscopy of a COS-7 cell expressing mCherry-PTP1Btail (green) and the ER lumenal marker mTFP1-ER (red). The overlay of both channels is also displayed. Especially clear regions of overlap (free from mitochondria) are highlighted (boxes). Scale bar: 10 μm. (PDF) [file pone.0139429.s003.pdf]

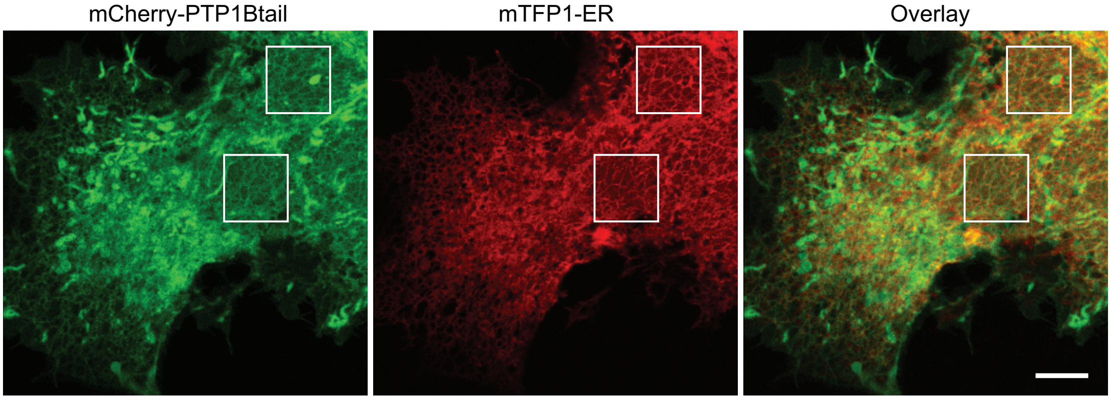

S3 Figure. Closer examination of the distribution of the PTP1B tail anchor along the ER. Confocal microscopy of a COS-7 cell expressing mCherry-PTP1Btail (green) and the ER lumenal marker mTFP1-ER (red). The overlay of both channels is also displayed. Especially clear regions of overlap (free from mitochondria) are highlighted (boxes). Scale bar: 10 μm.
