# Supplementary material for: Subcellular Partitioning of Protein Tyrosine Phosphatase 1B to the Endoplasmic Reticulum and Mitochondria Depends Sensitively on the Composition of Its Tail Anchor
Source: PLoS One. 2015 Oct 2;10(10):e0139429. doi: 10.1371/journal.pone.0139429 (PMC4592070; doi:10.1371/journal.pone.0139429)
Supplement: S4 Fig — (A) Overexpression of mTurquoise-APEX-PTP1B causes aggregation of mitochondrial and ER membranes. The large feature (upper left) is consistent with self-aggregation of the ER. Mitochondrial cristae (inner mitochondrial membrane) are not stained (black arrowhead). (B) Overexpression of mTurquoise-APEX-PTP1B leads as well to alteration of the mitochondrial interior in many mitochondria (black arrowhead). The white arrows indicate regions of higher staining along the ER that are in direct apposition to mitochondria (ER MAM sites). Scale bars: 500 nm. (PDF) [file pone.0139429.s004.pdf]

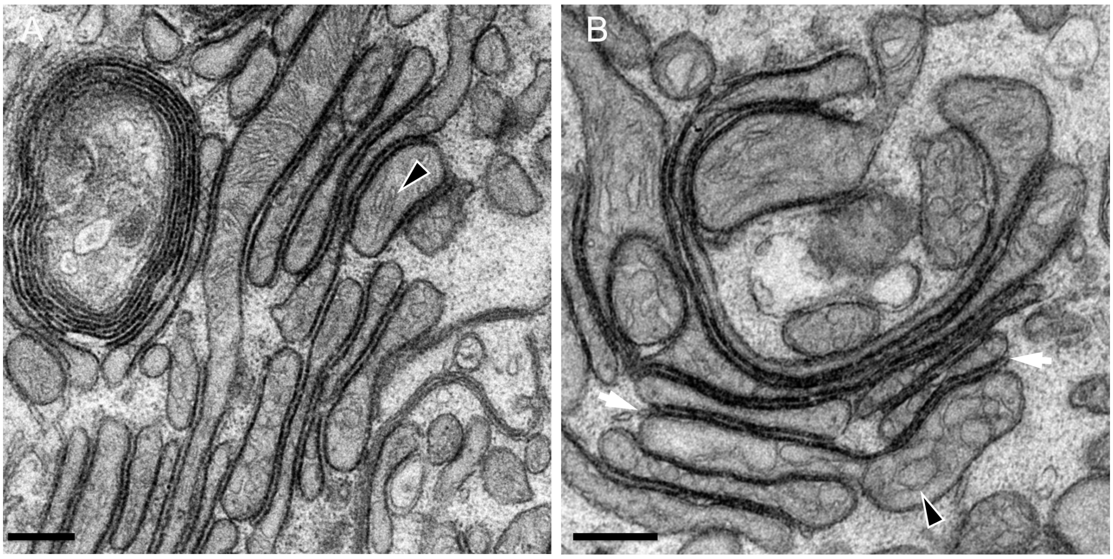

S4 Figure. Effects of PTP1B overexpression on ER and mitochondrial morphologies.
(A) Overexpression of mTurquoise-APEX-PTP1B causes aggregation of mitochondrial and ER membranes. The large feature (upper left) is consistent with self-aggregation of the ER. Mitochondrial cristae (inner mitochondrial membrane) are not stained (black arrowhead). (B) Overexpression of mTurquoise-APEX-PTP1B leads as well to alteration of the mitochondrial interior in many mitochondria (black arrowhead). The white arrows indicate regions of higher staining along the ER that are in direct apposition to mitochondria (ER MAM sites). Scale bars: 500 nm.
