# Supplementary material for: Subcellular Partitioning of Protein Tyrosine Phosphatase 1B to the Endoplasmic Reticulum and Mitochondria Depends Sensitively on the Composition of Its Tail Anchor
Source: PLoS One. 2015 Oct 2;10(10):e0139429. doi: 10.1371/journal.pone.0139429 (PMC4592070; doi:10.1371/journal.pone.0139429)
Supplement: S5 Fig — A library of previously identified C-terminal tail-anchor-containing proteins in yeast is displayed based on the list of Burri & Lithgow55 (with additional inclusion of Gem157 in the mitochondria panel) along with the list of 56 GPI-anchored proteins given in Ast et al.58, which were removed from the list of Burri & Lithgow due to their GPI anchorage. The hydropathies of the C termini of these proteins are displayed based on the Kyte-Doolittle method56 (boxcar smoothing of n = 7), with all protein sequences centered at the amino acid position corresponding to peak hydropathy. The hydropathy profile of the PTP1B tail anchor is shown in all figures (black). In the bottom right panel, the hydropathy profiles of canonical tail anchor proteins from yeast (Fis1, Ysy6, Sec22, Gas1) and from mammalian cells (Bcl2, CytB5, Sec61β, VAMP-1B) are shown, as well as the high hydropathy tail isoform PTP1BN412I (N412I, which has not been shifted to its maximum hydropathy, but is instead in register with the PTP1B tail). (PDF) [file pone.0139429.s005.pdf]

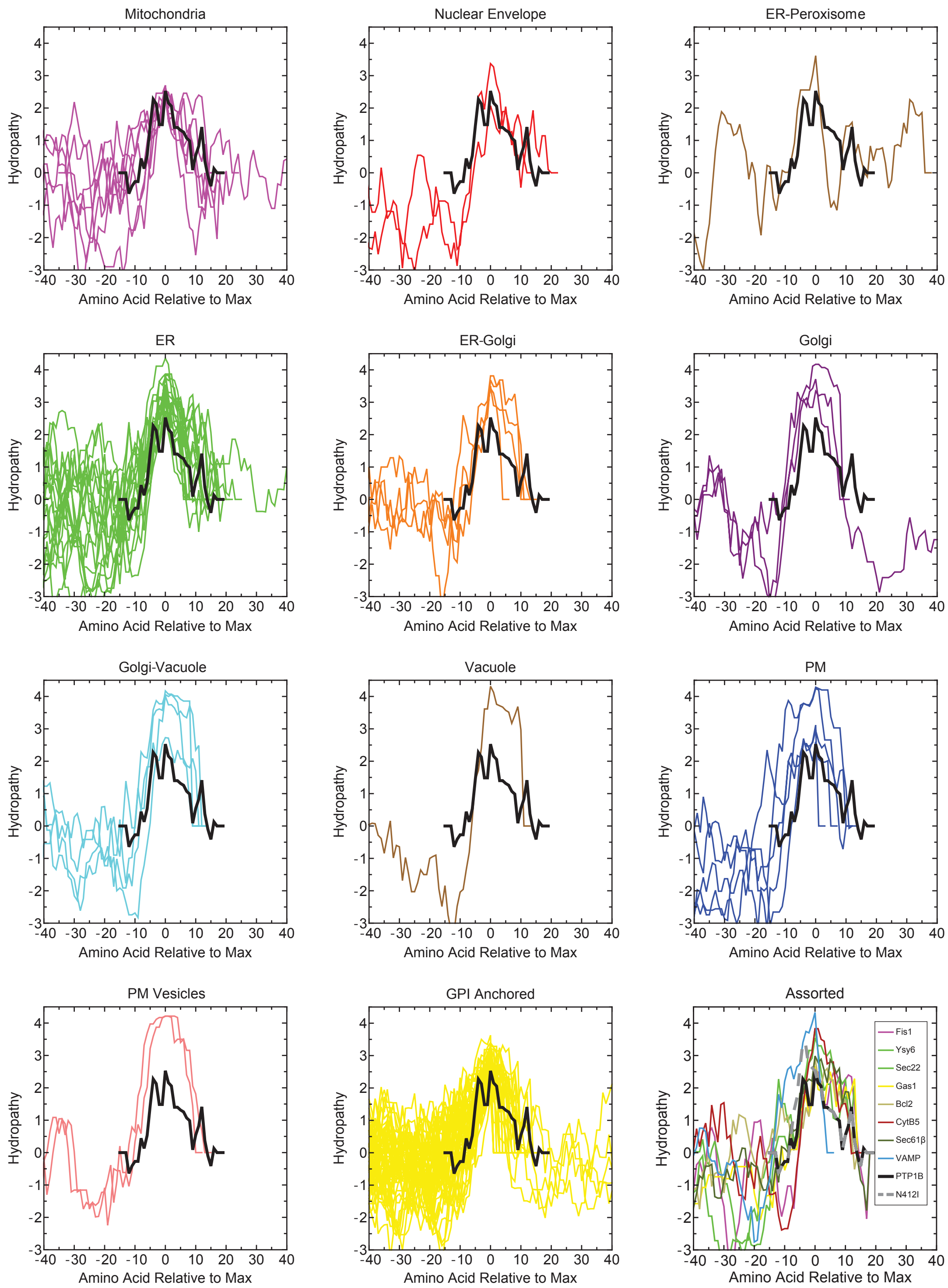

S5 Figure. Predicted targeting of the PTP1B tail anchor in yeast based on its hydropathy. A library of previously identified C-terminal tail-anchor-containing proteins in yeast is displayed based on the list of Burri & Lithgow[55] (with additional inclusion of Gem1[57] in the mitochondria panel) along with the list of 56 GPI-anchored proteins given in Ast et al.[58], which were removed from the list of Burri & Lithgow due to their GPI anchorage. The hydropathies of the C termini of these proteins are displayed based on the Kyte-Doolittle method[56] (boxcar smoothing of n=7), with all protein sequences centered at the amino acid position corresponding to peak hydropathy. The hydropathy profile of the PTP1B tail anchor is shown in all figures (black). In the bottom right panel, the hydropathy profiles of canonical tail anchor proteins from yeast (Fis1, Ysy6, Sec22, Gas1) and from mammalian cells (Bcl2, CytB5, Sec61β, VAMP-1B) are shown, as well as the high hydropathy tail isoform $PTP1B^{N412I}$ (N412I, which has not been shifted to its maximum hydropathy, but is instead in register with the PTP1B tail).
