# Supplementary material for: Subcellular Partitioning of Protein Tyrosine Phosphatase 1B to the Endoplasmic Reticulum and Mitochondria Depends Sensitively on the Composition of Its Tail Anchor
Source: PLoS One. 2015 Oct 2;10(10):e0139429. doi: 10.1371/journal.pone.0139429 (PMC4592070; doi:10.1371/journal.pone.0139429)
Supplement: S6 Fig — Confocal microscopy of specific strains of S. cerevisiae (ESM356-1 background strain[92]) that chromosomally express yemCitrine-PTP1Btail and markers for either the ER (Cwp2-mCherry), vacuole (Ste2-mCherry), mitochondria (Cox4-mCherry) or Golgi (Sec7-mCherry). Scale bar: 20 μm. (PDF) [file pone.0139429.s006.pdf]

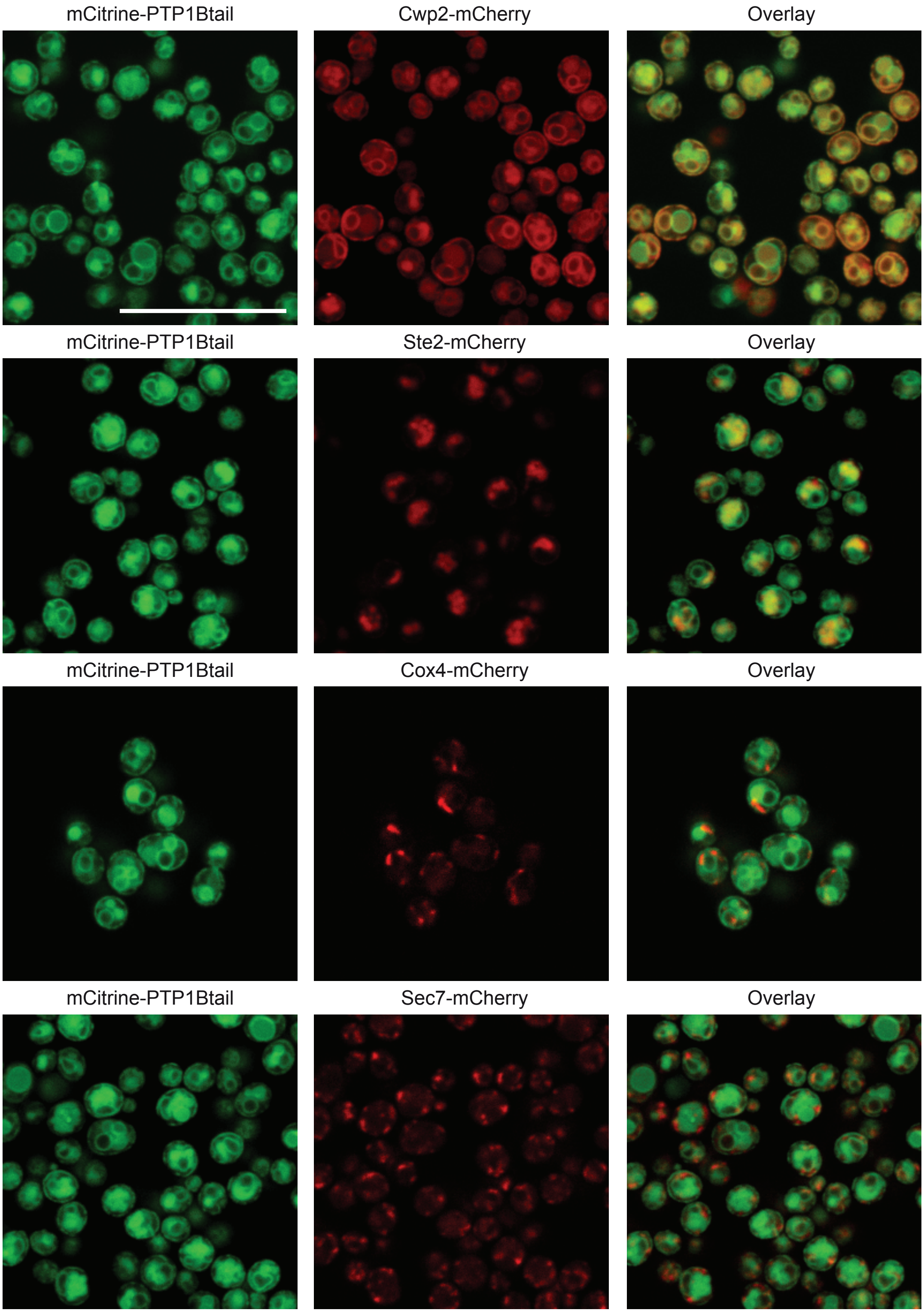

S6 Figure. Subcellular partitioning of the PTP1B tail anchor in yeast.
Confocal microscopy of specific strains of *S. cerevisiae* (ESM356-1 background strain[92]) that chromosomally express yemCitrine-PTP1Btail and markers for either the ER (Cwp2-mCherry), vacuole (Ste2-mCherry), mitochondria (Cox4-mCherry) or Golgi (Sec7-mCherry). Scale bar: 20 µm.
