# Supplementary material for: Subcellular Partitioning of Protein Tyrosine Phosphatase 1B to the Endoplasmic Reticulum and Mitochondria Depends Sensitively on the Composition of Its Tail Anchor
Source: PLoS One. 2015 Oct 2;10(10):e0139429. doi: 10.1371/journal.pone.0139429 (PMC4592070; doi:10.1371/journal.pone.0139429)
Supplement: S7 Fig — A wild-type strain of the yeast S. cerevisiae that produces ergosterol (RH288163) as well as a mutant strain that produces cholesterol as its dominant sterol (RH682963), with membrane composition therefore more similar to mammalian cells, were transformed with the plasmid p415-yemCitrine-PTP1Btail. In both strains, yemCitrine-PTP1Btail localized to the perinuclear/cortical membranes of the ER and to the vacuolar membrane. Scale bars: 5 μm. (PDF) [file pone.0139429.s007.pdf]

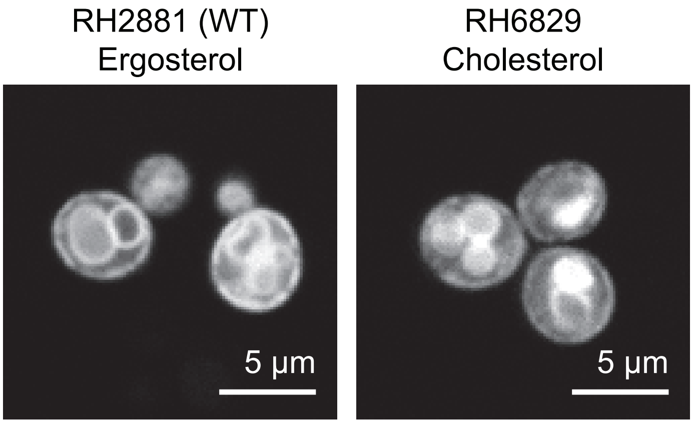

S7 Figure. Dependence of the targeting of the PTP1B tail anchor on the type of sterol.
A wild-type strain of the yeast *S. cerevisiae* that produces ergosterol (RH2881[63]) as well as a mutant strain that produces cholesterol as its dominant sterol (RH6829[63]), with membrane composition therefore more similar to mammalian cells, were transformed with the plasmid p415-yemCitrine-PTP1Btail. In both strains, yemCitrine-PTP1Btail localized to the perinuclear/cortical membranes of the ER and to the vacuolar membrane. Scale bars: 5 µm.
