# Supplementary material for: Subcellular Partitioning of Protein Tyrosine Phosphatase 1B to the Endoplasmic Reticulum and Mitochondria Depends Sensitively on the Composition of Its Tail Anchor
Source: PLoS One. 2015 Oct 2;10(10):e0139429. doi: 10.1371/journal.pone.0139429 (PMC4592070; doi:10.1371/journal.pone.0139429)
Supplement: S8 Fig — (A–E) Coexpression of the N-terminally truncated and fluorophore-labeled PTP1BtailC (Fig 4) in COS-7 cells (mCherry-PTP1BtailC) along with either the mitochondrial marker (left label, “Mito”) Tom20-mTagBFP (A) or the Golgi marker (“Golgi”) GalNAcT2-mTurquoise (B); and in yeast cells (yemCitrine-PTP1BtailC) along with either the ER marker (“ER”) Cwp2-mCherry (C), the mitochondrial marker (“Mito”) Cox4-mCherry (D) or the Golgi marker (“Golgi”) Sec7-mCherry (E). (F–J) Similar coexpression of the N- and C-terminally truncated and fluorophore-labeled PTP1BtailM (Fig 4) in COS-7 cells (mCherry-PTP1BtailM, F and G) and in yeast (yemCitrine-PTP1BtailM, H–J). Scale bars: 20 μm. (PDF) [file pone.0139429.s008.pdf]

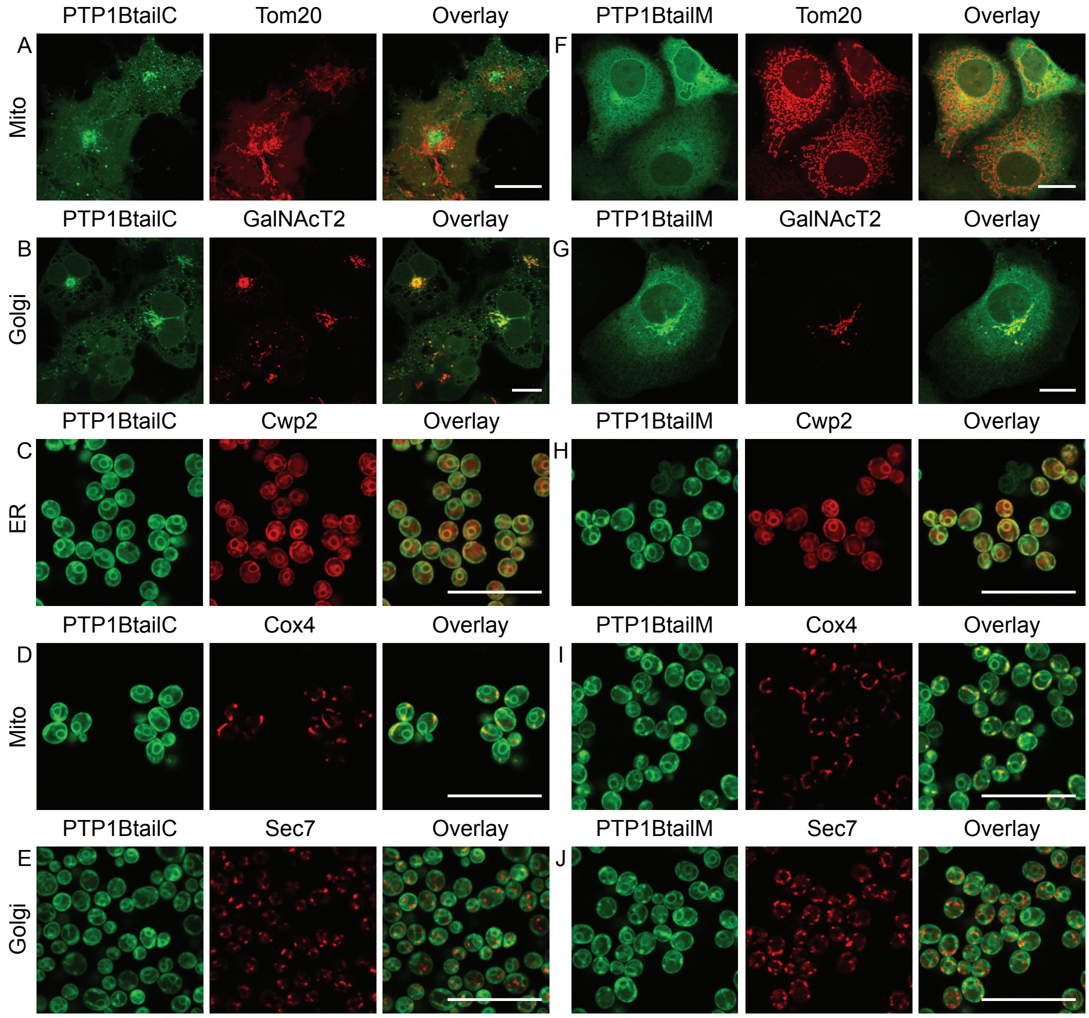

S8 Figure. Localization of N- and/or C-terminal truncations of the PTP1B tail anchor in COS-7 cells and in yeast.

(A–E) Coexpression of the N-terminally truncated and fluorophore-labeled PTP1BtailC (Fig. 4) in COS-7 cells (mCherry-PTP1BtailC) along with either the mitochondrial marker Tom20-mTagBFP (A) or the Golgi marker GalNAcT2-mTurquoise (B); and in yeast cells (yemCitrine-PTP1BtailC) along with either the ER marker Cwp2-mCherry (C), the mitochondrial marker Cox4-mCherry (D) or the Golgi marker Sec7-mCherry (E). (F–J) Similar coexpression of the N- and C-terminally truncated and fluorophore-labeled PTP1BtailM (Fig. 4) in COS-7 cells (mCherry-PTP1BtailM, F and G) and in yeast (yemCitrine-PTP1BtailM, H–J). Scale bars: 20 μm.
