# Supplementary material for: Subcellular Partitioning of Protein Tyrosine Phosphatase 1B to the Endoplasmic Reticulum and Mitochondria Depends Sensitively on the Composition of Its Tail Anchor
Source: PLoS One. 2015 Oct 2;10(10):e0139429. doi: 10.1371/journal.pone.0139429 (PMC4592070; doi:10.1371/journal.pone.0139429)
Supplement: S9 Fig — (A,B) Coexpression of the fluorphore-labeled, negatively charged tail isoform PTP1BtailR428E (Fig 4) in COS-7 cells (mCherry-PTP1BtailR428E) along with the mitochondrial marker (left label, “Mito”) Tom20-mTagBFP (A) and in yeast cells (yemCitrine-PTP1BtailR428E) along with the mitochondrial marker Cox4-mCherry (B). (C,D) Coexpression of the fluorophore-labeled, highly positively charged tail isoform PTP1BtailF429R (Fig 4) in COS-7 cells (mCherry-PTP1BtailF429R) along with the mitochondrial marker Tom20-mTagBFP (C) and in yeast cells (yemCitrine-PTP1BtailF429R) along with the mitochondrial marker Cox4-mCherry (D). Scale bars: 20 μm. (PDF) [file pone.0139429.s009.pdf]

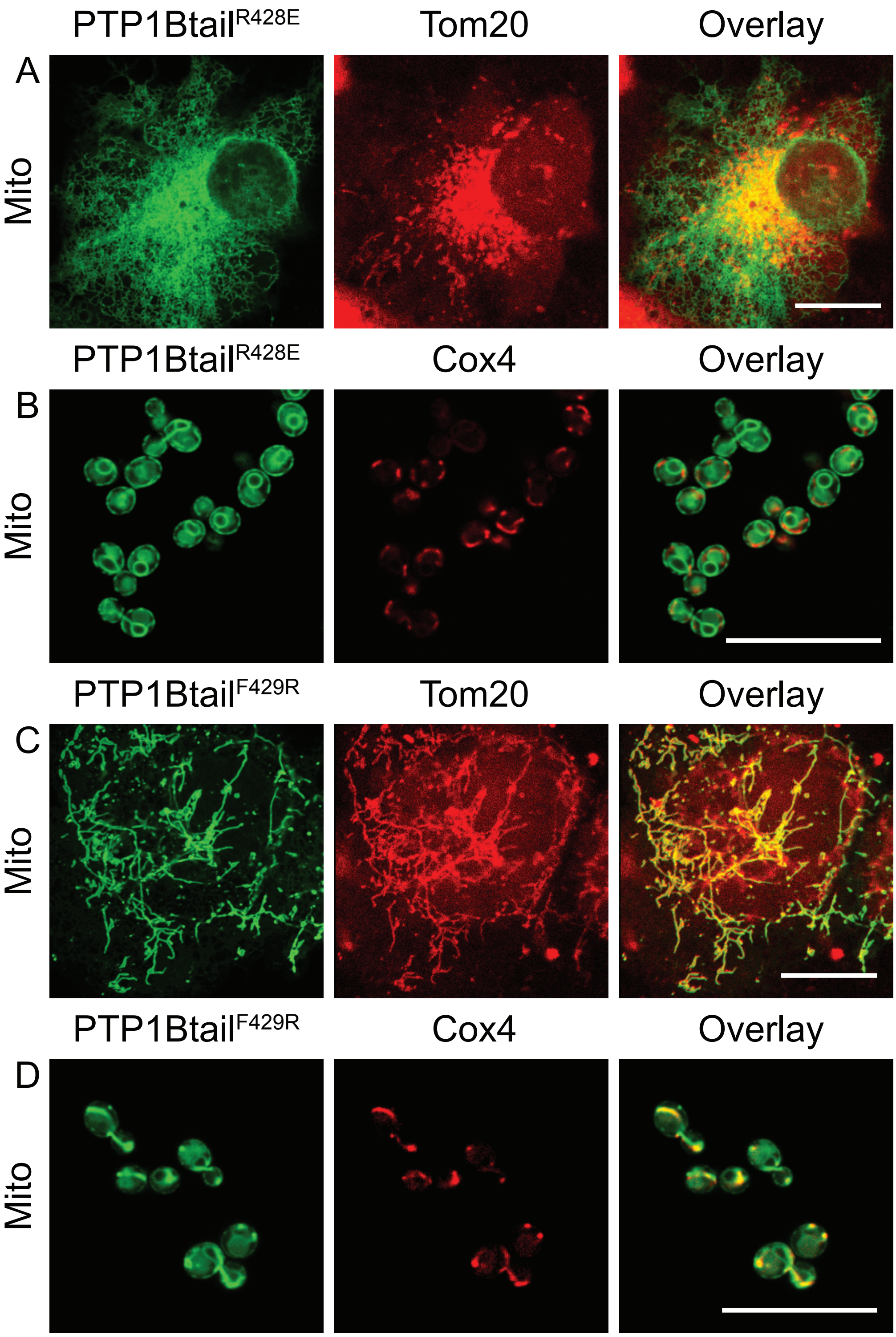

S9 Figure. Localization of charge-altered isoforms of the PTP1B tail anchor in COS-7 cells and in yeast.

(A,B) Coexpression of the fluorphore-labeled, negatively charged tail isoform PTP1Btail^{R428E} (Fig. 4) in COS-7 cells (mCherry-PTP1Btail^{R428E}) along with the mitochondrial marker Tom20-mTagBFP (A) and in yeast cells (yemCitrine-PTP1Btail^{R428E}) along with the mitochondrial marker Cox4-mCherry (B). (C,D) Coexpression of the fluorophore-labeled, highly positively charged tail isoform PTP1Btail^{F429R} (Fig. 4) in COS-7 cells (mCherry-PTP1Btail^{F429R}) along with the mitochondrial marker Tom20-mTagBFP (C) and in yeast cells (yemCitrine-PTP1Btail^{F429R}) along with the mitochondrial marker Cox4-mCherry (D). Scale bars: 20 µm.
