# Supplementary material for: Subcellular Partitioning of Protein Tyrosine Phosphatase 1B to the Endoplasmic Reticulum and Mitochondria Depends Sensitively on the Composition of Its Tail Anchor
Source: PLoS One. 2015 Oct 2;10(10):e0139429. doi: 10.1371/journal.pone.0139429 (PMC4592070; doi:10.1371/journal.pone.0139429)
Supplement: S10 Fig — The wild-type tail anchor chimera mTFP1-PTP1Btail is shown (green) alongside tail isoform chimera mCherry-PTP1BtailN412I having higher hydropathy (red) in COS-7 cells. Their red/green overlay is also displayed as well as the mitochondrial marker Tom20-mTagBFP. Scale bar: 20 μm. (PDF) [file pone.0139429.s010.pdf]

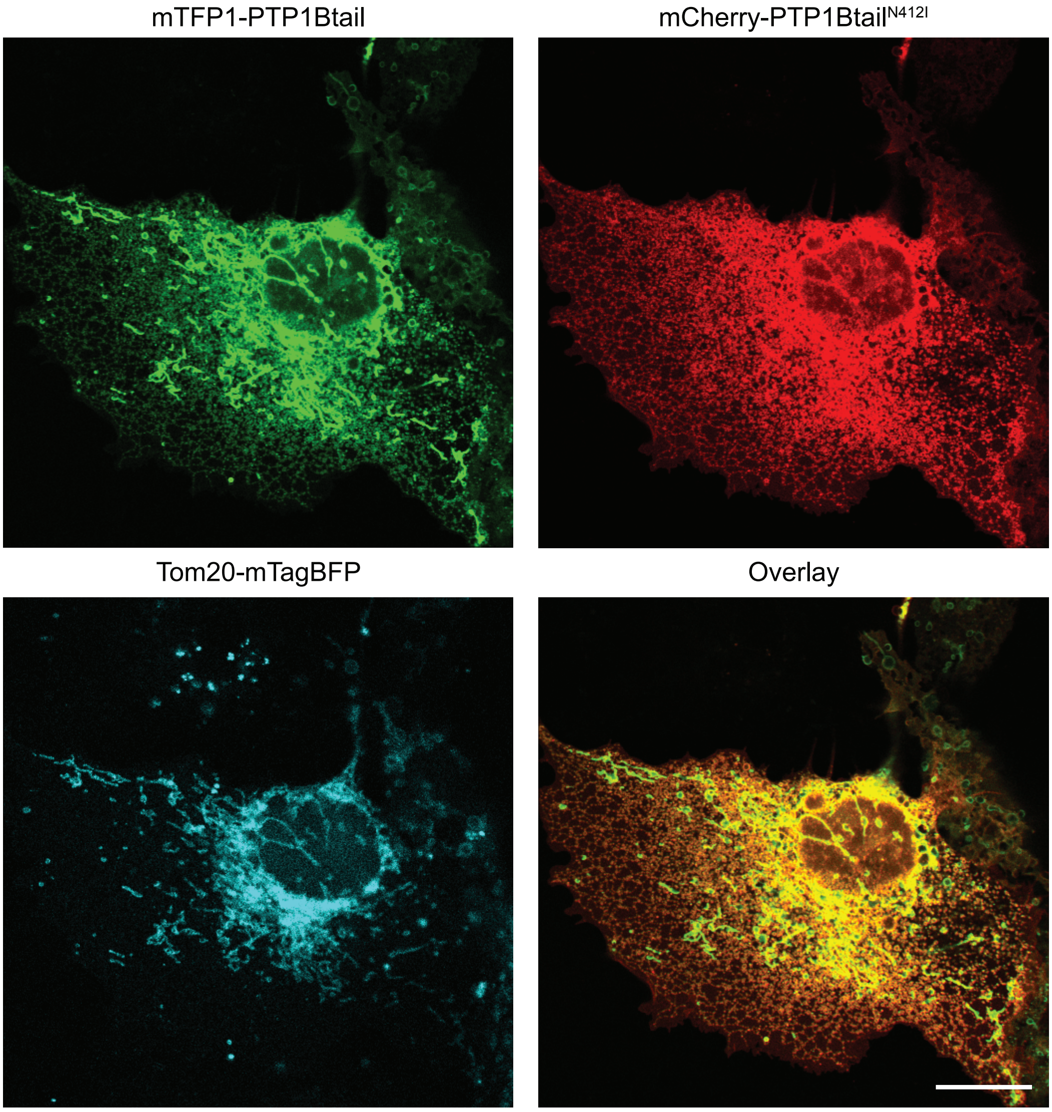

S10 Figure. Localization of a PTP1B tail isoform with high hydropathy in COS-7 cells.
The wild-type tail anchor chimera mTFP1-PTP1Btail is shown (green) alongside tail isoform chimera mCherry-PTP1Btail^{N412I} having higher hydropathy (red) in COS-7 cells. Their red/green overlay is also displayed as well as the mitochondrial marker Tom20-mTagBFP. Scale bar: 20 μm.
