# Supplementary material for: Subcellular Partitioning of Protein Tyrosine Phosphatase 1B to the Endoplasmic Reticulum and Mitochondria Depends Sensitively on the Composition of Its Tail Anchor
Source: PLoS One. 2015 Oct 2;10(10):e0139429. doi: 10.1371/journal.pone.0139429 (PMC4592070; doi:10.1371/journal.pone.0139429)
Supplement: S11 Fig — (A) Subcellular partitioning of a slightly truncated tail isoform PTP1BtailΔHALS (Fig 4, red) and the original tail anchor (green) were identical (see overlay), with both accumulating strongly at the mitochondria (as marked using Tom20-mTagBFP, cyan). Differences at the cell peripheries are due to optical refraction artifacts. The hydropathy profiles of the PTP1BtailΔHALS (black) and a scrambled isoform PTP1BtailScr (Fig 4, dashed magenta) are also shown. (B–C) Coexpression of the fluorophore-labeled PTP1BtailScr in COS-7 cells (mCherry-PTP1BtailScr) along with either the mitochondrial marker Tom20-mTagBFP (B) or the Golgi marker GalNAcT2-mTurquoise (C). Scale bars: 20 μm. (PDF) [file pone.0139429.s011.pdf]

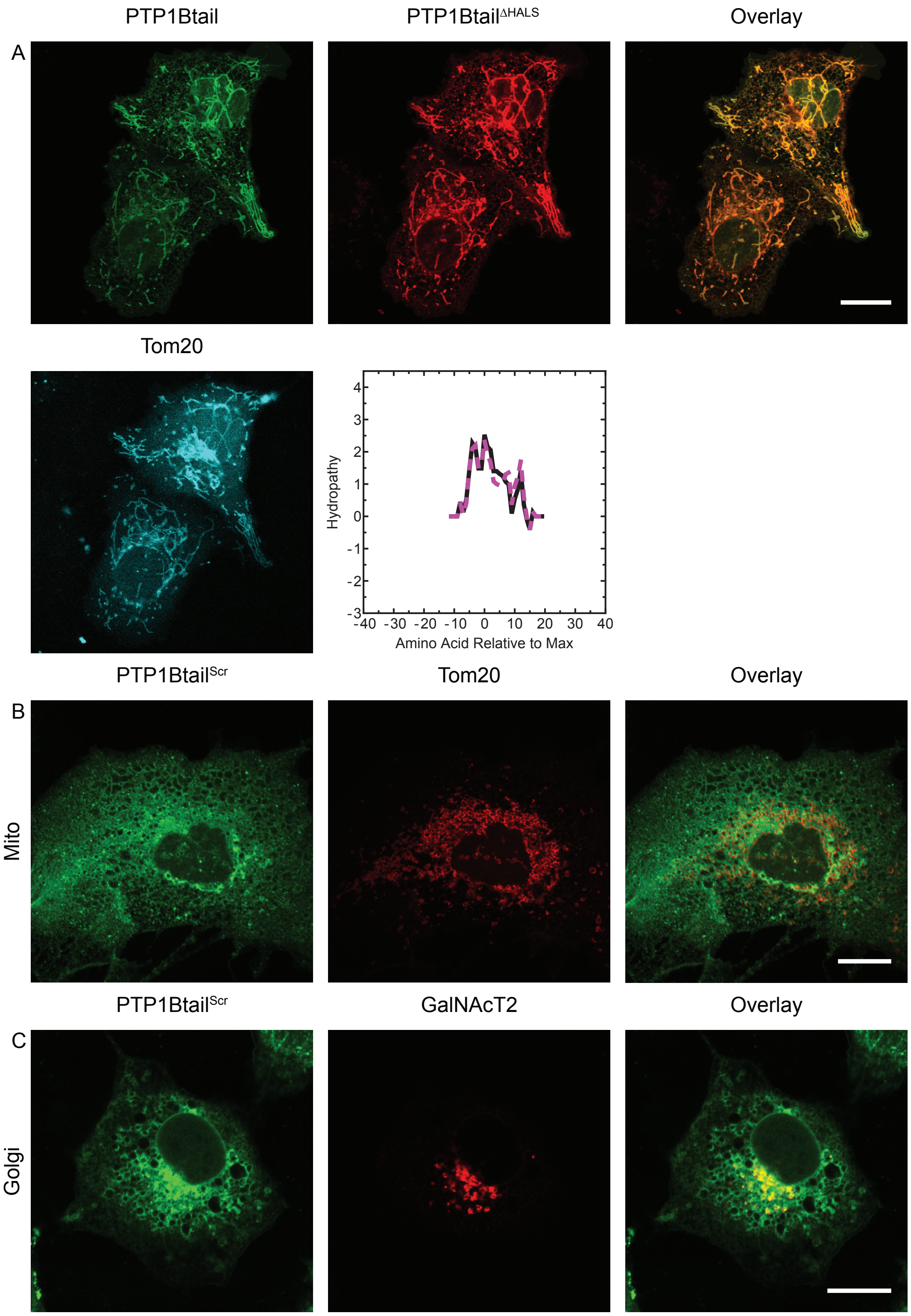

S11 Figure. Localization of a scrambled PTP1B tail isoform in COS-7 cells.
(A) Subcellular partitioning of a slightly truncated tail isoform PTP1Btail$^{\Delta HALS}$ (Fig. 4, red) and the original tail anchor (green) were identical (see overlay), with both accumulating strongly at the mitochondria (as marked using Tom20-mTagBFP, cyan). Differences at the cell peripheries are due to optical refraction artifacts. The hydropathy profiles of the PTP1Btail$^{\Delta HALS}$ (black) and a scrambled isoform PTP1Btail^{Scr} (Fig. 4, dashed magenta) are also shown. (B–C) Coexpression of the fluorophore-labeled PTP1Btail^{Scr} in COS-7 cells (mCherry-PTP1Btail^{Scr}) along with either the mitochondrial marker Tom20-mTagBFP (B) or the Golgi marker GalNAcT2-mTurquoise (C). Scale bars: 20 µm.
