# Supplementary material for: Subcellular Partitioning of Protein Tyrosine Phosphatase 1B to the Endoplasmic Reticulum and Mitochondria Depends Sensitively on the Composition of Its Tail Anchor
Source: PLoS One. 2015 Oct 2;10(10):e0139429. doi: 10.1371/journal.pone.0139429 (PMC4592070; doi:10.1371/journal.pone.0139429)
Supplement: S12 Fig — COS-7 cells expressing dSH2-YFP, PTB-mCherry and the mitochondrial marker Tom20-mTagBFP were starved and imaged before (0 min) and after (5 min and 20 min) their stimulation with EGF (100 ng/mL). No mitochondrial localization of dSH2-YFP was observed. In contrast, a basal accumulation of PTB-mCherry at the mitochondria was observed that increased following EGF stimulation. Scale bar: 20 μm. (PDF) [file pone.0139429.s012.pdf]

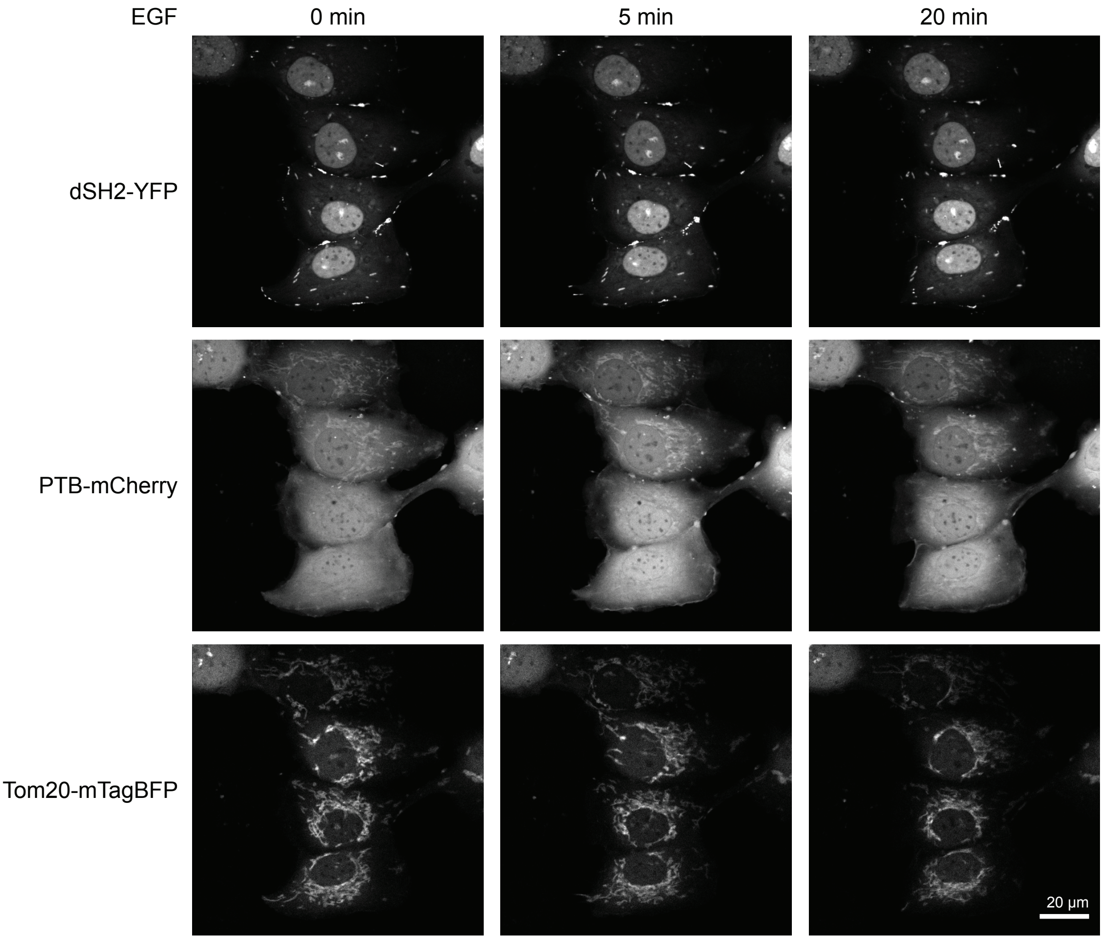

S12 Figure. Visualizing mitochondrial phosphotyrosine before and after EGF stimulation. COS-7 cells expressing dSH2-YFP, PTB-mCherry and the mitochondrial marker Tom20-mTagBFP were starved and imaged before (0 min) and after (5 min and 20 min) their stimulation with EGF (100 ng/mL). No mitochondrial localization of dSH2-YFP was observed. In contrast, a basal accumulation of PTB-mCherry at the mitochondria was observed that increased following EGF stimulation. Scale bar: 20 µm.
