# Supplementary material for: Subcellular Partitioning of Protein Tyrosine Phosphatase 1B to the Endoplasmic Reticulum and Mitochondria Depends Sensitively on the Composition of Its Tail Anchor
Source: PLoS One. 2015 Oct 2;10(10):e0139429. doi: 10.1371/journal.pone.0139429 (PMC4592070; doi:10.1371/journal.pone.0139429)
Supplement: S13 Fig — Donor lifetime images of ErbB1-mCitrine are shown before (-2 min to 0 min) and after (2 min to 4 min, 12 min to 14 min) stimulation with EGF in cells expressing the donor-labeled ErbB1 and the mitochondrial marker Tom20-mTagBFP in the first and second rows (lifetime control cells, representative of n = 3 recordings). In the column “Histogram/Acceptor”, the corresponding TCSPC histograms of the donor lifetime are shown (“Res” stands for the normalized fit residuals, see Walther et al. for further details90) and acceptor images are also displayed (acquired immediately after recording of the donor data). A lifetime of 3.02 ns was obtained from fitting the histogram of the entire 16-minute recording, assuming a fixed donor-only lifetime of 3.05 ns (blue TCSPC histogram) and a FRET lifetime of 1.51 ns (orange TCSPC histogram), which were determined from double lifetime fitting of the cells shown in the third and fourth rows. A generally low FRET fraction (consistent with zero) was obtained across the images. FLIM images of COS-7 cells expressing ErbB1-mCitrine, mCherry-PTP1BD/A and Tom20-mTagBFP are shown in the third and fourth rows (representative of n = 5 recordings). The image of acceptor mCherry-PTP1BD/A is given in the bottom row and fourth column (“Histogram/Acceptor”) to be compared with the Tom20-mTagBFP image to its right. Here, a basal interaction (of varying strength) is detected that increases in all of the cells after EGF stimulation. An average lifetime of 2.78 ns over the entire 16-minute movie was obtained, which was significantly lower than the 3.02 ns lifetime of the donor-only control cells. A specific decrease in lifetime at the mitochondria (arrows) was clearly observed in the cells on the right. The Tom20-mTagBFP mislocalized in the cell on the left (large aggregate) preventing determination of the mitochondria in this cell. Color scale in the top left image gives the FRET fraction α. The “intensity weighted” images of the donor in the second and f [file pone.0139429.s013.pdf]

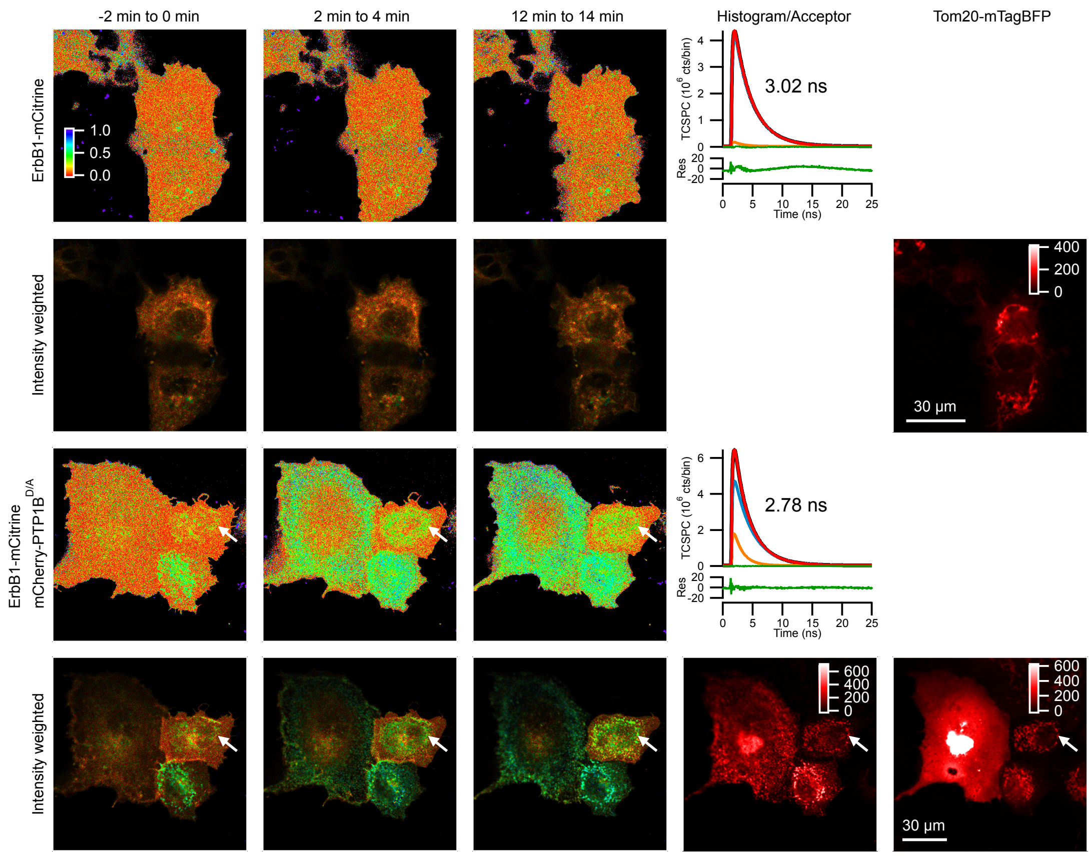

S13 Figure. Dynamic FLIM-based monitoring of the subcellular interaction of ErbB1-mCitrine with mCherry-PTP1B$^{D/A}$ in COS-7 cells following EGF stimulation.

Donor lifetime images of ErbB1-mCitrine are shown before (-2 min to 0 min) and after (2 min to 4 min, 12 min to 14 min) stimulation with EGF in cells expressing the donor-labeled ErbB1 and the mitochondrial marker Tom20-mTagBFP in the first and second rows (lifetime control cells, representative of n=3 recordings). In the column "Histogram/Acceptor", the corresponding TCSPC histograms of the donor lifetime are shown ("Res" stands for the normalized fit residuals, see Walther et al. for further details[90]) and acceptor images are also displayed (acquired immediately after recording of the donor data). A lifetime of 3.02 ns was obtained from fitting the histogram of the entire 16-minute recording, assuming a fixed donor-only lifetime of 3.05 ns (blue TCSPC histogram) and a FRET lifetime of 1.51 ns (orange TCSPC histogram), which were determined from double lifetime fitting of the cells shown in the third and fourth rows. A generally low FRET fraction (consistent with zero) was obtained across the images. FLIM images of COS-7 cells expressing ErbB1-mCitrine, mCherry-PTP1B$^{D/A}$ and Tom20-mTagBFP are shown in the third and fourth rows (representative of n=5 recordings). The image of acceptor mCherry-PTP1B$^{D/A}$ is given in the bottom row and fourth column ("Histogram/Acceptor") to be compared with the Tom20-mTagBFP image to its right. Here, a basal interaction (of varying strength) is detected that increases in all of the cells after EGF stimulation. An average lifetime of 2.78 ns over the entire 16-minute movie was obtained, which was significantly lower than the 3.02 ns lifetime of the donor-only control cells. A specific decrease in lifetime at the mitochondria (arrows) was clearly observed in the cells on the right. The Tom20-mTagBFP mislocalized in the cell on the left (large aggregate) preventing determination of the mitochondria in this cell. Color scale in the top left image gives the FRET fraction α. The "intensity weighted" images of the donor in the second and fourth rows correspond to an additional weighting of the respective images in the first and third rows by the observed donor counts in each pixel[90]. Scale bars: 30 µm.
