# Supplementary material for: Subcellular Partitioning of Protein Tyrosine Phosphatase 1B to the Endoplasmic Reticulum and Mitochondria Depends Sensitively on the Composition of Its Tail Anchor
Source: PLoS One. 2015 Oct 2;10(10):e0139429. doi: 10.1371/journal.pone.0139429 (PMC4592070; doi:10.1371/journal.pone.0139429)
Supplement: S14 Fig — Donor lifetime images of COS-7 cells expressing ErbB1-mCitrine (donor), mCherry-PTP1Btail (acceptor) and the mitochondrial marker Tom20-mTagBFP (representative of n = 3 recordings, see S13 Fig for further details). The average lifetime of the entire 16 minute recording was 2.98 ns. A generally low FRET fraction α across the cells was detected that was similar to the negative control shown in the first two rows of S13 Fig. Despite the similar localization and expression of the tail-only acceptor-labeled chimera, no lifetime reduction either before or after EGF stimulation was detectable of the donor-labeled ErbB1-mCitrine. This control, therefore, importantly demonstrates that the reduced lifetime in the bottom two rows of S13 Fig reflects the direct interaction of ErbB1-mCitrine with the catalytic domain of mCherry-labeled PTP1BD/A. Scale bar: 30 μm. (PDF) [file pone.0139429.s014.pdf]

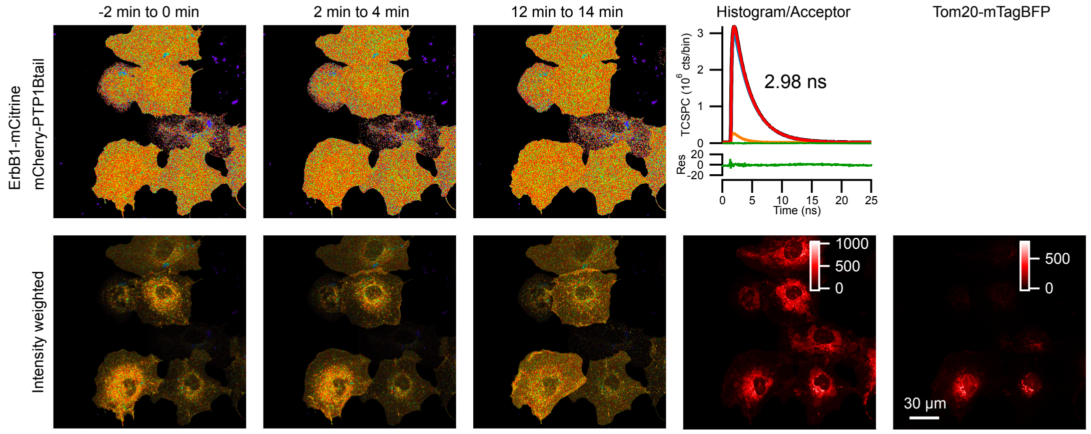

S14 Figure. Control for dynamic FLIM-based monitoring of subcellular ErbB1 interaction with PTP1B$^{D/A}$.

Donor lifetime images of COS-7 cells expressing ErbB1-mCitrine (donor), mCherry-PTP1Btail (acceptor) and the mitochondrial marker Tom20-mTagBFP (representative of n=3 recordings, see S13 Figure for further details). The average lifetime of the entire 16 minute recording was 2.98 ns. A generally low FRET fraction α across the cells was detected that was similar to the negative control shown in the first two rows of S13 Figure. Despite the similar localization and expression of the tail-only acceptor-labeled chimera, no lifetime reduction either before or after EGF stimulation was detectable of the donor-labeled ErbB1-mCitrine. This control, therefore, importantly demonstrates that the reduced lifetime in the bottom two rows of S13 Figure reflects the direct interaction of ErbB1-mCitrine with the catalytic domain of mCherry-labeled PTP1B$^{D/A}$. Scale bar: 30 µm.
