# Supplementary material for: Subcellular Partitioning of Protein Tyrosine Phosphatase 1B to the Endoplasmic Reticulum and Mitochondria Depends Sensitively on the Composition of Its Tail Anchor
Source: PLoS One. 2015 Oct 2;10(10):e0139429. doi: 10.1371/journal.pone.0139429 (PMC4592070; doi:10.1371/journal.pone.0139429)
Supplement: S15 Fig — In the first two rows, donor lifetime images of COS-7 cells expressing ErbB1-mCitrine, mCherry-PTP1BD/A-ER and the mitochondrial marker Tom20-mTagBFP are displayed before and after EGF stimulation (representative of n = 3 recordings, see S13 Fig for further details). A robust decrease in lifetime was detectable upon EGF stimulation, revealing the specific interaction of ErbB1 with ER-localized PTP1B. In the third and fourth rows, donor lifetime images of COS-7 cells expressing ErbB1-mCitrine, mCherry-PTP1BD/A-OMM and the mitochondrial marker Tom20-mTagBFP are displayed before and after EGF stimulation (representative of n = 4 recordings). A robust decrease in lifetime was detected upon EGF stimulation only in the vicinity of the mitochondria (arrows; note the high degree of overlap of the mCherry-PTP1BD/A-OMM construct and the mitochondrial marker Tom20-mTagBFP). Despite the only slight reduction of the lifetime of the entire 16 minute recording (2.89 ns for the first two rows, 2.96 ns for the last two rows), local lifetime reductions were clearly detected that coincided with the acceptor localization (ER in top two rows, mitochondria in bottom two rows). Scale bars: 30 μm. (PDF) [file pone.0139429.s015.pdf]

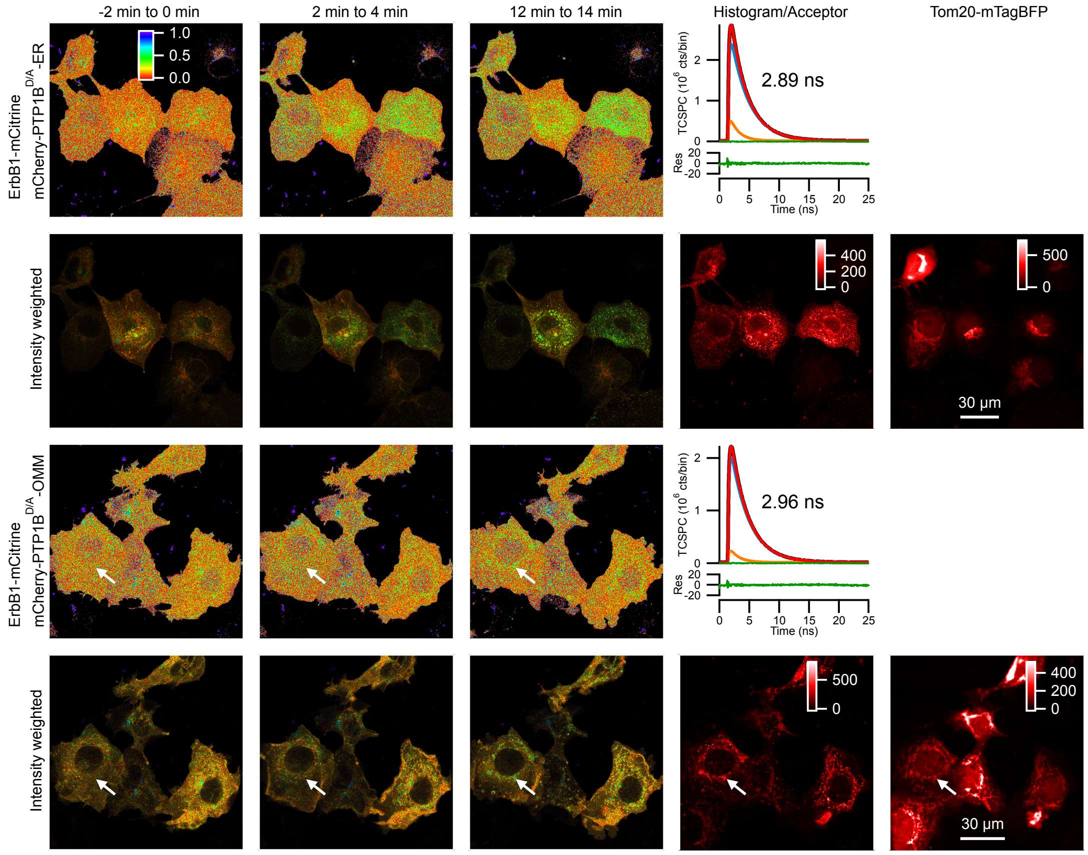

S15 Figure. Dynamic FLIM-based monitoring of the interaction of ErbB1-mCitrine with mCherry-labeled PTP1B$^{D/A}$ targeted to either the ER or the outer mitochondrial membrane.
In the first two rows, donor lifetime images of COS-7 cells expressing ErbB1-mCitrine, mCherry-PTP1B$^{D/A}$-ER and the mitochondrial marker Tom20-mTagBFP are displayed before and after EGF stimulation (representative of n=3 recordings, see S13 Figure for further details). A robust decrease in lifetime was detectable upon EGF stimulation, revealing the specific interaction of ErbB1 with ER-localized PTP1B. In the third and fourth rows, donor lifetime images of COS-7 cells expressing ErbB1-mCitrine, mCherry-PTP1B$^{D/A}$-OMM and the mitochondrial marker Tom20-mTagBFP are displayed before and after EGF stimulation (representative of n=4 recordings). A robust decrease in lifetime was detected upon EGF stimulation only in the vicinity of the mitochondria (arrows; note the high degree of overlap of the mCherry-PTP1B$^{D/A}$-OMM construct and the mitochondrial marker Tom20-mTagBFP). Despite the only slight reduction of the lifetime of the entire 16 minute recording (2.89 ns for the first two rows, 2.96 ns for the last two rows), local lifetime reductions were clearly detected that coincided with the acceptor localization (ER in top two rows, mitochondria in bottom two rows). Scale bars: 30 µm.
