# Supplementary material for: Subcellular Partitioning of Protein Tyrosine Phosphatase 1B to the Endoplasmic Reticulum and Mitochondria Depends Sensitively on the Composition of Its Tail Anchor
Source: PLoS One. 2015 Oct 2;10(10):e0139429. doi: 10.1371/journal.pone.0139429 (PMC4592070; doi:10.1371/journal.pone.0139429)
Supplement: S16 Fig — In the first two rows, donor lifetime images of COS-7 cells expressing ErbB1-mCitrine, mCherry-PTP1BD/A-IMS and the mitochondrial marker Tom20-mTagBFP are displayed before and after EGF stimulation (representative of n = 8 recordings, see S13 Fig for further details). No significant decrease in lifetime was detectable upon EGF stimulation, either generally across the cell or specifically at the mitochondria (arrows), and also no recruitment of ErbB1 to the mitochondria. In the third and fourth rows, donor lifetime images of COS-7 cells expressing ErbB1-mCitrine, mCherry-PTP1BD/A-MAT and the mitochondrial marker Tom20-mTagBFP are displayed before and after EGF stimulation (representative of n = 8 recordings). While there was a slightly decreased lifetime across some cells (reflected by the slightly lower lifetime of 2.95 ns obtained from fitting the entire histogram corresponding to the 16 minute recording), there was no additional decrease at the mitochondria (arrows) and also no observed recruitment of ErbB1 to the mitochondria. Scale bars: 30 μm. (PDF) [file pone.0139429.s016.pdf]

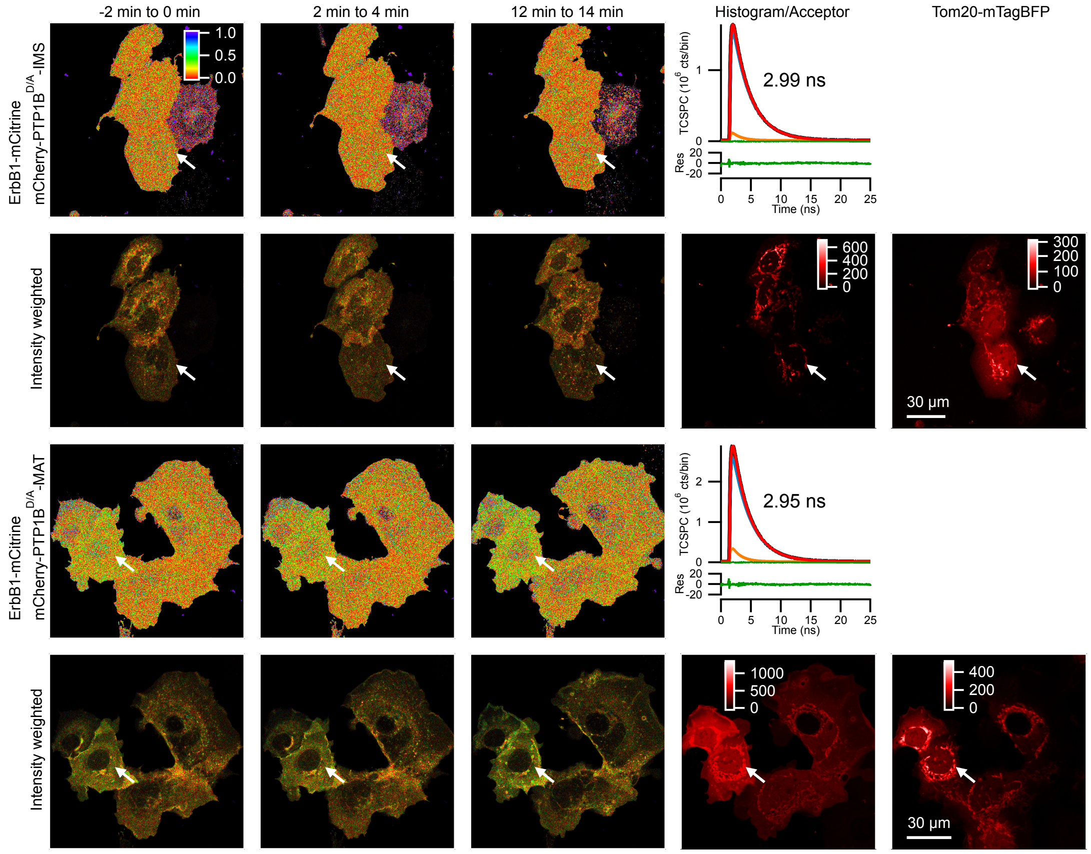

Fig S16. Dynamic FLIM-based monitoring of the interaction of ErbB1-mCitrine with mCherry-labeled PTP1B$^{D/A}$ targeted to either the intermembrane space (IMS) or the mitochondrial matrix (MAT).

In the first two rows, donor lifetime images of COS-7 cells expressing ErbB1-mCitrine, mCherry-PTP1B$^{D/A}$-IMS and the mitochondrial marker Tom20-mTagBFP are displayed before and after EGF stimulation (representative of n=8 recordings, see S13 Figure for further details). No significant decrease in lifetime was detectable upon EGF stimulation, either generally across the cell or specifically at the mitochondria (arrows), and also no recruitment of ErbB1 to the mitochondria. In the third and fourth rows, donor lifetime images of COS-7 cells expressing ErbB1-mCitrine, mCherry-PTP1B$^{D/A}$-MAT and the mitochondrial marker Tom20-mTagBFP are displayed before and after EGF stimulation (representative of n=8 recordings). While there was a slightly decreased lifetime across some cells (reflected by the slightly lower lifetime of 2.95 ns obtained from fitting the entire histogram corresponding to the 16 minute recording), there was no additional decrease at the mitochondria (arrows) and also no observed recruitment of ErbB1 to the mitochondria. Scale bars: 30 µm.
