# Supplementary material for: Subcellular Partitioning of Protein Tyrosine Phosphatase 1B to the Endoplasmic Reticulum and Mitochondria Depends Sensitively on the Composition of Its Tail Anchor
Source: PLoS One. 2015 Oct 2;10(10):e0139429. doi: 10.1371/journal.pone.0139429 (PMC4592070; doi:10.1371/journal.pone.0139429)
Supplement: S1 Table — (PDF) [file pone.0139429.s019.pdf]

Table S1. Localization in COS-7 (C) and yeast (Y) of different fluorophore-labeled PTP1B constructs.

	Mito	ER	Golgi	Vesicles	Vac	Cytosol
**PTP1Btail**	C	C Y			Y	
**PTP1Btail^{R428E}**		C Y			Y	
**PTP1Btail^{F429R}**	C Y	Y			Y	
**PTP1Btail^{N412I}**		C Y			Y	
**PTP1Btail$^{\Delta HALS}$**	C	C				
**PTP1Btail^{Scr}**		C	C	C		
**PTP1BtailC**	Y	C Y	C	C		C
**PTP1BtailM**	Y	C Y	C			C
