# Supplementary material for: Subcellular Partitioning of Protein Tyrosine Phosphatase 1B to the Endoplasmic Reticulum and Mitochondria Depends Sensitively on the Composition of Its Tail Anchor
Source: PLoS One. 2015 Oct 2;10(10):e0139429. doi: 10.1371/journal.pone.0139429 (PMC4592070; doi:10.1371/journal.pone.0139429)
Supplement: S2 Table — All strains with an AK prefix—and therefore not ESM356-192, RH288163 and RH682963—were generated for this study. (PDF) [file pone.0139429.s020.pdf]

Table S2. Yeast strains.
All strains with an AK prefix — and therefore not ESM356-1[92], RH2881[63] and RH6829[63] — were generated for this study.

strain	
ESM356-1	MAT**a** ura3-52 leu2Δ1 trp1Δ63 his3Δ200
AK199	ESM356-1 leu2Δ0::NatNT2rev-GPD-yemCitrine-PTP1Btail-Tcyc1
AK201	ESM356-1 leu2Δ0::NatNT2rev-GPD-yemCitrine-PTP1Btail-Tcyc1 STE2::mCherry::kanMX
AK202	ESM356-1 leu2Δ0::NatNT2rev-GPD-yemCitrine-PTP1Btail-Tcyc1 COX4::mCherry::kanMX
AK203	ESM356-1 leu2Δ0::NatNT2rev-GPD-yemCitrine-PTP1Btail-Tcyc1 SEC7::mCherry::kanMX
AK204	ESM356-1 leu2Δ0::NatNT2rev-GPD-yemCitrine-PTP1Btail-Tcyc1 CWP2::mCherry::kanMX
AK205	ESM356-1 leu2Δ0::NatNT2rev-GPD-yemCitrine-PTP1Btail-Tcyc1 get3Δ::klURA
AK206	ESM356-1 leu2Δ0::NatNT2rev-GPD-yemCitrine-PTP1Btail-Tcyc1 get2Δ::klURA
AK207	ESM356-1 leu2Δ0::NatNT2rev-GPD-yemCitrine-PTP1Btail-Tcyc1 sgt2Δ::klURA
AK222	ESM356-1 leu2Δ0::NatNT2rev-GPD-yemCitrine-PTP1Btail-Tcyc1 get2Δ::klURA get1Δ::HIS3MX6
AK225	ESM356-1 leu2Δ0::NatNT2rev-GPD-yemCitrine-PTP1Btail-Tcyc1 srp101Δ::klURA
AK238	ESM356-1 leu2Δ0::NatNT2rev-GPD-yemCitrine-YSY6tail-Tcyc1
AK256	ESM356-1 leu2Δ0::NatNT2rev-GPD-yemCitrine-YSY6tail-Tcyc1 get2Δ::klURA
AK257	ESM356-1 leu2Δ0::NatNT2rev-GPD-yemCitrine-PTP1BtailM-Tcyc1
AK258	ESM356-1 leu2Δ0::NatNT2rev-GPD-yemCitrine-PTP1BtailC-Tcyc1
AK259	ESM356-1 leu2Δ0::NatNT2rev-GPD-yemCitrine-PTP1Btail-Tcyc1 ydj1Δ::klURA
AK260	ESM356-1 leu2Δ0::NatNT2rev-GPD-yemCitrine-PTP1Btail-Tcyc1 ram1Δ::klURA
AK261	ESM356-1 leu2Δ0::NatNT2rev-GPD-yemCitrine-PTP1Btail-Tcyc1 apj1Δ::klURA
AK263	ESM356-1 leu2Δ0::NatNT2rev-GPD-yemCitrine-PTP1Btail-Tcyc1 sec72Δ::klURA
AK264	ESM356-1 leu2Δ0::NatNT2rev-GPD-yemCitrine-SEC22tail-Tcyc1
AK265	ESM356-1 leu2Δ0::NatNT2rev-GPD-yemCitrine-SEC22tail-Tcyc1 get2Δ::klURA
AK266	ESM356-1 leu2Δ0::NatNT2rev-GPD-yemCitrine-PTP1Btail-Tcyc1 get2Δ::klURA get1Δ::HIS3MX6 sec72Δ::hphNT1
AK273	ESM356-1 leu2Δ0::NatNT2rev-GPD-yemCitrine-PTP1BtailM-Tcyc1 SEC7::mCherry::kanMX
AK274	ESM356-1 leu2Δ0::NatNT2rev-GPD-yemCitrine-PTP1BtailC-Tcyc1SEC7::mCherry::kanMX
RH2881	MAT**a** ura3 leu2 his3 trp1 can1 bar1
AK275	RH2881 p415-yemCitrine-PTP1Btail
RH6829	MAT**a** ura3 leu2 his3 trp1 can1 bar1 erg5Δ::HIS5-TDH3-DHCR24 erg6Δ::TRP1-TDH3-DHCR7
AK276	RH6829 p415-yemCitrine-PTP1Btail
AK277	ESM356-1 leu2Δ0::NatNT2rev-GPD-yemCitrine-PTP1BtailR428E-Tcyc1
AK280	ESM356-1 leu2Δ0::NatNT2rev-GPD-yemCitrine-PTP1BtailN412I-Tcyc1
AK281	ESM356-1 leu2Δ0::NatNT2rev-GPD-yemCitrine-PTP1BtailF429R-Tcyc1
AK282	ESM356-1 leu2Δ0::NatNT2rev-GPD-yemCitrine-PTP1BtailR428E-Tcyc1 COX4::mCherry::kanMX
AK284	ESM356-1 leu2Δ0::NatNT2rev-GPD-yemCitrine-PTP1BtailF429R-Tcyc1 COX4::mCherry::kanMX
